# Efficacy of supermarket and web-based interventions for improving dietary quality: a randomized, controlled trial

**DOI:** 10.1038/s41591-022-02077-7

**Published:** 2022-12-01

**Authors:** Dylan L. Steen, Robert N. Helsley, Deepak L. Bhatt, Eileen C. King, Suzanne S. Summer, Matthew Fenchel, Brian E. Saelens, Mark H. Eckman, Sarah C. Couch

**Affiliations:** 1 Division of Cardiovascular Health and Disease, University of Cincinnati, Cincinnati, OH USA; 2 Department of Pharmacology and Nutritional Sciences, Saha Cardiovascular Research Center, University of Kentucky, Lexington, KY USA; 3 Mount Sinai Heart, Icahn School of Medicine at Mount Sinai Health System, New York, NY USA; 4 Cincinnati Children’s Hospital Medical Center, Cincinnati, OH USA; 5 University of Washington and Seattle Children’s Research Institute, Seattle, WA USA; 6 Division of General Internal Medicine, University of Cincinnati, Cincinnati, OH USA; 7 Department of Rehabilitation, Exercise and Nutrition Sciences, University of Cincinnati, Cincinnati, OH USA

**Keywords:** Outcomes research, Lifestyle modification, Public health, Randomized controlled trials, Risk factors

## Abstract

Dietary interventions may best be delivered at supermarkets, which offer convenience, accessibility, full food inventories and, increasingly, in-store registered dietitians, online shopping and delivery services. In collaboration with a large retail supermarket chain, we conducted a multisite supermarket and web-based intervention targeting nutrition trial (no. NCT03895580), randomizing participants (*n* = 247 (139 women and 108 men)) 2:2:1 to two levels of dietary education (Strategy 1 and Strategy 2) or an enhanced control group that included educational components beyond the routine standard of care. Both Strategies 1 and 2 included individualized, in-person, dietitian-led, purchasing data-guided interventions. Strategy 2 also included online tools for shopping, home delivery, selection of healthier purchases, meal planning and healthy recipes. The primary endpoint was change in dietary approaches to stop hypertension (DASH) score (a measure of adherence to the DASH diet) from baseline to 3 months. The primary endpoint was met because, at 3 months, the DASH score increased by 4.7 more for the combined Strategy 1 and Strategy 2 groups than for the control group (95% confidence interval (CI) (0.9, 8.5), *P* = 0.02). In a prespecified hierarchical test, at 3 months, DASH score increased by 3.8 more for the Strategy 2 group than for the Strategy 1 group (95% CI (0.8, 6.9), *P* = 0.01). This trial demonstrates the efficacy of data-guided, supermarket-based, dietary interventions and modern online shopping tools in improving dietary quality in a free-living, community-based population. The trial also demonstrates the opportunity for academic investigators to collaborate with retailers to design and rigorously test comprehensive healthcare interventions.

## Abstract (web summary)

In a randomized control trial, individuals who received dietitian-led counseling in the supermarket, together with web-based tools, had improved dietary quality in comparison to a control group, as assessed by adherence to the heart-healthy DASH diet plan.

## Main

Supermarkets with expansive footprints and evolving healthcare operations may offer new opportunities to expand clinical care services beyond traditional medical settings. Intrinsically, grocery stores may be partners well suited to addressing unmet public health challenges, including unhealthy diets^1^. Over the past 10 years, nutrition counseling provided by registered dietitians has been introduced in supermarket-based retail clinics^2,3^. New retail technologies may further address barriers to healthy eating, including websites and mobile applications for online food shopping, home grocery delivery and nutrition support. Automatically, electronically collected purchasing data, refined to create visibility into unhealthy dietary behaviors and intake, may also provide value when used by consumers and healthcare providers.

The supermarket and web-based intervention targeting nutrition (SuperWIN) was designed to test two in-person, dietitian-led education interventions focused on the DASH dietary pattern^4,5^. Each intervention was guided and individualized by data on each participant’s food purchases. The first intervention focused on the in-store shopping environment, while the second added online shopping, home delivery and other technologies that might improve the quality of grocery purchases and dietary intake. To date, research collaborations between academia and supermarkets and grocery stores have been very limited in regard to rigorous testing of new strategies aimed at improving dietary quality.

## Results

**Fig. 1:**
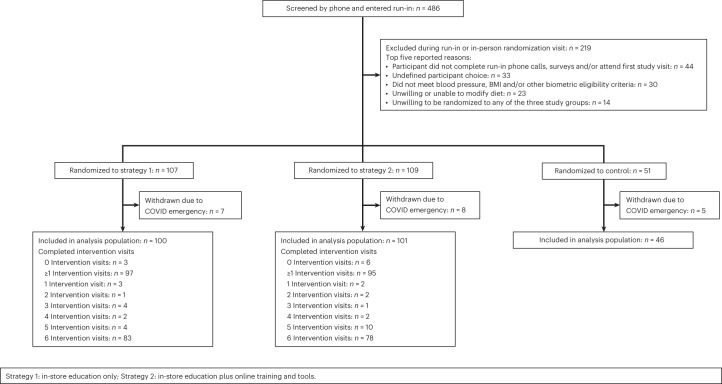
Trial profile: flow of participants through the SuperWIN trial. Due to the COVID-19 pandemic, recruitment and all in-person study visits were stopped on 13 March 2020. Under safety precautions, recruitment and study visits were resumed in June 2020.

From March 2019 to the end of February 2021, 267 participants in total were randomized (Fig. 1). Due to the COVID-19 pandemic, the study was temporarily interrupted due to safety concerns about continued in-person intervention delivery. Study leadership withdrew 20 recently randomized participants who had not yet reached 3 months of follow-up (that is, the time point at which data required for the primary endpoint were collected). We prespecified that these participants would be excluded from all subsequent analyses. The overall cohort, including for assessment of the primary endpoint, consisted of 247 participants. For subsequent prespecified COVID-19 analyses, the prepandemic subgroup consisted of 109 participants.

In the overall cohort, 91% of participants provided 3-month dietary intake data. In Strategies 1 and 2, 96% of participants attended one or more intervention visits, with 80% attending all six scheduled intervention visits. To understand the disruption due to the pandemic, study conduct is also reported separately for the prepandemic and pandemic cohorts (Supplementary Tables 8 and 9). In the prepandemic cohort (*n* = 109), intervention visit completion was 97.8% (264 of 270 total visits) in Strategy 1 and 98.4% (248 of 252 total visits) in Strategy 2. In the pandemic cohort (*n* = 138), intervention visit completion dropped to 84.5% (279 of 330 total visits) in Strategy 1 and to 81.1% (287 of 354) in Strategy 2. Prepandemic, dietary data and biometrics collection at 3 months was 100.0% (109 of 109) and 100.0% (109 of 109), respectively. In the pandemic cohort, dietary data and biometrics collection at 3 months dropped to 84.1% (116 of 138) and 79.0% (109 of 138), respectively.

### Participant characteristics

At baseline in the overall cohort, median age was 58 years, 69% of which were female and 21% self-identified as black or African American. In addition, 64.8% were married or living with a partner, 53.4% were employed full-time and 63.6% had at least a bachelor’s degree. Mean systolic blood pressure (SBP) was 129.3 (standard deviation (s.d.), 16.7) and hypertensive medication use was 72.9%. Obesity was present in 63.6%. Median non-high-density lipoprotein cholesterol (non-HDL-C) was 111 (min, 36; max, 216) and hypercholesterolemia medication use was 41.7%. Baseline characteristics were balanced across the groups (Table 1).

**Table 1:** Baseline characteristics by randomization group variable

	Control (*n* = 46)	Strategy 1 (*n* = 100)	Strategy 2 (*n* = 101)	Strategies 1 and 2 (*n* = 201)
Age, mean, years (s.d.)	56.2 (11.4)	57.0 (10.7)	55.8 (11.0)	56.4 (10.9)
Female, *n*	32 (69.6%)	68 (68.0%)	71 (70.3%)	139 (69.2%)
Race, *n*				
Black or African American	6 (13.0%)	23 (23.0%)	22 (21.8%)	45 (22.4%)
White	36 (78.3%)	73 (73.0%)	72 (71.3%)	145 (72.1%)
Married/living with partner, *n*	30 (65.2%)	70 (70.0%)	60 (59.4%)	130 (64.7%)
Employed full-time (≥40 h per week), *n*	25 (54.3%)	60 (60.0%)	47 (46.5%)	107 (53.2%)
Education, *n*				
Bachelor’s degree	17 (37.0%)	36 (36.0%)	31 (30.7%)	67 (33.3%)
Graduate degree	14 (30.4%)	27 (27.0%)	32 (31.7%)	59 (29.4%)
Annual household income, *n*				
<US$35,000	1 (2.2%)	6 (6.0%)	8 (7.9%)	14 (7.0%)
US$35,000 to <US$50,000	5 (10.9%)	8 (8.0%)	12 (11.9%)	20 (10.0%)
US$50,000 to <US$75,000	7 (15.2%)	24 (24.0%)	17 (16.8%)	41 (20.4%)
US$75,000 to <US$100,000	16 (34.8%)	15 (15.0%)	13 (12.9%)	28 (13.9%)
US$100,000 to <US$125,000	4 (8.7%)	10 (10.0%)	11 (10.9%)	21 (10.4%)
≥US$125,000	13 (28.3%)	37 (37.0%)	40 (39.6%)	77 (38.3%)
No children living in the household, *n*	35 (76.1%)	75 (75.0%)	77 (76.2%)	152 (75.6%)
People the participant shops/cooks for at home, including the participant, *n*				
1 or 2	32 (69.6%)	68 (68.0%)	71 ((70.3%)	139 (69.2%)
≥3	14 (30.4%)	32 (32.0%)	30 (29.7%)	62 (30.8%)
Previous myocardial infarction or stroke, *n*	5 (10.9%)	7 (7.0%)	5 (5.0%)	12 (6.0%)
Treated with hypertension meds, *n*	31 (67.4%)	77 (77.0%)	73 (72.3%)	150 (74.6%)
Blood pressure, mean, mmHg (s.d.)				
Systolic	130.0 (16.4)	129.8 (18.6)	128.4 (14.9)	129.1 (16.8)
Diastolic	85.7 (11.1)	82.1 (11.6)	83.4 (10.4)	82.8 (11.0)
Body mass index, mean, kg m^–2^ (s.d.)	33.8 (7.2)	34.0 (7.9)	32.9 (8.1)	33.5 (8.0)
Treatment with hypercholesterolemia meds, *n*	20 (43.5%)	47 (47.0%)	37 (36.6%)	136 (67.7%)
Non-HDL-C, mean, mg dl^–1^ (s.d.)	107.0 (32.5)	115.2 (37.0)	112.5 (35.3)	113.9 (36.1)
Total cholesterol, mean, mg dl^–1^ (s.d.)	158.5 (40.0)	167.4 (41.2)	168.1 (40.7)	167.8 (40.9)
TG, mean, mg dl^–1^ (s.d.)	170.5 (84.1)	173.0 (95.3)	159.2 (96.2)	166.0 (95.8)

### Primary endpoint, DASH score

The baseline mean DASH score was 45.4 (10.4), indicating that participants had on average approximately 50% adherence to the DASH dietary pattern goals. From baseline to 3 months, there were mean increases in DASH score of 5.8 (95% CI (2.5, 9.2), *P* = 0.0006), 8.6 (95% CI (6.4, 10.8), *P* < 0.0001) and 12.4 (95% CI (10.3, 14.6), *P* < 0.0001) for control, Strategy 1 and Strategy 2 groups, respectively (Table 2). From baseline to 3 months, the combined Strategies 1 and 2 showed a DASH score increased by 10.5 (95% CI (9.0, 12.1), *P* < 0.0001). The combined Strategies 1 and 2 resulted in a significant increase in DASH score by 4.7 (95% CI (0.9, 8.5), *P* = 0.02) compared with control (enhanced medical nutrition therapy) (Table 3). At 6 months (3 months post intervention), there were persistent increases in DASH score of 4.4 (95% CI (0.6, 8.1), *P* = 0.02), 6.6 (95% CI (4.0, 9.2), *P* < 0.0001) and 8.4 (95% CI (5.9, 11.0), *P* < 0.0001) for control, Strategy 1,and Strategy 2 groups, respectively (Table 2). At 6 months, the combined Strategies 1 and 2 had a persistently increased DASH score of 7.5 (95% CI (5.7, 9.3), *P* < 0.0001). However, the combined Strategies 1 and 2 no longer demonstrated a difference in DASH score (3.1, 95% CI (−1.0, 7.3), *P* = 0.14) compared with the enhanced control group (Table 3).

**Table 2:** Change in DASH score in control, Strategy 1 and Strategy 2 groups

DASH score	Control (*n* = 46) mean (95% CI)	Strategy 1 (*n* = 100) mean (95% CI)	Strategy 2 (*n* = 101) mean (95% CI)	Strategies 1 and 2 (*n* = 201) mean (95% CI)
**At baseline**	45.2 (42.0, 48.4)	44.4 (42.0, 46.8)	43.2 (40.8, 45.5)	43.8 (41.9, 45.7)
**At 3 months**	51.0 (47.6, 54.4)	53.1 (50.6, 55.5)	55.6 (53.2, 58.1)	54.3 (52.4, 56.3)
**At 6 months**	49.6 (46.3, 52.8)	51.0 (48.6, 53.5)	51.6 (49.2, 54.0)	51.3 (49.4, 53.2)
**Change (baseline to 3 months)**	5.8 (2.5, 9.2)	8.6 (6.4, 10.8)	12.4 (10.3, 14.6)	10.5 (9.0, 12.1)
**Change (baseline to 6 months)**	4.4 (0.6, 8.1)	6.6 (4.0, 9.2)	8.4 (5.9, 11.0)	7.5 (5.7, 9.3)

Strategy 1: in-store education only; Strategy 2: in-store education plus online training and tools. A mixed model for repeated measures was used to model each outcome, controlling for age group, sex, household size, race, income and baseline body mass index. Contrasts (with two-sided *t*-tests) were used to estimate differences between treatment groups for 3- and 6-month changes from baseline. Intervention effect is mean change in outcome and 95% CI. No adjustments for multiple comparison were made. These criteria apply also to Tables 3 and 4.

**Table 3:** Between-group differences in DASH score, primary and secondary comparisons

DASH score	Strategies 1 and 2 versus control mean (95% CI)	*P* value^a^	Strategy 2 versus strategy 1 mean (95% CI)	*P* value^b^
Change (baseline to 3 months)	4.7 (0.9, 8.5)	0.02	3.8 (0.8, 6.9)	0.01
Change (baseline to 6 months)	3.1 (−1.0, 7.3)	0.14	1.8 (−1.9, 5.5)	0.34

Intervention effect is difference in mean change in outcome and 95% confidence interval.

^a^*P* value from baseline to follow-up time point, whether Strategies 1 and 2 combined increased DASH score compared with control.

^b^*P* value from baseline to follow-up time point, whether Strategy 2 increased DASH score compared with Strategy 1.

We formally tested two hypotheses in hierarchical fashion: (1) from baseline to 3 months, whether Strategies 1 and 2 combined increased DASH score compared with control^a^; and (2) from baseline to 3 months, whether Strategy 2 increased DASH score compared with Strategy 1^b^. The second hypothesis was tested only if *P* < 0.05 was met for the first hypothesis. No other adjustments for multiple comparisons were made.

Individualized nutrition education, enhanced with online shopping technologies and training (Strategy 2), significantly increased DASH score by 3.8 (95% CI (0.8, 6.9), *P* = 0.01) compared with education without online enhancement (Strategy 1), from baseline to 3 months (Table 3). This increase was at least partially due to an increase in daily mean servings of fruits in Strategy 2 versus 1 (*P* < 0.05) (Table 4). From baseline to 6 months, Strategy 2 resulted in no difference in DASH score (1.8, 95% CI (− 1.9, 5.5), *P* = 0.34) compared with Strategy 1 (Table 3).

**Table 4:** Change in DASH score components at 3 months

	Control (*n* = 46) mean (95% CI)	Strategy 1 (*n* = 100) mean (95% CI)	Strategy 2 (*n* = 101) mean (95% CI)	Strategies 1 and 2 versus control mean (95% CI)	*P* value	Strategy 2 versus 1 mean (95% CI)	*P* value
**Fruit servings, 1,000** **kcal** **d**^**–1**^							
At baseline	1.8 (1.3, 2.3)	1.6 (1.3, 2.0)	1.3 (1.0, 1.7)				
Change (baseline to 3 months)	0.2 (−0.3, 0.7)	0.7 (0.4, 1.0)	1.2 (0.9, 1.5)	0.8 (0.2, 1.3)	<0.01	0.5 (0, 0.9)	0.03
**Vegetable servings, 1,000** **kcal** **d**^**–1**^							
At baseline	3.3 (2.7, 3.8)	2.7 (2.3, 3.1)	2.7 (2.3, 3.1)				
Change (baseline to 3 months)	0.3 (−0.3, 0.9)	0.8 (0.4, 1.2)	0.8 (0.4, 1.2)	0.5 (−0.2, 1.2)	0.18	0 (−0.6, 0.6)	1.00
**Total dairy servings, 1,000** **kcal** **d**^**–1**^							
At baseline	1.7 (1.4, 2.0)	1.7 (1.5, 1.9)	1.6 (1.4, 1.8)				
Change (baseline to 3 months)	−0.3 (−0.7, 0.1)	−0.2 (−0.4, 0.1)	0 (−0.2, 0.2)	0.2 (−0.2, 0.7)	0.27	0.2 (−0.2, 0.5)	0.32
**Low-fat dairy servings, 1,000** **kcal** **d**^**–1**^							
At baseline	0.3 (0.2, 0.5)	0.3 (0.2, 0.4)	0.3 (0.1, 0.4)				
Change (baseline to 3 months)	0 (−0.2, 0.2)	0.3 (0.2, 0.4)	0.2 (0.1, 0.3)	0.3 (0.1, 0.4)	<0.01	−0.1 (−0.2, 0.1)	0.43
**Total grain servings, 1,000** **kcal** **d**^**–1**^							
At baseline	5.5 (4.8, 6.2)	5.4 (4.9, 5.9)	5.0 (4.5, 5.5)				
Change (baseline to 3 months)	−1.3 (−2.0, −0.7)	−1.0 (−1.4, −0.5)	−0.5 (−1.0, −0.1)	0.6 (−0.2, 1.4)	0.14	0.5 (−0.2, 1.1)	0.16
**Whole grain servings, 1,000** **kcal** **d**^**–1**^							
At baseline	1.6 (1.2, 2.0)	1.3 (1.0, 1.6)	1.2 (0.9, 1.5)				
Change (baseline to 3 months)	0 (−0.4, 0.4)	0.7 (0.4, 1.0)	1.0 (0.7, 1.3)	0.9 (0.4, 1.4)	<0.01	0.3 (−0.1, 0.7)	0.13
**Meat servings, 1,000** **kcal** **d**^**–1**^							
At baseline	5.0 (4.2, 5.7)	5.1 (4.6, 5.6)	5.5 (4.9, 6.0)				
Change (baseline to 3 months)	−0.2 (−1.0, 0.6)	−0.4 (−0.9, 0.1)	−0.6 (−1.1, −0.1)	−0.3 (−1.2, 0.6)	0.52	−0.2 (−0.9, 0.5)	0.52
**Nuts/seeds servings, 1,000** **kcal** **d**^**–1**^							
At baseline	0.9 (0.5, 1.4)	1.1 (0.8, 1.4)	1.1 (0.8, 1.4)				
Change (baseline to 3 months)	0.5 (0, 1.0)	0.1 (−0.2, 0.4)	0.4 (0, 0.7)	−0.3 (−0.9, 0.3)	0.36	0.3 (−0.2, 0.7)	0.24
**Sweets servings, 1,000** **kcal** **d**^**–1**^							
At baseline	2.5 (1.9, 3.0)	2.3 (1.9, 2.7)	2.5 (2.1, 2.8)				
Change (baseline to 3 months)	−1.1 (−1.7, −0.5)	−0.6 (−1.0, −0.2)	−0.9 (−1.3, −0.5)	0.4 (−0.3, 1.0)	0.29	−0.3 (−0.8, 0.3)	0.33
**Fats/oils servings, 1,000** **kcal** **d**^**–1**^							
At baseline	4.0 (3.4, 4.5)	3.6 (3.1, 4.0)	3.7 (3.3, 4.1)				
Change (baseline to 3 months)	−0.8 (−1.4, −0.2)	−0.6 (−1.0, −0.2)	−1.0 (−1.4, −0.6)	0 (−0.7, 0.7)	0.98	−0.4 (−1.0, 0.2)	0.14
**Sodium, mg** **d**^**–1**^							
At baseline	2.836.4 (2.580.5, 3,092.4)	2,773.5 (2,585.9, 2,961.1)	2,633.8 (2,447.0, 2,820.6)				
Change (baseline to 3 months)	−615.0 (−885.5, −344.5)	−609.4 (−787.5, −431.3)	−546.9 (−721.5, −372.3)	36.9 (−272.5, 346.2)	0.81	62.5 (−183.4, 308.4)	0.62

### Prespecified secondary endpoints

From baseline to 3 months, SBP, diastolic blood pressure (DBP) and body mass index (BMI) did not decrease in the control group (Supplementary Table 14). From baseline to 3 months, SBP decreased in Strategy 1 and Strategy 2 groups by −6.6 (−9.8, −3.4) and −5.7 (−8.7, −2.8) mmHg, respectively. DBP decreased in Strategy 1 and Strategy 2 groups by −2.4 (−4.2, −0.6) and −2.0 (−3.9, −0.1) mmHg, respectively. BMI decreased in Strategy 1 and Strategy 2 groups by −0.4 (−0.7, −0.2) and −0.8 (−1.0, −0.5) kg m^–2^, respectively. In between-group comparisons, however, no differences were found.

From baseline to 6 months, SBP decreased in control, Strategy 1 and Strategy 2 groups by −5.4 (−10.7, −0.1), −5.2 (−8.8, −1.6) and −4.2 (−7.8, −0.5) mmHg, respectively (Supplementary Table 15). DBP decreased in control and Strategy 1 groups by −3.7 (−7.1, −0.3) and −4.4 (−6.7, −2.2) mmHg, respectively, but did not decrease in the Strategy 2 group. BMI decreased in Strategy 1 and Strategy 2 groups by −0.6 (−0.9, −0.3) and −0.7 (−1.1, −0.4) kg m^–2^, respectively. In between-group comparisons, however, no differences were found.

From baseline to either 3 months (Supplementary Table 16) or 6 months (Supplementary Table 17), non-HDL-C, total cholesterol and triglycerides (TG) were not reduced by the combined Strategies 1 and 2 versus the enhanced control.

### COVID impact analyses

In the prepandemic cohort, from baseline to 3 months, the combined Strategies 1 and 2 increased the DASH score by 8.3 (95% CI (3.4, 13.3), *P* = 0.001) compared with the enhanced control (Supplementary Table 18). From baseline to 6 months, the combined Strategies 1 and 2 did not result in a significant difference in DASH score: 5.1 (95% CI (−0.8, 11.1), *P* = 0.09) compared with control (Supplementary Table 19).

Strategy 2 resulted in no significant difference in DASH score, at 3.1 (95% CI (−1.3, 7.6), *P* = 0.017), from baseline to 3 months compared with Strategy 1 (Supplementary Table 18). From baseline to 6 months, the increase in DASH score was nonsignificant at 1.2 (95% CI (−4.2, 6.6), *P* = 0.67) when comparing Strategy 2 with Strategy 1 (Supplementary Table 19).

From baseline to 3 months, there were no reductions in SBP, DBP, BMI, non-HDL-C, total cholesterol or TG comparing the combined Strategies 1 and 2 versus the enhanced control (Supplementary Table 18).

### Subgroup analysis

A prespecified exploratory subgroup analysis evaluating absolute mean DASH score difference for selected subgroups within Strategies 1 and 2 versus control was performed. Greater improvements in DASH diet adherence were associated with older age (51–75 versus 21–50 years), white race (white versus nonwhite) and baseline hypertension (with versus without) (Supplementary Table 20; interaction terms, *P* ≤ 0.01). There was no difference by gender (men versus women).

## Discussion

In SuperWIN, all three study groups demonstrated increases in adherence to the DASH dietary pattern from baseline to 3 months, along with persistence of increased DASH adherence at 6 months. On top of a data-enhanced medical nutrition therapy session, the addition of an individualized, food-purchasing, data-guided, ‘teaching in the aisles’ nutrition intervention increased DASH adherence. Introduction to online shopping and technologies to improve purchases, meal planning and recipe quality further increased DASH adherence. Participant engagement before the pandemic was remarkably high for a community-based study. This was reflected by extremely high visit attendance and near-perfect dietary intake and biometrics collection. Even during the disruption of the pandemic^6,7^, visit attendance and data collection remained comparable to prepandemic community-based studies^8^.

Globally, suboptimal diet is accountable for more deaths than any other risk factor across age, sex and socioeconomic status^9^. While the scores used to measure DASH adherence have varied across studies, the association between DASH scores and clinical outcomes has been consistently demonstrated^10^. As an example, a recent meta-analysis found a linear dose–response for each five-point increase in DASH score and associations with lower all-cause, cardiovascular (CV), stroke and cancer mortality of 5, 4, 3 and 3%, respectively^11^. In both the PREDIMED trial and Western and non-Western observational studies, it has been shown that even small measures of increased diet quality result in large CV risk reductions over time^12–17^. While the validation studies of DASH were performed in a highly controlled environment (that is, feeding studies), the PREMIER trial, which enrolled free-living participants, demonstrated the impact on DASH adherence through an intensive 18-visit, 6-month program conducted at academic centers^18,19^. The 2021 update to the 2006 American Heart Association (AHA) guidelines continues to recommend evidence-based, heart-healthy dietary patterns, including DASH^20^.

Despite longstanding recommendations, adoption of DASH across the United States^21^ and many other countries^1^ remains low. From 2007 to 2012, using a different DASH adherence score and the National Health and Nutrition Examination Survey, it was estimated that, in the United States, individuals with hypertension scored only about 2.6–2.7 from a total score of 9 (ref. ^22^). A 2019 AHA Science Advisory concluded that ‘immediate action is needed’ to innovate new approaches to close this gap^23^. Specific recommendations included creation of new partnerships (including with retailers) for sponsored research. The need for research on the health benefits of online shopping and smart technologies (for example, nutrition and health applications) was also highlighted. We incorporated several such approaches through a supermarket-based partnership. SuperWIN may now be able to extend the findings of previous dietary trials through an innovative, dietitian-driven, grocery store-based model, with technologies that facilitate broad convenience and accessibility.

In SuperWIN, the interventions increased DASH score by a clinically meaningful 4.7 points from baseline to 3 months compared with the control group. The control group experienced a 5.8-point increase in DASH score, which is larger than the change in DASH (or DASH food group) adherence for standard-of-care interventions reported in other recent clinical trials^24,25^. It is likely that the standard of care delivered in SuperWIN had a more favorable impact on DASH adherence due to the use of the participants’ preferred stores, dietitians’ expertise and provision of DASH-focused dietary intake data. At 6 months, while DASH scores decreased slightly, they remained significantly increased from baseline by 4.4 (0.6, 8.1), 6.6 (4.0, 9.2) and 8.4 (5.9, 11.0) in the control, Strategy 1 and Strategy 2 groups, respectively. Additional research may identify opportunities to increase not only the initial post-intervention dietary improvements, but also to maximize their persistence over long-term follow-up. Opportunities include: (1) addition of more in-person visits, (2) use of telenutrition visits and (3) continued delivery of updated purchasing behavior data to the participant and/or dietitian.

The rapid yearly increase in online shopping across all age groups in the United States^26^ may provide unique opportunities to address barriers to shopping and making better food choices. In addition, utilization of mobile health-focused applications in patients with CV risk factors is increasing^27^. In SuperWIN, the addition of online technologies increased DASH score by a clinically meaningful 3.8 points from baseline compared with education that did not incorporate them. Considering that SuperWIN enrolled a ‘late tech-adopters’ population, it is possible that early tech-adopters may experience even greater dietary improvements. As online shopping becomes even more common in the United States and other countries, retail platforms will offer increasingly comprehensive features and services at lower prices. Collectively, this may offer opportunities to mitigate challenges related to poor health literacy, busy schedules, inadequate personal or public transportation, distance to the nearest grocery retailer, disability and industry marketing^28^. In 2019, the Supplemental Nutrition Assistance Program launched its pilot program to understand whether online shopping might increase the purchase of healthy foods^29,30^.

In the overall cohort, secondary endpoints of blood pressure, lipids and BMI were not improved by the interventions compared with the enhanced control. However, as an example, SBP decreased by −6.6 and −5.7 mmHg at 3 months in the Strategy 1 and Strategy 2 arms, respectively, while SBP remained decreased by −5.2 and −4.2 mmHg at 6 months in the Strategy 1 and Strategy 2 arms, respectively. A similar pattern was found for BMI. More research will be needed to quantify the impact of these interventions on secondary outcomes.

Our trial has certain limitations. Our cohort was receiving routine primary care and was predominantly middle-aged, female, married or living with a partner and living in households with a reasonable total annual household income (for comparison, in 2020, median US household annual income was US$69,560 (ref. ^31^)). The COVID-19 pandemic dramatically not only impacted clinical trials but also shopping behavior and many other aspects of our participants’ lives (for example, work, childcare). In terms of evaluation of SBP, DBP and lipid changes, we did not collect data on medication doses at any time point. In addition, medication use was assessed by a survey rather than by a rigorous evaluation by the study dietitian. We also did not collect medication use at 3 months. We performed a limited number of BP measurements at each time point, which may have curtailed precision. Collectively these limitations, as well as the baseline levels in this cohort, may have reduced our ability to more rigorously assess changes in these secondary endpoints. In future, additional components (for example, exercise interventions, detailed medication assessments, medication titration or medication adherence counseling through the retail pharmacy) may be combined with ‘SuperWIN-like’ dietary interventions to target these outcomes.

In conclusion, all three study groups had higher DASH adherence at 3 months, which persisted until the final study assessments at 6 months. Individualized, in-person, dietitian-led nutrition education focusing on the DASH dietary pattern, guided by electronic food-purchasing data and delivered within each participant’s home supermarket, increased DASH adherence. The addition of technologies for online shopping with both grocery pick-up and home delivery options, as well as food comparisons and meal preparation, increased DASH adherence. More research will be needed to better understand the effects of these interventions on downstream secondary outcomes. These findings demonstrate the importance of sponsored research with the retail industry, the opportunities to enhance dietary quality through grocery stores and retail clinics, as well as the efficacy of specific types of new interventions.

## Methods

SuperWIN was a randomized, parallel-assignment, active-control, efficacy trial. For the protocol and statistical analysis plan, refer to Supplementary Notes 1 and 2. The study was registered on Clinicaltrials.gov (NCT03895580). The University of Cincinnati (UC) Institutional Review Board approved the protocol, and all participants gave written informed consent. All participant visits were conducted across 13 Kroger supermarket locations in Ohio and Kentucky, each of which had a clinic that allowed for study visits and assessments. The authors vouch for the accuracy of the data, as well as for the fidelity of this report to the trial protocol. A detailed description of the methods of this trial has been published^10^.

### Participants

All participants were required to have a primary care clinician at UC Health. Lists of UC Health patients likely to meet the eligibility criteria (for example, diagnosis code of hypertension) were generated from UC Health’s Clarity Database (Epic Systems Corp.). The UC study coordinator then mailed study materials to those patients living near the study stores. Phone calls, texting, emails and flyers were also used. Interested patients were phone-screened by the coordinator and, if eligible, were entered into the run-in period. Men and women aged between 21 and 75 years were eligible if they were the primary food planner for their household, were an existing shopper at one of the study Kroger supermarkets, were able to shop and prepare food independently and had a home computer (Supplementary Table 4). Participants were enrolled if they had at least one CV risk factor: (1) SBP >130 mmHg, DBP >80 mmHg and/or treatment with an antihypertensive medication; (2) obesity, defined as BMI ≥30kg m^–2^; and/or (3) non-HDL-C ≥130 mg dl^–1^ and/or treatment with a lipid-lowering medication. Key exclusion criteria included current treatment with another dietary or weight loss intervention, use of Kroger’s online shopping platform within 12 months, previous use of Kroger’s dietary counseling services, baseline SBP ≥190 mmHg, DBP ≥110 mmHg or non-HDL-C ≥190 mg dl^–1^. All participants provided verbal consent to enter the run-in period and written informed consent at the beginning of the first study visit. Participants were eligible to receive up to two incentives of US$25 throughout the trial.

### Randomization and blinding

Following a run-in period consisting of collection of baseline dietary intake via phone, and survey information via email, participants attended a study visit at their assigned store location. All study visits were conducted in the store by a supermarket registered dietitian (‘study dietitian’) (Supplementary Table 5). At the end of visit, once eligibility and interest were confirmed, participants were randomized in a 2:2:1 ratio to: (1) individualized, in-store nutrition education (Strategy 1); (2) individualized, in-store nutrition education enhanced with online technologies and training (Strategy 2); or (3) no further education (control). Randomization was accomplished by the study dietitian using the preloaded, stratified randomization list in the Research Electronic Data Capture (REDCap) randomization module, which programmatically displayed the assignment. Randomization was stratified by characteristics reported to influence food choice: age (two levels), gender (two levels) and household size (three levels). The block size used was five.

The principal investigators and other key study staff at UC and Cincinnati Children’s Hospital and Medical Center (CCHMC) were not blinded; each could access REDCap and determine participant group assignment. Raw dietary intake data were not stored in REDCap. All calculations of DASH score during follow-up and changes in DASH score were performed only after completion of the study.

### Procedures

All participants received a 30-min medical nutrition therapy session (standard of care) at the first study visit, before randomization. The dietitian educated participants on the evidence-based DASH diet in relation to CV risk factor reduction, set personal DASH dietary goals based on current intake and developed an action plan to meet those goals. Compared with typical practice, this session was enhanced by displaying each participant’s baseline dietary intake data (collected during the run-in period) via simple figures and tables. These highlighted the participant’s mean servings of each DASH food group, the mean serving goals per food group, the specific foods consumed, as well as the time and location of consumption. DASH food serving goals were established based on a caloric goal for either weight maintenance or loss.

Participants randomized to Strategies 1 and 2 were scheduled for six additional in-store educational sessions performed at 2-week intervals over the next 3 months. Educational visits in Strategies 1 and 2 utilized the physical supermarket environment (‘teaching within the aisles’). Strategy 2 participants were also trained by the study dietitians in a stepwise manner on the store’s online shopping platform, free home delivery services and two other healthcare applications (Supplementary Table 6). Nutrition education themes, learning outcomes and skill-building exercises were consistent across Strategies 1 and 2. Education in Strategies 1 and 2 was guided by dietitian and participant review of each participant’s updated, individualized, Kroger purchasing data (automatically collected via a store loyalty card) at the beginning of each session. These data, displayed via simple figures and tables, highlighted the purchases of food groups and specific food items, as well as counts of purchases and money spent since randomization.

All randomized participants were scheduled for in-store study assessments (interviews and biometric measurements) at baseline and at 3 and 6 months from randomization. Raw dietary intake data were collected by phone at baseline and at 3 and 6 months. Medication intake was collected via an emailed survey at baseline and at 6 months.

### Endpoints

The primary endpoint was change in DASH score, which was calculated on a 0–90-point scale^24^ with a higher score indicating greater adherence to a DASH diet (Supplementary Table 7). In this trial, DASH score was calculated from 11 component scores based on the alignment between actual intake and the DASH serving recommendations (for example, whole grains, vegetables, fruits).

At baseline and at 3 and 6 months, three 24-dietary phone recalls (two weekday and one weekend) were collected at each time point by trained dietary interviewers from the Bionutrition Center at CCHMC. Only after completion of the study were these raw dietary intake data used to calculate DASH scores. DASH scores were first calculated for individual recalls followed by calculation of a participant’s mean DASH score at each time point. Use of dietary intake recalls, collection on multiple days and a comprehensive scoring system to reflect the multiple components of DASH were used to optimize measurement of DASH dietary pattern adherence.

Prespecified secondary endpoints included SBP, DBP, BMI, non-HDL-C, total cholesterol and TG measured during study visits by the study dietitians at baseline and at 3 and 6 months. COVID-19 impact analyses were prespecified before database lock. The prepandemic subgroup was defined as those participants who were randomized and had completed 3 months of follow-up before study cessation, when the United States declared a national emergency on 13 March 2020.

### Statistical analyses

We evaluated change in DASH score within each group from baseline to 3 months and from baseline to 6 months. We formally tested two hypotheses in hierarchical fashion: (1) whether, from baseline to 3 months, Strategies 1 and 2 combined increased DASH score compared with an enhanced control; and (2) whether, from baseline to 3 months, Strategy 2 increased DASH score compared with Strategy 1 (see above). The second hypothesis was tested only if *P* < 0.05 was met for the first hypothesis, which preserved an experiment-wise error rate of 0.05. We used an intention-to-treat analysis to test both hypotheses. We used regression-based, multiple imputation techniques for missing follow-up outcome data. The primary comparisons used a mixed model for repeated measures, controlling for stratification and other previously selected baseline covariates (see legends in tables for the specific covariates used for each comparison), with DASH score as the dependent variable and treatment group, time and group × time interaction as independent variables. Residual plots were used to confirm that model assumptions had been met. We estimated that 100 participants in each intervention group (total, 200) and 50 participants for the control group would be required to provide 93 and 97% power to detect a five-point difference in change from baseline to 3 months in the DASH score for the first and second hypothesis tests, respectively. Power estimates were performed at two-sided alpha = 0.05 and assumed equal s.d. = 9 for the primary endpoint^32^. Although not powered to test subgroup treatment interactions, a separate analysis of variance model was used for each subgroup to compare Strategies 1 and 2 combined with control on DASH score at 3 months. Subgroups were selected using baseline characteristics (for example, male versus female) that might be associated with the efficacy of the interventions. Beyond the two formal hypotheses tested in this study, no corrections for multiple comparisons were performed. All analyses were performed using SAS 9.4 TS1M5.

### Reporting summary

Further information on research design is available in the Nature Portfolio Reporting Summary linked to this article.

## Online content

Any methods, additional references, Nature Portfolio reporting summaries, source data, extended data, supplementary information, acknowledgements, peer review information; details of author contributions and competing interests; and statements of data and code availability are available at 10.1038/s41591-022-02077-7.

## Supplementary information

Supplementary Information Supplementary Tables 1–21, Notes 1 (protocol) and 2 (statistical analysis plan).

Reporting Summary

## Data Availability

The datasets generated during and/or analyzed during the current study are not publicly available, but may be made available upon reasonable request to the corresponding author.
