# Supplementary material for: Efficacy of supermarket and web-based interventions for improving dietary quality: a randomized, controlled trial
Source: Nat Med. 2022 Dec 1;28(12):2530–6. doi: 10.1038/s41591-022-02077-7 (PMC9800276; doi:10.1038/s41591-022-02077-7)
Supplement: Supplementary file 1 — Supplementary Tables 1–21, Notes 1 (protocol) and 2 (statistical analysis plan). [file 41591_2022_2077_MOESM1_ESM.pdf]

nature medicine

Article

https://doi.org/10.1038/s41591-022-02077-7

Efficacy of supermarket and web-based interventions for improving dietary quality: a randomized, controlled trial

In the format provided by the authors and unedited

SUPPLEMENTARY APPENDIX

Table of Contents

Table S1: SuperWIN Investigators	2
Table S2: Study Dietitians and Coordinators	3
Table S3: Protocol Amendments	4
Table S4: Eligibility Criteria for the SuperWIN Trial	5
Table S5: SuperWIN Tasks and Assessments	8
Table S6: Components of Online Enhancement in Strategy 2	9
Table S7: Components of the Adapted DASH Score Used in the SuperWIN Trial	10
Table S8: Strategy 1 and 2 Visit Completion Frequency Before and During the COVID-19 Pandemic	11
Table S9: Dietary Intake and Biometrics Data Collection Before and During the COVID-19 Pandemic	12
Table S10: DASH Score Components at Baseline and 6 Months	13
Table S11: Other Nutritional Measurements at Baseline and 3 Months	15
Table S12: Other Nutritional Measurements at Baseline and 6 Months	16
Table S13: Medication Treatment at Baseline and 6 Months	17
Table S14: Systolic Blood Pressure, Diastolic Blood Pressure, and Body-Mass Index at Baseline and 3 Months	18
Table S15: Systolic Blood Pressure, Diastolic Blood Pressure, and Body-Mass Index at Baseline and 6 Months	19
Table S16: Non-HDL Cholesterol, Total Cholesterol, and Triglycerides at Baseline and 3 Months	20
Table S17: Non-HDL Cholesterol, Total Cholesterol, and Triglycerides at Baseline and 6 Months	21
Table S18: Pre-COVID-19 Subgroup Analyses at Baseline and 3 Months	22
Table S19: Pre-COVID-19 Subgroup Analyses at Baseline and 6 Months	24
Table S20: Change in DASH score for Subgroups at 3 Months	26
Table S21: Study Supermarkets and Locations Used	28
Supplementary Note 1: Study Protocol	29
Supplementary Note 2: Statistical Analysis Plan	59

Table S1: SuperWIN Investigators

Dylan L. Steen, M.D., M.S.	University of Cincinnati	Co-PI
Sarah C. Couch, Ph.D., R.D.	University of Cincinnati	Co-PI
Mark H. Eckman, M.D., M.S.	University of Cincinnati	Sub-I
Deepak L. Bhatt, M.D., M.P.H.	Brigham & Women’s Hospital and Harvard Medical School	Sub-I
Eileen C. King, Ph.D.	Cincinnati Children’s Hospital and Medical Center	Sub-I
Brian E. Saelens, Ph.D.	University of Washington and Seattle Children’s Research Institute	Sub-I

Table S2: Study Dietitians and Coordinators

Kroger Registered Dietitians (RD)	Sarah Limbert, Amelia Noel, Madison Linek, Tiffany Naticchioni, Victoria LeMaire, Emily Rider, Elizabeth Calvelage, Laura Brown, and Ani Mueller.
UC Coordinators	Robert Helsley and Tina Ramey.
Kroger RDs served as the study dietitians. In addition to dietary education, they conducted all in-store study visit procedures.	

Table S3: Protocol Amendments

Amendment Number	Date	Components Amended
**1**	February 12, 2019	Minor clarifications on timing of screening and run-in tasks. For example: Screening phone call will be conducted ≥18 days from Day 0 as opposed to 18 days from Day 0.
**2**	April 18, 2019	Corrected protocol to state DASH was a 90-point score, not an 80-point score.<br>Clarified recruitment methods to include letters, postcards, and phone calls, as well as printed materials in UC Health primary care offices and Kroger study stores.<br>Minor eligibility criteria revisions:<br>Inclusion criterion 9b: Changed: “Ability to stand for significant periods of time to grocery shop and prepare food at home” to “Any mobility issues that do not impact grocery shopping frequency or preparation of food at home”.<br>Exclusion criterion 12: Changed: “Previous use of the Kroger’s online shopping platform (Kroger.com or the Kroger app)” to “Frequent (≥1x/within the last 12 months) shopper using Kroger’s online shopping platform (Kroger.com or the Kroger app) or unwillingness to use it throughout the study”.
**3**	October 8, 2019	Protocol amended to include option to re-contact participants for up to 2 years after authorization.
**4**	January 24, 2020	Added EPIC’s MyChart, emailing, and texting to recruitment methods.
**5**	February 24, 2020	Minor eligibility revision:<br>Inclusion criterion 5: Changed: “Existing shopper at a study site Kroger (≥50% of grocery store food dollars spent at selected study site Kroger) with a Kroger Loyalty ID number” to “Regular existing shopper at a study site Kroger (≥50% of grocery store food dollars spent at Kroger) with a Kroger Loyalty ID number.”
**6**	June 16, 2021	Minor changes regarding collected purchasing data:<br>Changed: “In addition, household purchase data beginning 365 days prior to Day 0 will be pulled into the study database” to “In addition, household purchase data will be pulled into the study database by 84.51°, using patient identifiable information (e.g. name, address) submitted via password-protected communications, from 365 days prior to Day 0.”<br>Changed: “The CCHMC Data Management and Analysis Center (DMAC) staff will ensure the safe transfer and storage of participant data (including from 84.51°)” to “The CCHMC Data Management and Analysis Center (DMAC) staff will ensure the safe transfer and storage of participant data (including to and from 84.51°).”

Table S4: Eligibility Criteria for the SuperWIN Trial

INCLUSION CRITERIA	
1.	Age ≥21 to ≤75 years on the start date of the study (Day 0).
2.	Speak, write, and read English fluently.
3.	In-person outpatient visit with a UC Health primary care network (PCN) provider within the last 12 months.
4.	Major food planner of their household, which may include planning, purchasing, and/or preparing greater than 50% of the meals or foods consumed at home.
5.	Regular existing shopper at a study site Kroger (≥50% of grocery store food dollars spent at Kroger) with a Kroger Loyalty ID number.
6.	Willing to use a new individual Kroger Loyalty ID number for the duration of the study.
7.	Access to a home desktop or laptop computer with reliable internet access.
8.	Access to an email account that can be used for the duration of the study.
9.	Able to independently purchase and prepare food:<br>a. Reliable transportation to participating Kroger store<br>b. Any mobility issues that do not impact grocery shopping frequency or preparation of food at home<br>c. Tools and equipment needed to prepare own food at home, such as a kitchen equipped with basic cooking tools (e.g. burner, pan, spatula, sink)
10.	Presence of a cardiovascular risk factor(s) (at least one):<br>a. Hypertension (defined by one of the following):<br>i. Blood pressure (BP) measurement: systolic BP 130–189 mm Hg and/or diastolic BP 80-109 mm Hg (at Visit 1).<br>ii. Current treatment with an oral medication prescribed by a physician for blood pressure lowering (defined by one of the following):<br>1. Beta-blockers<br>2. Diuretics (e.g. thiazide or thiazide-like diuretics, loop diuretics, aldosterone antagonists, potassium-sparing diuretics)<br>3. Angiotensin-converting enzyme inhibitors<br>4. Angiotensin II receptor blockers<br>5. Direct renin inhibitors (i.e. aliskiren)<br>6. Calcium channel blockers (dihydropyridine and nondihydropyridine)

	7. Calcium alpha$_2$-agonist and other centrally acting drugs (e.g. clonidine)<br>8. Direct vasodilators (e.g. hydralazine)<br>b. Body-mass index (BMI) measurement ≥30 kg/m^2 (at Visit 1).<br>c. Hypercholesterolemia (defined by one of the following):<br>i. Low-density lipoprotein cholesterol (LDL-C)* 130-189 mg/dl (at Visit 1)<br>ii. Current treatment with a medication prescribed by a physician for hypercholesterolemia (defined by one of the following):<br>1. Statins<br>2. Proprotein convertase subtilisin/kexin type 9 (PCSK9) inhibitors<br>3. Absorption inhibitors (i.e. ezetimibe)<br>4. Fibrates<br>5. High-dose niacin (≥1 g per day)<br>6. Bile acid sequestrants (e.g. cholestyramine)
**EXCLUSION CRITERIA**	
1.	Unwillingness or inability to modify current diet.
2.	Actively engaged in another dietary intervention or taking a weight-loss supplement.
3.	SBP ≥190 mm Hg or DBP ≥110 mm Hg (at Visit #1).
4.	Low-density lipoprotein cholesterol (LDL-C)* ≥190 mg/dl, triglycerides (TG)^ ≥600 mg/dl, or glucose ≥400 mg/dl (at Visit 1).
5.	Evidence of prior diagnosis of severe chronic kidney disease (CKD) defined by electronic health record codes for dialysis, CKD Stage 4 or 5 and/or last estimated glomerular filtration rate (eGFR) ≤30 ml/min/1.73^2 (based on Modification of Diet in Renal Disease [MDRD] formula).
6.	Active cancer other than non-melanoma skin cancers (i.e. basal cell carcinoma or squamous cell carcinoma).
7.	Diagnosis of celiac disease, ulcerative colitis, or Crohn's disease.
8.	High risk for alcohol use disorder:<br>a. ≥21 drinks a week (men)<br>b. ≥14 drinks a week (women)
9.	Women who are pregnant or plan to become pregnant within the next 6 months from the start of the study (Day 0).
10.	Food allergies requiring a specialized diet, including allergies to dairy products, eggs, peanuts, tree nuts, soy, gluten-containing wheat and grains, fish, and shellfish.

11.	Previous visit at TLC for diet counseling.
12.	Frequent (≥1x/within the last 12 months) shopper using Kroger's online shopping platform (Kroger.com or the Kroger app) or unwillingness to use it throughout the study.
13.	Participant lives greater than 20 miles from any participating Kroger study store.
14	Women, Infants, and Children (WIC) beneficiaries in their household.

Addendum: *In REDCap, LDL-C was to be automatically calculated using the Friedewald equation after the dietitians had entered values for total cholesterol, high-density lipoprotein cholesterol (HDL-C), and triglycerides (TG). These values were obtained using the Cardiocheck Plus analyzer (PTS Diagnostics, Whitestown, IN) on a fingerstick blood sample at Kroger's The Little Clinic (in-store retail clinic). Upon completion of the trial, it was noticed that the calculation programmed into REDCap was for non-HDL-C, hence eligibility was determined using non-HDL-C. Please note that SuperWIN participants were not required to fast. In addition, the majority had obesity, so non-HDL-C might be considered a preferrable target (compared to LDL-C). ^The measurement cutoff for TG on the Cardiocheck Plus analyser was 500 mg/dl.

Table S5: SuperWIN Tasks and Assessments

					Assessments	
**Method/Location**	**Study Team Tasks**	**Before Visit 1**	**Visit 1 (Day 0)**	**Intervention Visits**	**3-mon**	**6-mon**
Phone	Obtain Verbal Consent	X				
Phone	Screen for Eligibility	X				
Phone	Schedule Visit 1 (if phone screen suggested eligibility)	X				
Email/Internet	Send Demographics Survey	X				
Email/Internet	Send Medical History Survey	X				X
Email/Internet	Send Food-related Self-Efficacy and Eating Behavior Survey	X			X	
Phone	Collect Dietary Intake Recalls[1]	X			X	X
In-store	Measure Height		X			
In-store	Measure Weight		X		X	X
In-store	Measure Waist and Hip Circumferences		X		X	X
In-store	Measure Blood Pressure		X		X	X
In-store	Measure Blood Lipid Panel[2,3]		X		X	X
In-store	Measure Blood Glucose[3]		X		X	X
In-store	Deliver Medical Nutrition Therapy		X			
In-store	Randomization (if appropriate)		X			
In-store	Assign New Kroger Plus Card for Individual Use During Study		X			
Email/Internet	Send SuperWIN Intervention Survey				X	
In-store	Deliver Education Modules[4]			X		
In-store	Review Updated Purchasing Data Snapshot with Participant[4]			X		
In-store	Complete Checklist[4]			X		
Email/Internet	Send Videos to Participant for Review[5]			X		

Strategy 1: In-store education only. Strategy 2: In-store education plus online training and tools.

[1]Diet recall performed 3-times (2 on weekdays, 1 on weekend) at each timepoint (i.e. baseline, 3 months, 6 months). [2]Blood lipid panel consisted of total cholesterol, non-high-density lipoprotein cholesterol (non-HDL-C), and triglycerides. [3]Participants were not required to fast. [4]Applicable to participants randomized to either Strategy 1 or Strategy 2 group. [5]Videos reviewed online enhancements (e.g. how to shop online). Applicable only to participants in Strategy 2.

Table S6: Components of Online Enhancement in Strategy 2

	**Description**
Kroger.com (and/or Kroger's mobile shopping application)	Kroger's proprietary online shopping site. If participants preferred to online shop using a mobile application, training on Kroger's mobile application was emphasized. During most of the trial, placing an online order by either method was $4.95 per shopping trip.
The Grocery Runners (GR)	A same day "last-mile" delivery service. Participants who online shopped at Kroger had unlimited access to free home delivery for the duration of their participation in the study. Participants could elect to not use home delivery, instead picking up their online orders at the store themselves.
OptUP	Kroger's proprietary digital nutrition rating tool to simplify healthier shopping. There was no cost associated with using this tool.
Yummly	A proprietary platform to assist with meal-planning and recipe-building. There was no cost associated with using this tool.
Participants were oriented to Kroger.com and GR's home delivery service at Visit 1 after being randomized to Strategy 2. OptUP and Yummly were introduced at subsequent visits.	

Table S7: Components of the Adapted DASH Score Used in the SuperWIN Trial

DASH Score component	Maximum DASH Score	Standard for Maximum Score	Standard for Minimum Score of 0
Fruits	10	≥2.25 servings/1000 calories per day	0 servings/1000 calories per day
Vegetables	10	≥2.25 servings/1000 calories per day	0 servings/1000 calories per day
Total Grains	5	≥3.5 servings/1000 calories per day	0 servings/1000 calories per day
Whole and Semi-whole Grains	5	≥1.75 servings/1000 calories per day	0 servings/1000 calories per day
Meats, Poultry, and Fish	10	≤3.0 servings/1000 calories per day	≥6.0 servings/1000 calories per day
Total Dairy	5	≥1.5 servings/1000 calories per day	0 servings/1000 calories per day
Fat-free and Low-fat Dairy	5	≥1.25 servings/1000 calories per day	0 servings/1000 calories per day
Nuts, Seeds and Legumes	10	≥0.28 servings/1000 calories per day	0 servings/1000 calories per day
Fats and Oils	10	≤1.25 servings/1000 calories per day	≥2.5 servings/1000 calories per day
Sweets and Added Sugars	10	≤0.35 servings/1000 calories per day	≥0.7 servings/1000 calories per day
Sodium	10	≤2300 mg per day	≥3300 mg per day

DASH = Dietary Approaches to Stop Hypertension. DASH score range = 0-90 calculated from the sum of all DASH score components.

Maximum component scores could be achieved when the intake met the DASH food group recommendation**, whereas lower intakes were scored proportionately. If lower intakes were favored by the DASH good group recommendations, reverse scoring was applied (i.e. meats, fats, sweets, sodium were reverse scored).

A minimum score of 0 was achieved when no servings were eaten of DASH recommended food groups (i.e. fruits, vegetables, grains, dairy, nuts) or twice the recommended servings were eaten of food groups for which advice was to limit consumption (i.e. meats, fats, sweets, sodium). DASH goals were evaluated based on actual calories consumed rather than calorie goals established by the study dietitian, in order to evaluate the quality of the participants' diets based on actual energy intake.

**DASH Food Group Recommendations based on a 2000 calorie/day DASH eating plan from the National Heart Lung and Blood Institute[1]

Table S8: Strategy 1 and 2 Visit Completion Frequency Before and During the COVID-19 Pandemic

Visit	Strategy 1		Strategy 2	
	Before	During	Before	During
1	97.8% (44/45)	94.5% (52/55)	100.0% (42/42)	88.1% (52/59)
2	97.8% (44/45)	89.1% (49/55)	97.6% (41/42)	83.1% (49/59)
3	97.8% (44/45)	87.3% (48/55)	97.6% (41/42)	83.1% (49/59)
4	95.6% (43/45)	80.0% (44/55)	95.2% (40/42)	76.3% (45/59)
5	97.8% (44/45)	76.4% (42/55)	100.0% (42/42)	76.3% (45/59)
6	100.0% (45/45)	80.0% (44/55)	100.0% (42/42)	79.7% (47/59)

Strategy 1: In-store education only. Strategy 2: In-store education plus online training and tools. % represents completion by number of subgroup participants divided by the subgroup total. Overall totals for Control (N=46), Strategy 1 (N=100), and Strategy 2 (N=101).

TABLE S9: Dietary Intake and Biometrics Data Collection Before and During the COVID-19 Pandemic

Assessment	Control		Strategy 1		Strategy 2	
	Before	During	Before	During	Before	During
Dietary intake at 3 months	100.0% (22/22)	83.3% (20/24)	100.0% (45/45)	85.5% (47/55)	100.0% (42/42)	83.1% (49/59)
Biometrics at 3 months	100.0% (22/22)	70.8% (17/24)	100.0% (45/45)	80.0% (44/55)	100.0% (42/42)	81.4% (48/59)
Dietary intake at 6 months	100.0% (22/22)	95.8% (23/24)	93.3% (42/45)	94.5% (52/55)	97.6% (41/42)	94.9% (56/59)
Biometrics at 6 months	100.0% (22/22)	83.3% (20/24)	93.3% (42/45)	90.9% (50/55)	90.5% (38/42)	84.7% (50/59)

Strategy 1: In-store education only. Strategy 2: In-store education plus online training and tools. % represents completion by number of subgroup participants divided by the subgroup total. Biometrics include blood pressure, weight, hip-to-waist ratio, and laboratory measurements. Overall totals for Control (N=46), Strategy 1 (N=100), and Strategy 2 (N=101).

Table S10: DASH Score Components at Baseline and 6 Months

	Control (n=46) Mean (95%CI)	Strategy 1 (n=100) Mean (95%CI)	Strategy 2 (n=101) Mean (95%CI)	Primary Comparison (CI) Strategies 1 and 2 vs. Control Mean (95%CI)	P-value	Secondary Comparison (CI) Strategy 2 vs. 1 Mean (95%CI)	P-value
**Fruit servings/1000kcal/day**							
At baseline	1.8 (1.3, 2.3)	1.6 (1.3, 2.0)	1.3 (1.0, 1.7)				
At 6 mon	1.8 (1.3, 2.3)	2.0 (1.6, 2.3)	2.2 (1.9, 2.6)				
Change (baseline to 6 mon)	-0.0 (-0.6, 0.5)	0.4 (-0.0, 0.7)	0.9 (0.5, 1.3)	0.7 (0.1, 1.3)	0.03	0.5 (0.0, 1.1)	0.04
**Vegetable servings/1000kcal/day**							
At baseline	3.3 (2.7, 3.8)	2.7 (2.3, 3.1)	2.7 (2.3, 3.1)				
At 6 mon	2.8 (2.3, 3.4)	3.1 (2.7, 3.5)	3.2 (2.8, 3.6)				
Change (baseline to 6 mon)	-0.4 (-1.1, 0.2)	0.4 (-0.1, 0.9)	0.5 (0.0, 0.9)	0.9 (0.1, 1.6)	0.02	0.1 (-0.6, 0.7)	0.88
**Total Dairy servings/1000kcal/day**							
At baseline	1.7 (1.4, 2.0)	1.7 (1.5, 1.9)	1.6 (1.4, 1.8)				
At 6 mon	1.4 (1.1, 1.8)	1.7 (1.5, 1.9)	1.4 (1.2, 1.6)				
Change (baseline to 6 mon)	-0.2 (-0.6, 0.2)	-0.0 (-0.3, 0.3)	-0.2 (-0.5, 0.1)	0.1 (-0.3, 0.6)	0.54	-0.2 (-0.6, 0.2)	0.32
**Low Fat Dairy servings/1000kcal/day**							
At baseline	0.3 (0.2, 0.5)	0.3 (0.2, 0.4)	0.3 (0.1, 0.4)				
At 6 mon	0.4 (0.3, 0.6)	0.6 (0.4, 0.7)	0.5 (0.3, 0.6)				
Change (baseline to 6 mon)	0.1 (-0.1, 0.3)	0.2 (0.1, 0.4)	0.2 (0.1, 0.3)	0.1 (-0.1, 0.3)	0.39	-0.1 (-0.3, 0.1)	0.55
**Total Grain servings/1000kcal/day**							
At baseline	5.5 (4.8, 6.2)	5.4 (4.9, 5.9)	5.0 (4.5, 5.5)				
At 6 mon	4.8 (4.1, 5.5)	5.0 (4.5, 5.5)	4.5 (4.0, 5.1)				
Change (baseline to 6 mon)	-0.7 (-1.5, 0.1)	-0.4 (-0.9, 0.2)	-0.4 (-1.0, 0.1)	0.3 (-0.6, 1.2)	0.48	-0.1 (-0.8, 0.7)	0.87
**Whole Grain servings/1000kcal/day**							
At baseline	1.6 (1.2, 2.0)	1.3 (1.0, 1.6)	1.2 (0.9, 1.5)				
At 6 mon	1.5 (1.1, 1.9)	1.9 (1.6, 2.2)	1.9 (1.6, 2.2)				
Change (baseline to 6 mon)	-0.1 (-0.6, 0.4)	0.6 (0.3, 1.0)	0.7 (0.4, 1.1)	0.8 (0.2, 1.3)	<.01	0.1 (-0.4, 0.6)	0.73

	Control (n=46) Mean (95%CI)	Strategy 1 (n=100) Mean (95%CI)	Strategy 2 (n=101) Mean (95%CI)	Primary Comparison (CI) Strategies 1 and 2 vs. Control Mean (95%CI)	P-value	Secondary Comparison (CI) Strategy 2 vs. 1 Mean (95%CI)	P-value
**Meat servings/1000kcal/day**							
At baseline	5.0 (4.2, 5.7)	5.1 (4.6, 5.6)	5.5 (4.9, 6.0)				
At 6 mon	4.8 (4.0, 5.5)	5.0 (4.5, 5.6)	4.7 (4.2, 5.3)				
Change (baseline to 6 mon)	-0.2 (-1.0, 0.7)	-0.1 (-0.7, 0.5)	-0.7 (-1.3, -0.2)	-0.2 (-1.2, 0.7)	0.65	-0.7 (-1.5, 0.2)	0.11
**Nuts/seeds servings/1000kcal/day**							
At baseline	0.9 (0.5, 1.4)	1.1 (0.8, 1.4)	1.1 (0.8, 1.4)				
At 6 mon	1.3 (0.9, 1.8)	1.1 (0.8, 1.5)	1.1 (0.7, 1.4)				
Change (baseline to 6 mon)	0.4 (-0.1, 1.0)	0.1 (-0.3, 0.4)	-0.1 (-0.4, 0.3)	-0.4 (-1.0, 0.2)	0.17	-0.1 (-0.7, 0.4)	0.60
**Sweets servings/1000kcal/day**							
At baseline	2.5 (1.9, 3.0)	2.3 (1.9, 2.7)	2.5 (2.1, 2.8)				
At 6 mon	2.1 (1.6, 2.6)	1.7 (1.3, 2.1)	1.8 (1.4, 2.2)				
Change (baseline to 6 mon)	-0.4 (-1.0, 0.3)	-0.7 (-1.1, -0.2)	-0.7 (-1.1, -0.2)	-0.3 (-1.0, 0.4)	0.42	-0.0 (-0.7, 0.6)	0.90
**Fats/oils servings/1000kcal/day**							
At baseline	4.0 (3.4, 4.5)	3.6 (3.1, 4.0)	3.7 (3.3, 4.1)				
At 6 mon	3.2 (2.6, 3.8)	3.2 (2.8, 3.7)	2.8 (2.4, 3.3)				
Change (baseline to 6 mon)	-0.8 (-1.5, -0.1)	-0.3 (-0.8, 0.2)	-0.9 (-1.3, -0.4)	0.2 (-0.6, 1.0)	0.62	-0.5 (-1.2, 0.2)	0.13
**Sodium mg/1000kcal/day**							
At baseline	2836.4 (2580.5, 3092.4)	2773.5 (2585.9, 2961.1)	2633.8 (2447.0, 2820.6)				
At 6 mon	2441.0 (2182.3, 2699.7)	2395.8 (2204.9, 2586.6)	2172.4 (1983.1, 2361.7)				
Change (baseline to 6 mon)	-395.4 (-699.6, -91.3)	-377.8 (-587.7, -167.8)	-461.4 (-666.1, -256.7)	-24.1 (-360.8, 312.5)	0.89	-83.7 (-380.7, 213.3)	0.58

Strategy 1: In-store education only. Strategy 2: In-store education plus online training and tools. A mixed model for repeated measures was used to model each outcome, controlling for age-group, sex, household size, race, income and baseline BMI. Contrasts (with two-sided t-tests) were used to estimate differences between treatment groups for 6-month change from baseline. Intervention effect is difference in mean change in outcome and 95% confidence interval. No adjustments for multiple comparisons were made.

Table S11: Other Nutritional Measurements at Baseline and 3 Months

	Control (n=46) Mean (95%CI)	Strategy 1 (n=100) Mean (95%CI)	Strategy 2 (n=101) Mean (95%CI)	Primary Comparison (CI) Strategies 1 and 2 vs. Control Mean (95%CI)	P-value	Secondary Comparison (CI) Strategy 2 vs. 1 Mean (95%CI)	P-value
**Energy Intake - kcal/day**							
At baseline	1809.8 (1668.8, 1950.8)	1773.1 (1669.2, 1877.1)	1771.3 (1667.7, 1874.8)				
At 3 mon	1534.7 (1386.6, 1682.8)	1545.5 (1439.6, 1651.4)	1549.6 (1443.2, 1656.0)				
Change (baseline to 3 mon)	-275.1 (-409.9, -140.3)	-227.6 (-315.7, -139.4)	-221.7 (-308.8, -134.5)	50.5 (-104.0, 205.0)	0.52	5.9 (-116.2, 128.1)	0.92
**Total fat % of kcals/day**							
At baseline	36.9 (34.6, 39.1)	36.5 (34.8, 38.1)	37.1 (35.5, 38.8)				
At 3 mon	33.3 (30.9, 35.6)	32.0 (30.3, 33.7)	32.0 (30.3, 33.6)				
Change (baseline to 3 mon)	-3.6 (-5.8, -1.4)	-4.5 (-6.0, -3.0)	-5.2 (-6.6, -3.7)	-1.2 (-3.7, 1.3)	0.34	-0.7 (-2.7, 1.4)	0.52
**Saturated Fat % of kcals/day**							
At baseline	11.7 (10.7, 12.7)	11.6 (10.9, 12.4)	11.7 (11.0, 12.5)				
At 3 mon	10.3 (9.2, 11.3)	9.4 (8.7, 10.1)	9.5 (8.8, 10.3)				
Change (baseline to 3 mon)	-1.4 (-2.4, -0.4)	-2.2 (-2.9, -1.6)	-2.2 (-2.9, -1.6)	-0.8 (-2.0, 0.4)	0.17	0.0 (-0.9, 0.9)	0.97
**Calcium mg/1000kcal/day**							
At baseline	820.0 (730.5, 909.6)	799.2 (733.6, 864.9)	770.0 (704.7, 835.4)				
At 3 mon	734.0 (640.2, 827.8)	827.5 (759.9, 895.2)	816.1 (748.7, 883.6)				
Change (baseline to 3 mon)	-86.0 (-179.7, 7.7)	28.3 (-34.5, 91.1)	46.1 (-15.2, 107.4)	123.2 (15.8, 230.7)	0.02	17.8 (-68.6, 104.1)	0.69
**Potassium mg/1000kcal/day**							
At baseline	2488.3 (2241.4, 2735.3)	2344.8 (2162.5, 2527.1)	2372.5 (2190.9, 2554.1)				
At 3 mon	2571.5 (2313.5, 2829.6)	2603.3 (2417.5, 2789.2)	2683.8 (2497.0, 2870.5)				
Change (baseline to 3 mon)	83.2 (-147.0, 313.4)	258.5 (106.6, 410.4)	311.3 (161.5, 461.2)	201.7 (-62.2, 465.7)	0.13	52.8 (-157.3, 263.0)	0.62
**Magnesium mg/1000kcal/day**							
At baseline	287.7 (258.0, 317.5)	269.6 (247.6, 291.6)	277.3 (255.4, 299.1)				
At 3 mon	289.1 (258.0, 320.2)	297.8 (275.5, 320.2)	315.6 (293.1, 338.1)				
Change (baseline to 3 mon)	1.4 (-26.3, 29.1)	28.2 (10.0, 46.4)	38.4 (20.3, 56.4)	31.9 (0.2, 63.6)	0.05	10.1 (-15.1, 35.4)	0.43

Strategy 1: In-store education only. Strategy 2: In-store education plus online training and tools. A mixed model for repeated measures was used to model each outcome, controlling for age-group, sex, household size, race, income and baseline BMI. Contrasts (with two-sided t-tests) were used to estimate differences between treatment groups a) at baseline and b) for 3-month change from baseline. No adjustments for multiple comparisons were made.

Table S12: Other Nutritional Measurements at Baseline and 6 Months

	Control (n=46) Mean (95%CI)	Strategy 1 (n=100) Mean (95%CI)	Strategy 2 (n=101) Mean (95%CI)	Primary Comparison (CI) Strategies 1 and 2 vs. Control Mean (95%CI)	P-value	Secondary Comparison (CI) Strategy 2 vs. 1 Mean (95%CI)	P-value
**Energy Intake - kcal/day**							
At baseline	1809.8 (1668.8, 1950.8)	1773.1 (1669.2, 1877.1)	1771.3 (1667.7, 1874.8)				
At 6 mon	1618.7 (1476.5, 1761.0)	1628.1 (1522.9, 1733.2)	1531.2 (1426.3, 1636.1)				
Change (baseline to 6 mon)	-191.1 (-347.3, -34.9)	-145.1 (-252.5, -37.6)	-240.0 (-345.4, -134.7)	-1.5 (-174.3, 171.3)	0.99	-95.0 (-247.4, 57.4)	0.22
**Total fat % of kcals/day**							
At baseline	36.9 (34.6, 39.1)	36.5 (34.8, 38.1)	37.1 (35.5, 38.8)				
At 6 mon	35.2 (33.0, 37.5)	33.4 (31.7, 35.1)	31.7 (30.1, 33.4)				
Change (baseline to 6 mon)	-1.6 (-4.2, 0.9)	-3.1 (-4.9, -1.3)	-5.4 (-7.1, -3.7)	-2.6 (-5.4, 0.2)	0.07	-2.3 (-4.8, 0.2)	0.07
**Saturated Fat % of kcals/day**							
At baseline	11.7 (10.7, 12.7)	11.6 (10.9, 12.4)	11.7 (11.0, 12.5)				
At 6 mon	10.7 (9.7, 11.7)	10.2 (9.5, 11.0)	9.5 (8.7, 10.2)				
Change (baseline to 6 mon)	-1.0 (-2.1, 0.2)	-1.4 (-2.2, -0.6)	-2.3 (-3.1, -1.5)	-0.9 (-2.1, 0.4)	0.19	-0.9 (-2.0, 0.3)	0.13
**Calcium mg/1000kcal/day**							
At baseline	820.0 (730.5, 909.6)	799.2 (733.6, 864.9)	770.0 (704.7, 835.4)				
At 6 mon	766.2 (675.9, 856.6)	836.3 (769.3, 903.3)	766.5 (700.2, 832.9)				
Change (baseline to 6 mon)	-53.8 (-160.0, 52.4)	37.1 (-36.8, 111.0)	-3.5 (-75.2, 68.2)	70.6 (-47.1, 188.3)	0.24	-40.6 (-145.0, 63.8)	0.45
**Potassium mg/1000kcal/day**							
At baseline	2488.3 (2241.4, 2735.3)	2344.8 (2162.5, 2527.1)	2372.5 (2190.9, 2554.1)				
At 6 mon	2481.1 (2232.2, 2730.0)	2587.3 (2402.7, 2771.9)	2440.5 (2256.5, 2624.4)				
Change (baseline to 6 mon)	-7.3 (-276.8, 262.2)	242.5 (56.6, 428.4)	68.0 (-113.9, 249.9)	162.5 (-135.7, 460.8)	0.29	-174.5 (-438.1, 89.1)	0.19
**Magnesium mg/1000kcal/day**							
At baseline	287.7 (258.0, 317.5)	269.6 (247.6, 291.6)	277.3 (255.4, 299.1)				
At 6 mon	284.4 (254.3, 314.4)	299.8 (277.6, 322.0)	281.7 (259.5, 303.8)				
Change (baseline to 6 mon)	-3.4 (-35.8, 29.1)	30.2 (7.9, 52.5)	4.4 (-17.5, 26.3)	20.7 (-15.2, 56.6)	0.26	-25.8 (-57.4, 5.8)	0.11

Strategy 1: In-store education only. Strategy 2: In-store education plus online training and tools. A mixed model for repeated measures was used to model each outcome, controlling for age-group, sex, household size, race, income and baseline BMI. Contrasts (with two-sided t-tests) were used to estimate differences between treatment groups a) at baseline and c) for 6-month change from baseline. No adjustments for multiple comparisons were made.

Table S13: Medication Treatment at Baseline and 6 Months

Variable	Control (n=46) Mean (95%CI)	Strategy 1 (n=100) Mean (95%CI)	Strategy 2 (n=101) Mean (95%CI)	Primary Comparison (CI) Strategies 1 and 2 vs. Control Mean (95%CI)	P-value	Secondary Comparison (CI) Strategy 2 vs. 1 Mean (95%CI)	P-value
**Number of Total Medications[a]**							
At baseline	1.41 (0.99, 1.82)	1.71 (1.40, 2.02)	1.40 (1.09, 1.71)				
At 6 mon	1.38 (0.96, 1.81)	1.56 (1.25, 1.88)	1.36 (1.05, 1.68)				
Change (baseline to 6 mon)	-0.02 (-0.23, 0.19)	-0.15 (-0.29, -0.01)	-0.04 (-0.17, 0.10)	-0.07 (-0.30, 0.16)	0.54	0.11 (-0.08, 0.31)	0.26
**Number of Blood Pressure Meds[a]**							
At baseline	1.04 (0.69, 1.38)	1.27 (1.01, 1.53)	1.10 (0.84, 1.36)				
At 6 mon	0.97 (0.62, 1.32)	1.10 (0.84, 1.36)	1.03 (0.84, 1.36)				
Change (baseline to 6 mon)	-0.06 (-0.24, 0.11)	-0.17 (-0.28, -0.05)	-0.07 (-0.19, 0.05)	-0.05 (-0.25, 0.14)	0.59	0.09 (-0.07, 0.26)	0.27
**Number of Cholesterol Meds[a]**							
At baseline	0.37 (0.19, 0.55)	0.44 (0.31, 0.58)	0.30 (0.16, 0.43)				
At 6 mon	0.41 (0.24, 0.59)	0.46 (0.33, 0.59)	0.33 (0.20, 0.46)				
Change (baseline to 6 mon)	0.04 (-0.03, 0.12)	0.02 (-0.04, 0.07)	0.03 (-0.02, 0.08)	-0.02 (-0.09, 0.05)	0.64	0.02 (-0.05, 0.09)	0.61

Strategy 1: In-store education only. Strategy 2: In-store education plus online training and tools. For continuous measurements: a mixed model for repeated measures was used to model each outcome, controlling for age-group, sex, race, household size, income, baseline SBP, or baseline total cholesterol. Contrasts (with two-sided t-tests) were used to estimate differences between treatment groups a) at baseline, and b) for 6-month change from baseline. [a] Intervention effect is difference in mean change in outcome and 95% confidence interval. No adjustments for multiple comparisons were made.

Table S14: Systolic and Diastolic Blood Pressure, Body-Mass Index at Baseline and 3 Months

	Control (n=46) Mean (95%CI)	Strategy 1 (n=100) Mean (95%CI)	Strategy 2 (n=101) Mean (95%CI)	Primary Comparison (CI) Strategies 1 and 2 vs. Control	P-value	Secondary Comparison (CI) Strategy 2 vs. 1	P-value
**Systolic blood pressure[a] – mmHg**							
At baseline	125.9 (119.1, 132.7)	125.6 (119.7, 131.5)	125.0 (119.0, 130.9)				
At 3 months	123.2 (116.2, 130.1)	118.9 (113.0, 124.9)	119.2 (113.3, 125.2)				
Change (baseline to 3 months)	-2.8 (-7.1, 1.6)	-6.6 (-9.8, -3.4)	-5.7 (-8.7, -2.8)	-3.4 (-8.4, 1.6)	0.18	0.9 (-3.2, 5.0)	0.66
**Diastolic blood pressure[a] - mmHg**							
At baseline	82.8 (78.2, 87.5)	79.2 (75.1, 83.2)	81.4 (77.3, 85.6)				
At 3 months	80.2 (75.5, 84.9)	76.7 (72.6, 80.9)	79.4 (75.1, 83.7)				
Change (baseline to 3 months)	-2.6 (-5.5, 0.2)	-2.4 (-4.2, -0.6)	-2.0 (-3.9, -0.1)	0.4 (-2.7, 3.6)	0.79	0.4 (-2.1, 2.9)	0.76
**Hypertension control at 3 months[b] - N (%)**							
SBP <130 and DBP <80	9 (23%)	41 (46%)	32 (36%)	1.47 (0.83, 2.61)	0.19	0.75 (0.52, 1.06)	0.11
SBP <140 and DBP <90	22 (56%)	68 (76%)	66 (73%)	1.22 (0.90, 1.66)	0.19	0.88 (0.68, 1.15)	0.35
Strategy 1: In-store education only. Strategy 2: In-store education plus online training and tools. For systolic blood pressure and diastolic blood pressure: a mixed model for repeated measures was used to model each outcome, controlling for age-group, sex, race, household size, income, baseline BMI, baseline BP meds (0, 1, 2+), baseline sedentary (yes/no), and baseline current smoking (yes/no). Contrasts (with two-sided t-tests) were used to estimate differences between treatment groups a) at baseline and b) for 3-month change from baseline. For hypertension control, relative risk was calculated using logistic regression models, controlling for the baseline value of the dependent variable (e.g. SBP, DBP), age-group, sex, race, baseline BMI, baseline BP meds (0, 1, 2+), baseline sedentary (yes/no), and baseline current smoking (yes/no). Data represent least-squares means (95% CI). [a] Intervention effect is difference in mean change in blood pressure and 95% confidence interval. [b] Percentages for each group are calculated from the non-missing totals (Control= 39, Strategy 1=89, Strategy 2= 90). Intervention effect is the relative risk and 95% confidence interval. No adjustments for multiple comparisons were made.							
**BMI[a] - kg/m^2**							
At baseline	37.9 (34.2, 41.7)	38.1 (34.8, 41.4)	37.1 (33.7, 40.5)				
At 3 months	37.7 (33.9, 41.4)	37.7 (34.3, 41.0)	36.3 (32.9, 39.8)				
Change (baseline to 3 months)	-0.2 (-0.6, 0.1)	-0.4 (-0.7, -0.2)	-0.8 (-1.0, -0.5)	-0.4 (-0.8, 0.0)	0.08	-0.3 (-0.7, 0.0)	0.06
**BMI achievement at 3 months[b] - N (%)**							
BMI <30	11 (28%)	36 (40%)	46 (51%)	1.25 (0.65, 2.39)	0.50	0.99 (0.58, 1.67)	0.97
BMI <40	29 (74%)	77 (87%)	81 (90%)	1.01 (0.66, 1.52)	0.98	0.96 (0.60, 1.54)	0.88
For BMI, a mixed model for repeated measures was used to model each outcome, controlling for age-group, sex, household size, race, income, sedentary (yes/no), current smoking (yes/no), and baseline energy intake. Contrasts (with two-sided t-tests) were used to estimate differences between treatment groups a) at baseline and b) for 3-month change from baseline. For BMI achievement, relative risk was calculated using logistic regression models, controlling for the baseline value of BMI, age-group, sex, household size, race, income, baseline sedentary (yes/no), baseline current smoking (yes/no), and baseline energy intake. Data represent least-squares means (95% CI). [a] Intervention effect is difference in mean change in outcome and 95% confidence interval. [b] Percentages for each group are calculated from the non-missing totals (Control= 39, Strategy 1= 89, Strategy 2= 90). Intervention effect is the relative risk and 95% confidence interval. No adjustments for multiple comparisons were made.							

Table S15: Systolic Blood Pressure, Diastolic Blood Pressure, and Body-Mass Index at Baseline and 6 Months

	Control (n=46) Mean (95%CI)	Strategy 1 (n=100) Mean (95%CI)	Strategy 2 (n=101) Mean (95%CI)	Primary Comparison (CI) Strategies 1 and 2 vs. Control	P-value	Secondary Comparison (CI) Strategy 2 vs. 1	P-value
**Systolic blood pressure[a] – mmHg**							
At baseline	125.9 (119.1, 132.7)	125.6 (119.7, 131.5)	125.0 (119.0, 130.9)				
At 6 months	120.5 (113.6, 127.5)	120.4 (114.3, 126.4)	120.8 (114.7, 126.9)				
Change (baseline to 6 months)	-5.4 (-10.7, -0.1)	-5.2 (-8.8, -1.6)	-4.2 (-7.8, -0.5)	0.7 (-5.1, 6.6)	0.81	1.1 (-4.0, 6.1)	0.68
**Diastolic blood pressure[a] - mmHg**							
At baseline	82.8 (78.2, 87.5)	79.2 (75.1, 83.2)	81.4 (77.3, 85.6)				
At 6 months	79.2 (74.3, 84.0)	74.7 (70.6, 78.8)	79.4 (75.1, 83.7)				
Change (baseline to 6 months)	--3.7 (-7.1, -0.3)	-4.4 (-6.7, -2.2)	-2.0 (-4.3, 0.4)	0.5 (-3.3, 4.3)	0.80	2.5 (-0.8, 5.7)	0.14
**Hypertension control[b] at 6 months - N (%)**							
SBP <130 and DBP <80	13 (31%)	42 (46%)	34 (39%)	1.15 (0.72, 1.85)	0.56	0.87 (0.6, 1.25)	0.46
SBP <140 and DBP <90	29 (69%)	71 (77%)	61 (69%)	1.02 (0.77, 1.36)	0.89	0.98 (0.77, 1.25)	0.86

Strategy 1: In-store education only. Strategy 2: In-store education plus online training and tools. SBP: systolic blood pressure; DBP: diastolic blood pressure. For systolic blood pressure and diastolic blood pressure: a mixed model for repeated measures was used to model each outcome, controlling for age-group, sex, race, household size, income, baseline BMI, baseline BP meds (0, 1, 2+), baseline sedentary (yes/no), and baseline current smoking (yes/no). Contrasts (with two-sided t-tests) were used to estimate differences between treatment groups a) at baseline and b) for 6-month change from baseline. For hypertension control, relative risk was calculated using logistic regression models, controlling for the baseline value of the dependent variable (e.g. SBP, DBP), age-group, sex, race, baseline BMI, baseline BP meds (0, 1, 2+), baseline sedentary (yes/no), and baseline current smoking (yes/no). Data represent least-squares means (95% CI). [a] Intervention effect is difference in mean change in blood pressure and 95% confidence interval. [b] Percentages for each group are calculated from the non-missing totals (Control=39, Strategy 1=89, Strategy 2=90). Intervention effect is the relative risk and 95% confidence interval. No adjustments for multiple comparisons were made.

	Control	Strategy 1	Strategy 2	Primary Comparison (CI)	P-value	Secondary Comparison (CI)	P-value
**BMI[a] - kg/m²**							
At baseline	37.9 (34.2, 41.7)	38.1 (34.8, 41.4)	37.1 (33.7, 40.5)				
At 6 months	37.7 (33.9, 41.4)	37.5 (34.1, 40.8)	36.3 (32.9, 39.8)				
Change (baseline to 6 months)	-0.3 (-0.8, 0.2)	-0.6 (-0.9, -0.3)	-0.7 (-1.1, -0.4)	-0.4 (-1.0, 0.1)	0.14	-0.1 (-0.6, 0.3)	0.58
**BMI achievement[b] at 6 months - N (%)**							
BMI <30	12 (29%)	35 (38%)	43 (49%)	1.25 (0.67, 2.37)	0.48	1.05 (0.63, 1.72)	0.85
BMI <40	33 (79%)	78 (85%)	76 (86%)	0.99 (0.66, 1.49)	0.97	0.95 (0.63, 1.45)	0.83

BMI: body-mass index. For BMI, a mixed model for repeated measures was used to model each outcome, controlling for age-group, sex, household size, race, income, sedentary (yes/no), current smoking (yes/no), and baseline energy intake. Contrasts (with two-sided t-tests) were used to estimate differences between treatment groups a) at baseline and b) for 6-month change from baseline. For BMI achievement, relative risk was calculated using logistic regression models, controlling for the baseline value of BMI, age-group, sex, household size, race, income, baseline sedentary (yes/no), baseline current smoking (yes/no), and baseline energy intake. Data represent least-squares means (95% CI). [a] Intervention effect is difference in mean change in outcome and 95% confidence interval. [b] Percentages for each group are calculated from the non-missing totals (Control=39, Strategy 1=89, Strategy 2=90). Intervention effect is the relative risk and 95% confidence interval. No adjustments for multiple comparisons were made.

Table S16: Non-HDL Cholesterol, Total Cholesterol, and Triglycerides at Baseline and 3 Months

	Control (n=46) Mean (95%CI)	Strategy 1 (n=100) Mean (95%CI)	Strategy 2 (n=101) Mean (95%CI)	Primary Comparison (CI) Strategies 1 and 2 vs. Control	P-value	Secondary Comparison (CI) Strategy 2 vs. 1	P-value
**Non-HDL-C[a] – mg/dl**							
At baseline	107.3 (95.3, 119.4)	116.6 (107.1, 126.1)	110.6 (101.0, 120.1)				
At 3 months	111.0 (98.2, 123.8)	113.3 (103.8, 122.9)	115.5 (106.0, 125.1)				
Change (baseline to 3 months)	3.7 (-5.7, 13.0)	-3.2 (-9.1, 2.6)	4.9 (-0.9, 10.8)	-2.8 (-13.3, 7.7)	0.60	8.2 (0.0, 16.4)	0.05
**Non-HDL-C control at 3 months[b] - N (%)**							
Non-HDL-C <100	13 (33%)	37 (42%)	27 (30%)	1.19 (0.77, 1.85)	0.44	0.86 (0.55, 1.37)	0.53
Non-HDL-C <130	29 (74%)	64 (72%)	58 (64%)	0.91 (0.62, 1.32)	0.62	0.97 (0.78, 1.2)	0.79
**Total Cholesterol[a] – mg/dl**							
At baseline	154.6 (141.6, 167.5)	164.6 (154.3, 174.9)	160.3 (150.0, 170.7)				
At 3 months	159.4 (145.7, 173.0)	160.8 (150.5, 171.1)	164.7 (154.4, 175.1)				
Change (baseline to 3 months)	4.8 (-4.7, 14.2)	-3.8 (-9.7, 2.1)	4.4 (-1.6, 10.4)	-4.5 (-15.0, 6.0)	0.40	8.2 (-0.0, 16.4)	0.05
**Triglycerides[#] - mg/dl**							
At baseline	147.3 (121.1, 179.2)	148.6 (127.2, 173.7)	139.4 (119.2, 163.0)				
At 3 months	151.5 (122.0, 188.2)	141.8 (121.1, 166.1)	130.6 (111.1, 153.7)				
% of reference (baseline to 3 months)*	102.9 (86.8, 121.9)	95.4 (86.8, 104.9)	93.7 (84.7, 103.7)	91.9 (75.4, 112.0)	0.39	98.2 (85.8, 112.5)	0.79
**TG control at 3 months - N (%)[b]**							
TG <100	10 (26%)	19 (21%)	40 (44%)	1.04 (0.57, 1.89)	0.89	1.33 (0.88, 2.04)	0.18
TG <150	17 (44%)	44 (49%)	58 (64%)	1.22 (0.83, 1.80)	0.30	1.11 (0.83, 1.47)	0.49

Strategy 1: In-store education only. Strategy 2: In-store education plus online training and tools. Non-HDL-C: non-high-density lipoprotein cholesterol; TG: triglycerides. For continuous measurements: a mixed model for repeated measures was used to model each outcome, controlling for age-group, sex, race, household size, income, baseline BMI, baseline cholesterol meds (no/yes), and baseline sedentary (yes/no). Contrasts (with two-sided t-tests) were used to estimate differences between treatment groups a) at baseline and b) for 6-month change from baseline. For categorical control variables: relative risk was calculated using logistic regression models, controlling for the baseline value of the dependent variable (e.g. non-HDL-C, total cholesterol, TG), age-group, sex, household size, race, income, baseline sedentary (yes/no), baseline cholesterol meds (no/yes), and baseline BMI. [a] Intervention effect is difference in mean change in outcome and 95% confidence interval. [b] Percentages for each group are calculated from the non-missing totals (Control=39, Strategy 1=89, Strategy 2=90). [#] Modeling for triglycerides based on log-transformed data. Values for groups at all timepoints represent medians (geometric means) and confidence intervals. * Values for comparisons (within and across groups) represent percentage of reference value and confidence intervals. Intervention effect is the relative risk and 95% confidence interval. No adjustments for multiple comparisons were made.

Table S17: Non-HDL Cholesterol, Total Cholesterol, and Triglycerides at Baseline and 6 Months

	Control (n=46) Mean (95%CI)	Strategy 1 (n=100) Mean (95%CI)	Strategy 2 (n=101) Mean (95%CI)	Primary Comparison (CI) Strategies 1 and 2 vs. Control	P-value	Secondary Comparison (CI) Strategy 2 vs. 1	P-value
**Non-HDL-C[a] – mg/dl**							
At baseline	107.3 (95.3, 119.4)	116.6 (107.1, 126.1)	110.6 (101.0, 120.1)				
At 6 months	113.5 (101.2, 125.8)	112.9 (103.2, 122.5)	112.1 (102.4, 121.8)				
Change (baseline to 6 months)	6.1 (-4.9, 17.2)	-3.7 (-11.1, 3.7)	1.5 (-5.8, 8.8)	-7.2 (-19.7, 5.3)	0.26	5.2 (-5.1, 15.5)	0.32
**Non-HDL-C control[b] at 6 months – N (%)**							
NonHDL-C <100	14 (33%)	35 (38%)	30 (34%)	1.15 (0.76, 1.76)	0.51	0.88 (0.58, 1.35)	0.57
NonHDL-C <130	28 (67%)	70 (76%)	62 (70%)	1.30 (1.06, 1.60)	0.01	0.91 (0.71, 1.16)	0.44
**Total Cholesterol[a] – mg/dl**							
At baseline	154.6 (141.6, 167.5)	164.6 (154.3, 174.9)	160.3 (150.0, 170.7)				
At 6 months	161.5 (148.3, 174.7)	162.4 (152.0, 172.9)	164.8 (154.3, 175.2)				
Change (baseline to 6 months)	6.9 (-4.5, 18.3)	-2.2 (-9.7, 5.4)	4.5 (-3.1, 12.0)	-5.8 (-18.6, 7.1)	0.38	6.6 (-4.0, 17.2)	0.22
**Triglycerides[#] - mg/dl**							
At baseline	147.3 (121.1, 179.2)	148.6 (127.2, 173.7)	139.4 (119.2, 163.0)				
At 6 months	148.8 (122.3, 181.1)	140.2 (120.0, 163.9)	139.9 (118.9, 164.6)				
Relative change (baseline to 6 months)*	101.0 (85.1, 120.0)	94.3 (83.9, 106.0)	100.3 (88.8, 113.4)	96.3 (79.6, 116.5)	0.70	106.4 (89.8, 126.1)	0.48
**TG control[b] at 6 months – N (%)**							
TG <100	9 (21%)	25 (27%)	37 (42%)	1.09 (0.62, 1.89)	0.77	1.1 (0.72, 1.67)	0.66
TG <150	23 (55%)	51 (55%)	57 (65%)	0.86 (0.63, 1.19)	0.37	1.02 (0.78, 1.33)	0.87

Strategy 1: In-store education only. Strategy 2: In-store education plus online training and tools. Non-HDL-C: non-high-density lipoprotein cholesterol; TG: triglycerides. For continuous measurements: a mixed model for repeated measures was used to model each outcome, controlling for age-group, sex, race, household size, income, baseline BMI, baseline cholesterol meds (no/yes), and baseline sedentary (yes/no). Contrasts (with two-sided t-tests) were used to estimate differences between treatment groups a) at baseline and b) for 6-month change from baseline. For categorical control variables: relative risk was calculated using logistic regression models, controlling for the baseline value of the dependent variable (e.g. non-HDL-C, total cholesterol, TG), age-group, sex, household size, race, income, baseline sedentary (yes/no), baseline cholesterol meds (no/yes), and baseline BMI. [a] Intervention effect is difference in mean change in outcome and 95% confidence interval. [#] Modeling for triglycerides based on log-transformed data. Values for groups at all timepoints represent medians (geometric means) and confidence intervals. [*] Values for comparisons (within and across groups) represent percentage of reference value and confidence intervals. [b] Percentages for each group are calculated from the non-missing totals (Control=39, Strategy 1=89, Strategy 2=90). Intervention effect is the relative risk and 95% confidence interval. No adjustments for multiple comparisons were made.

Table S18: Pre-COVID-19 Subgroup Analyses at Baseline and 3 Months

	Control (n=22) Mean (95%CI)	Strategy 1 (n=45) Mean (95%CI)	Strategy 2 (n=42) Mean (95%CI)	Primary Comparison (CI) Strategies 1 and 2 vs. Control (N=109)	P-value	Secondary Comparison (CI) Strategy 2 vs. 1 (N=87)	P-value
**DASH Score[a]**							
At baseline	45.1 (39.9, 50.4)	42.6 (38.6, 46.6)	42.7 (38.4, 47.0)				
At 3 months	48.9 (43.6, 54.2)	53.2 (49.2, 57.2)	56.4 (52.1, 60.7)				
Change (baseline to 3 months)				8.3 (3.4, 13.3)	0.001	3.1 (-1.3, 7.6)	0.17
**Systolic blood pressure[a] - mm Hg**							
At baseline	133.9 (123.3, 144.5)	134.1 (125.3, 142.9)	128.9 (119.3, 138.4)				
At 3 months	132.2 (121.5, 142.8)	124.9 (116.1, 133.7)	123.7 (114.1, 133.3)				
Change (baseline to 3 months)				-5.4 (-12.0, 1.2)	0.11	4.0 (-1.9, 10.0)	0.18
**Diastolic blood pressure[a] – mm Hg**							
At baseline	87.5 (80.2, 94.9)	85.1 (79.0, 91.2)	85.2 (78.6, 91.9)				
At 3 months	85.7 (78.4, 93.0)	80.7 (74.6, 86.8)	83.1 (76.5, 89.8)				
Change (baseline to 3 months)				-1.4 (-5.2, 2.5)	0.48	2.3 (-1.2, 5.7)	0.19
**BMI[a]**							
At baseline	34.5 (28.2, 40.8)	37.2 (32.0, 42.4)	35.1 (29.3, 40.8)				
At 3 months	34.3 (28.0, 40.6)	36.7 (31.5, 41.9)	34.3 (28.6, 40.1)				
Change (baseline to 3 months)				-0.4 (-0.9, 0.1)	0.11	-0.3 (-0.7, 0.2)	0.28
**Non-HDL-C[a] - mg/dl**							
At baseline	113.0 (93.3, 132.7)	122.3 (106.4, 138.2)	118.5 (101.7, 135.3)				
At 3 months	116.0 (96.2, 135.7)	117.8 (101.9, 133.7)	121.0 (104.2, 137.8)				
Change (baseline to 3 months)				-4.0 (-16.3, 8.4)	0.52	7.0 (-4.1, 18.1)	0.21
**Total cholesterol[a] - mg/dl**							
At baseline	163.7 (142.9, 184.5)	172.0 (155.3, 188.8)	166.3 (148.6, 184.1)				
At 3 months	164.8 (144.0, 185.6)	166.3 (149.5, 183.1)	167.1 (149.4, 184.8)				
Change (baseline to 3 months)				-3.6 (-16.8, 9.6)	0.59	6.5 (-5.3, 18.4)	0.28
**Triglycerides[#] - mg/dl**							
At baseline	130.1 (93.5, 181.1)	133.3 (102.0, 174.0)	132.8 (100.1, 176.1)				
At 3 months	137.5 (98.8, 191.4)	139.1 (106.5, 181.7)	123.7 (93.2, 164.1)				
Change (baseline to 3 months)*				93.3 (77.0, 113.0)	0.48	89.2 (75.1, 106)	0.19

Strategy 1: In-store education only. Strategy 2: In-store education plus online training and tools. BMI: Body-mass index. Modelling process for each outcome described in Tables 3 and 4 for each endpoint. [a] Data represent least-squares means (95% CI). Intervention effect is difference in mean change in outcome and 95% confidence interval. [#] Modeling for triglycerides based on log-transformed data. Values for groups at all timepoints represent medians (geometric means) and confidence intervals. * Values for comparisons (within and across groups) represent percentage of reference value and confidence intervals. Intervention effect is the relative risk and 95% confidence interval. Two-sided t-tests were used. No adjustments for multiple comparisons were made.

Table S19: Pre-COVID-19 Subgroup Analyses at Baseline and 6 Months

	Control (n=22) Mean (95%CI)	Strategy 1 (n=45) Mean (95%CI)	Strategy 2 (n=42) Mean (95%CI)	Primary Comparison (CI) Strategies 1 and 2 vs. Control (N=109)	P-value	Secondary Comparison (CI) Strategy 2 vs. 1 (N=87)	P-value
**DASH Score[a]**							
At baseline	45.1 (39.9, 50.4)	42.6 (38.6, 46.6)	42.7 (38.4, 47.0)				
At 6 months	49.8 (44.5, 55.1)	51.9 (47.8, 55.9)	53.1 (48.8, 57.5)				
Change (baseline to 6 months)				5.1 (-0.8, 11.1)	0.09	1.2 (-4.2, 6.6)	0.67
**Systolic blood pressure[a] - mm Hg**							
At baseline	133.9 (123.3, 144.5)	134.1 (125.3, 142.9)	128.9 (119.3, 138.4)				
At 6 months	129.2 (118.6, 139.9)	127.5 (118.7, 136.3)	124.9 (115.2, 134.5)				
Change (baseline to 6 months)				-0.6 (-8.8, 7.6)	0.89	2.6 (-4.9, 10.0)	0.50
**Diastolic blood pressure[a] - mm Hg**							
At baseline	87.5 (80.2, 94.9)	85.1 (79.0, 91.2)	85.2 (78.6, 91.9)				
At 6 months	83.6 (76.3, 90.9)	80.2 (74.1, 86.3)	83.3 (76.6, 90.0)				
Change (baseline to 6 months)				0.6 (-4.4, 5.5)	0.82	2.9 (-1.6, 7.4)	0.21
**BMI[a]- kg/m^2**							
At baseline	34.5 (28.2, 40.8)	37.2 (32.0, 42.4)	35.1 (29.3, 40.8)				
At 6 months	34.4 (28.1, 40.7)	36.6 (31.4, 41.8)	34.3 (28.5, 40.0)				
Change (baseline to 6 months)				-0.6 (-1.3, 0.1)	0.11	-0.2 (-0.9, 0.5)	0.58
**Non-HDL-C[a] - mg/dl**							
At baseline	113.0 (93.3, 132.7)	122.3 (106.4, 138.2)	118.5 (101.7, 135.3)				
At 6 months	123.2 (103.5, 142.9)	119.4 (103.4, 135.4)	120.8 (103.8, 137.8)				
Change (baseline to 6 months)				-10.5 (-26.3, 5.3)	0.20	5.2 (-9.2, 19.7)	0.48
**Total cholesterol[a] - mg/dl**							
At baseline	163.7 (142.9, 184.5)	172.0 (155.3, 188.8)	166.3 (148.6, 184.1)				
At 6 months	172.7 (151.9, 193.5)	168.6 (151.8, 185.5)	171.0 (153.1, 189.0)				
Change (baseline to 6 months)				-8.4 (-25.2, 8.5)	0.33	8.1 (-7.3, 23.5)	0.30
**Triglycerides[#] - mg/dl**							
At baseline	130.1 (93.5, 181.1)	133.3 (102.0, 174.0)	132.8 (100.1, 176.1)				
At 6 months	135.2 (97.2, 188.2)	130.1 (99.5, 170.2)	134.5 (101.1, 179.0)				
Change (baseline to 6 months)*				95.7 (74.6, 122.8)	0.73	103.7 (82.6, 130.2)	0.75

Strategy 1: In-store education only. Strategy 2: In-store education plus online training and tools. BMI: Body-mass index. Modelling process for each outcome described in Tables S9, S12, and S13 for each endpoint. [a] Data represent least-squares means (95% CI). Intervention effect is difference in mean change in outcome and 95% confidence interval. [#] Modeling for triglycerides based on log-transformed data. Values for groups at all timepoints represent medians (geometric means) and confidence intervals. * Values for comparisons (within and across groups) represent percentage of reference value and confidence intervals. Intervention effect is the relative risk and 95% confidence interval. Two-sided t-tests were used. No adjustments for multiple comparisons were made.

Table S20: Change in DASH Score for Subgroups at 3 Months

Subgroup	Strategies 1 and 2 Mean (95% CI)	Control Mean (95% CI)	Absolute Mean DASH Difference (compared to Control) Mean (95% CI)	p-value
Age				Interaction: <0.01
21-50	52.9 (49.6, 56.1)	56.7 (51.5, 61.9)	-3.9 (-10.4, 2.7)	0.24
51-75	56.6 (55.0, 58.2)	50.2 (46.6, 53.7)	6.4 (2.5, 10.3)	<0.01
Gender				Interaction: 0.64
Female	55.8 (54.0, 57.5)	52.9 (49.4, 56.4)	2.8 (-1.2, 6.9)	0.17
Male	55.4 (52.9, 57.9)	50.9 (45.3, 56.5)	4.5 (-1.7, 10.7)	0.15
Race				Interaction: <0.01
Not White	53.1 (50.4, 55.8)	58.3 (52.1, 64.5)	-5.2 (-12.0, 1.7)	0.14
White	56.6 (55.0, 58.3)	50.6 (47.3, 54.0)	6.0 (2.1, 9.9)	<0.01
Married				Interaction: 0.46
Yes	56.3 (54.5, 58.1)	52.0 (48.2, 55.8)	4.3 (-0.1, 8.6)	0.05
No	54.5 (52.1, 56.8)	52.8 (47.8, 57.8)	1.7 (-4.0, 7.4)	0.56
Education				Interaction: 0.40
Bachelor/Graduate	55.8 (54.0, 57.6)	53.4 (49.5, 57.3)	2.4 (-2.0, 6.8)	0.29
Not	55.4 (53.0, 57.7)	50.0 (44.9, 55.1)	5.4 (-0.2, 10.9)	0.06
Employment				Interaction: 0.88
Fulltime	54.4 (52.4, 56.5)	51.3 (47.3, 55.4)	3.1 (-1.5, 7.8)	0.19
Not Fulltime	57.0 (55.0, 59.1)	53.4 (49.0, 57.8)	3.6 (-1.3, 8.4)	0.15
Annual Household Income				Interaction: 0.56
< $100,000	55.7 (53.7, 57.8)	51.6 (47.9, 55.3)	4.1 (-0.1, 8.4)	0.06
≥ $100,000	55.5 (53.5, 57.6)	53.5 (48.2, 58.8)	2.1 (-3.8, 8.0)	0.49
Household Size				Interaction: 0.53
1-2 People	55.6 (53.9, 57.3)	52.9 (49.4, 56.4)	2.6 (-1.3, 6.6)	0.19
3 or More	55.8 (53.2, 58.5)	50.8 (45.0, 56.7)	5.0 (-1.7, 11.6)	0.14

Frequency of Cooking at Home				Interaction: 0.98
< 5-6x/week	54.4 (52.4, 56.4)	51.4 (47.6, 55.2)	3.0 (-1.6, 7.5)	0.20
≥ 5-6x/week	57.1 (55.1, 59.1)	54.1 (49.0, 59.2)	2.9 (-2.6, 8.4)	0.30
Activity level				Interaction: 0.16
Moderate/very	56.8 (55.1, 58.6)	51.6 (47.2, 55.9)	5.3 (0.6, 9.9)	0.03
Less	53.7 (51.1, 56.2)	53.0 (48.9, 57.2)	0.6 (-4.4, 5.6)	0.81
Hypercholesterolemia				Interaction: 0.37
No	54.7 (52.2, 57.3)	53.5 (48.6, 58.4)	1.3 (-4.2, 6.8)	0.65
Yes	56.1 (54.3, 57.8)	51.7 (47.7, 55.6)	4.4 (-0.1, 8.9)	0.05
Hypertension				Interaction: 0.01
No	54.8 (52.2, 57.4)	58.9 (52.8, 64.9)	-4.0 (-10.9, 2.8)	0.25
Yes	56.0 (54.3, 57.8)	50.2 (46.8, 53.7)	5.8 (1.9, 9.7)	<0.01
Obese				Interaction: 0.40
No	56.7 (54.5, 59.0)	55.8 (50.4, 61.2)	0.9 (-4.9, 6.8)	0.76
Yes	55.0 (53.0, 56.9)	50.9 (47.2, 54.6)	4.1 (-0.4, 8.6)	0.08
Baseline DASH Score				Interaction: 0.78
< 40	52.4 (49.9, 55.0)	48.1 (42.7, 53.6)	4.3 (-1.8, 10.4)	0.17
≥ 40	57.3 (55.6, 58.9)	53.9 (50.5, 57.4)	3.3 (-0.6, 7.2)	0.10

Strategy 1: In-store education only. Strategy 2: In-store education plus online training and tools. DASH score at 3 months was modeled (dependent variable) with a separate ANOVA model for each subgroup - including treatment-group (Control, Strategies 1 and 2) and subgroup x treatment-group interaction as additional terms. Imputed data was used for missing DASH scores. Data represent least-squares means (95% CI). Test for interaction effect was a two-sided F-test. Tests for differences between groups was a two-sided t-test. No adjustments for multiple comparisons were made.

Table S21: Study Supermarkets and Locations Used

Town	Address	City	State
Florence	7685 Mall Road	Florence	KY
Oakley	4613 Marburg Avenue	Oakley	OH
Lebanon	1425 Columbus Ave.	Lebanon	OH
Newport	130 Pavilion Drive	Newport	KY
Harrison	10477 Harrison Avenue	Harrison	OH
Amelia	262 West Main Street	Amelia	OH
Beckett Ridge	8000 Princeton-Glendale Road	West Chester	OH
Tylersville	7855 Tylersville Rd	Tylersville	OH
Mason	5100 Terra Firma Drive	Mason	OH
Old Liberty	5420 Liberty Fairfield Road	Liberty Township	OH
Fairfield	560 Wessel Dr	Fairfield	OH
Corryville	1 West Corry Street	Cincinnati	OH
Liberty Township Marketplace	7300 Yankee Rd	Liberty Township	OH

Supplementary Note 1: Study Protocol

UNIVERSITY OF CINCINNATI

INSTITUTIONAL REVIEW BOARD – MEDICAL IRB RESEARCH PROTOCOL

PRINCIPAL INVESTIGATORS (PIs): Dylan L. Steen, MD, MS and Sarah Couch, PhD, RDN

TITLE: Supermarket and Web-Based Intervention Targeting Nutrition (SuperWIN) for Cardiovascular Risk Reduction

All attachments are identified in the subsequent Protocol and are listed in Section 11.

ABSTRACT:

The Supermarket and Web-based Intervention targeting Nutrition (SuperWIN) for cardiovascular risk reduction is a novel, pilot, randomized controlled trial aimed at increasing diet quality and decreasing cardiovascular risk. SuperWIN will deliver individualized, nutrition education at the point-of-purchase (POP), either in the aisles of the physical store or via online shopping platforms coupled with other modern software tools. The trial is possible through a new partnership between UC/UC Health and The Kroger Company, the nation's largest supermarket chain.

The two interventions to be evaluated in SuperWIN will utilize either an in-store POP education strategy or a combination online/in-store POP education strategy in participants with at least one cardiovascular disease risk factor. The POP education interventions will be enhanced with sophisticated, provider- and patient-facing, food-purchasing metrics, a novel counseling tool that here-to-fore has not been studied in regard to changing food shopping behavior. Our primary outcome measure is the Dietary Approaches to Stop Hypertension (DASH) score, a validated measure of diet quality. The study is well-powered to test: 1) whether any POP education intervention improves diet quality and 2) whether an improvement in diet quality depends on the POP education strategy utilized. SuperWIN may serve as a foundational study for driving the design and testing of convenient, accessible, and affordable healthcare services delivered in settings that are conducive to behavior change.

1.SPECIFIC AIMS

This study is designed to test whether individualized, point-of-purchase (POP) nutrition education enhanced with

electronic food purchasing data can improve dietary quality. Two POP nutrition education strategies will be evaluated; an in-store POP strategy and a combined online/in-store POP strategy. The online portion of the second strategy will include online grocery shopping, online recipe resources, grocery pickup or home delivery, and a phone application to better select healthy foods. Both POP nutrition education strategies will be delivered within a supermarket-based retail clinic by a registered dietitian nutritionist (RDN). Both strategies will be offered in addition to a “standard of care” single nutrition counseling session. The POP nutrition education strategies will emphasize the DASH dietary pattern. This dietary pattern has been empirically tested and shown to favorably modify blood pressure, blood cholesterol, insulin sensitivity, and weight status. This study aims to answer the following research questions:

1) Does the addition of an individualized, supermarket-based, POP nutrition education intervention in addition to standard of care improve dietary quality compared to standard care alone?
2) Do achieved dietary quality measures depend on whether the POP nutrition education focuses on an in-store shopping versus a combined online/in-store (paired with other online tools) strategy?

2.BACKGROUND & SIGNIFICANCE

The prevalence of chronic diseases, and chronic disease risk factors, is increasing [1] largely due to changing dietary habits [2]. These diet-driven diseases are contributing to unsustainable healthcare spending, along with tremendous direct and indirect costs to the economy [3]. Prior research has demonstrated that effective implementation of evidence-based dietary interventions results in improved public health, both by reducing new cases of disease as well as reducing the impact on those with pre-existing disease [2]. Unfortunately, adoption and adherence to many of these interventions has been limited in the general U.S. population [4].

There are many consumer barriers to purchasing and preparing healthier meals. These include busy lifestyles, inadequate personal or public transportation, distance to the nearest grocery retailer, disability, poor understanding of healthy behaviors and nutrition, industry marketing of unhealthy foods, and increased access to unhealthy foods [5]. Supermarkets remain the primary site where U.S. families purchase their food [6]. The average family makes 2.2 trips to the supermarket per week and obtains 75% of its food from grocery stores [7]. Delivered within the store, POP nutrition education combined with environmental manipulations (e.g. shelf-signs, cooking demonstrations), has shown promise in producing modest changes in healthful food purchasing practices, but remains understudied [8]. Recently introduced nutrition counseling provided in supermarket-based retail clinics [e.g. The Little Clinic (TLC) at Kroger’s supermarkets]

offers a promising opportunity to study individualized nutrition therapy in the POP environment. Validation of the effectiveness of these POP dietary services is needed.

In addition, new online technologies that might address barriers to healthy eating are expanding throughout the grocery retail industry. One of these is website/app-based ("online") food shopping (e.g. Amazon Fresh, Kroger's online store). This technology creates a new POP environment that could be enhanced with tools to sort grocery inventories for specific diets and/or identify healthier alternatives to a consumer's traditional selections. For example, foods not within the consumer's diet plan (e.g. high calorie) could be eliminated by a simple point-and-click option. Consumers could easily learn the selections most applicable to them (e.g. a "low calorie" option would be appropriate for weight reduction), resulting in a learning curve reduction compared to effort-intensive approaches such as reading food labels. In order to address meal preparation skills, dietary selections could also be linked to recipes and the ingredients required to prepare them. Finally, these online shopping technologies have the potential to reduce impulse in-store food purchases and facilitate supermarket shopping through grocery pickup and home delivery features.

Another opportunity exists for enhancement of POP interventions through use of electronic shopping data. These data are automatically collected by retailers for marketing and other uses. These data could be used to target consumers with coupons and other financial incentives to promote healthy food purchases. Each food item could also be electronically linked to nutrition data for the product, creating a measure of the nutritive quality of a consumer's grocery purchases. Purchasing data linked to nutrition data could be reported to customers, in-store dietitians, and other healthcare providers in user-friendly figures and metrics. These data would allow healthcare providers to individualize nutrition education, track progress over time, and reduce the burden of repeatedly collecting dietary intake information (both on providers and patients). To date, use of refined purchasing data metrics to guide dietary education has not undergone scientific study. We believe that either independently, or in combination, these interventions described above might provide scalable solutions to nutrition-related public health problems.

True research partnerships between the supermarket industry and academic institutions to study existing and novel dietary interventions have not previously existed. After an extensive effort by the UC investigator team and Kroger, a research partnership has been established to conduct clinical trials and observational research to better understand how to promote health and wellness in a sustainable, integrated healthcare-retail business model. The investigator team and Kroger leadership believe that scientific insights gained through this research partnership will help shape the healthcare and retail industries, both of which are undergoing disruptive change towards customer-focused, scalable solutions to

promote health and wellness. This trial is the first of this partnership.

3.PRELIMINARY STUDIES

None.

4.INVESTIGATOR EXPERIENCE

Co-Principal Investigator (PI) experience, expertise, and capabilities are described in detail in the following attachments (see attached NIH Biosketch Steen and NIH Biosketch Couch).

5.EXPERIMENTAL DESIGN & METHODS

A minimum of 250 adults will be randomized to the two study arms and control group (see *Inclusion and Exclusion Criteria* sections). The study design is illustrated in Figure 1.

Figure 1. SuperWIN Study Design

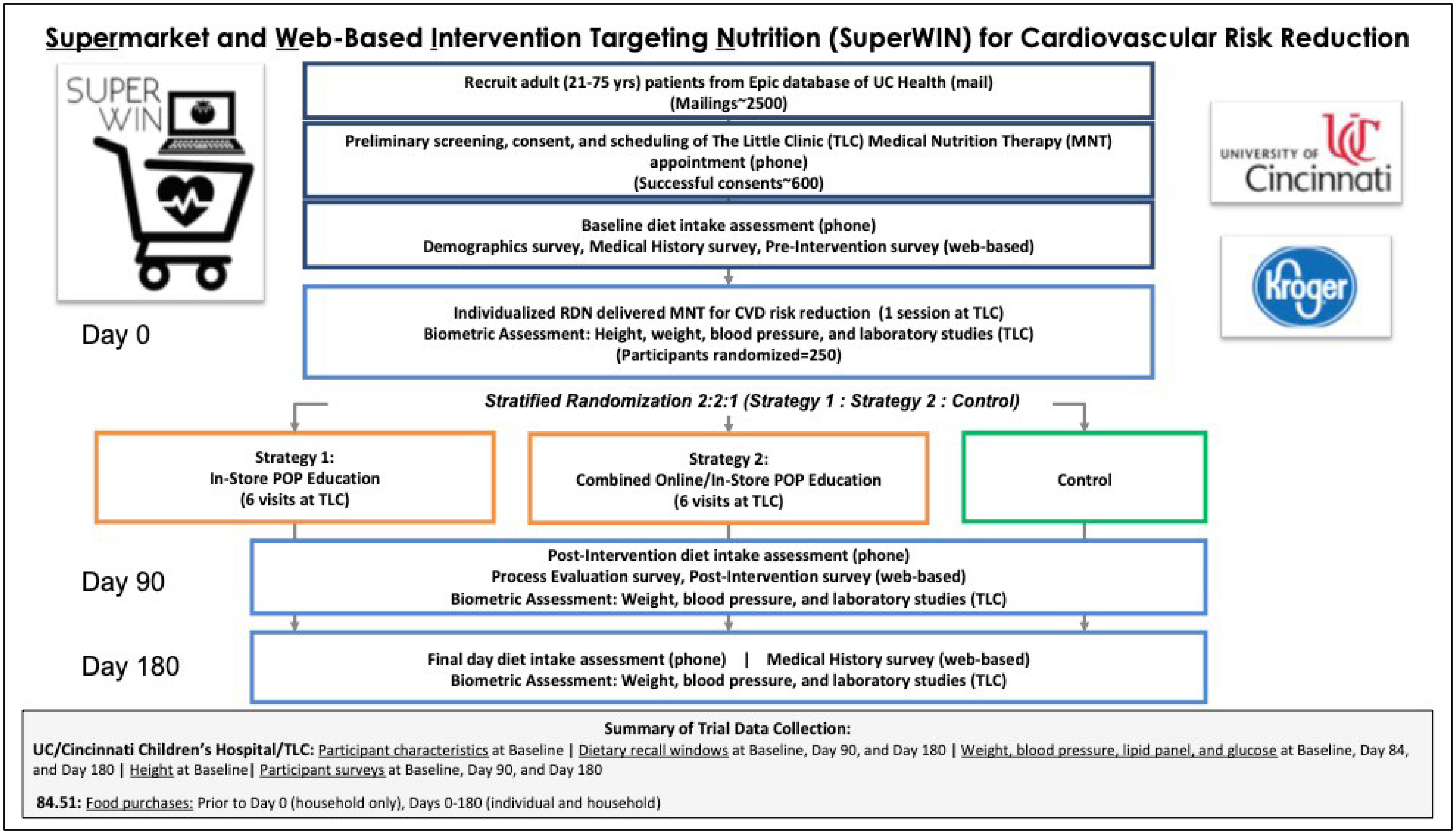

a. Inclusion Criteria

1) Age ≥21 to ≤75 years on the start date of the study (Day 0)
2) Speak, write, read English fluently
3) In-person outpatient visit with a UC Health primary care network (PCN) provider within the last 12 months
4) Major food planner of their household, which may include planning, purchasing, and/or preparing greater than 50% of the meals or foods consumed at home
5) Regular existing shopper at a study site Kroger (≥50% of grocery store food dollars spent at Kroger) with a Kroger Loyalty ID number
6) Willing to use a new individual Kroger Loyalty ID number for the duration of the study
7) Access to a home desktop or laptop computer with reliable internet access
8) Access to an email account that can be used for the duration of the study
9) Able to independently purchase and prepare food:
    a. Reliable transportation to participating Kroger store
    b. Any mobility issues that do not impact grocery shopping frequency or preparation of food at home
    c. Tools and equipment needed to prepare own food at home, such as a kitchen equipped with basic cooking tools (e.g. burner, pan, spatula, sink)
10) Presence of a cardiovascular risk factor(s) (at least one):
    a. Hypertension (defined by one of the following):
        i. Blood pressure measurement: systolic blood pressure (SBP) 130–189 mm Hg and/or diastolic blood pressure (DBP) 80–109 mm Hg (at Visit #1)
        ii. Current treatment with an oral medication prescribed by a physician for blood pressure lowering (defined by one of the following):
            1. Beta blockers
            2. Diuretics (e.g. thiazide or thiazide-like diuretics, loop diuretics, aldosterone antagonists, potassium-sparing diuretics)
            3. Angiotensin-converting enzyme inhibitors
            4. Angiotensin II receptor blockers
            5. Direct renin inhibitors (i.e. aliskiren)
            6. Calcium channel blockers (dihydropyridine and nondihydropyridine)
            7. Calcium alpha$_2$-agonist and other centrally acting drugs (e.g. clonidine)
            8. Direct vasodilators (e.g. hydralazine)

b. Body-mass index (BMI) measurement ≥30 kg/m^2 (at Visit #1)
c. Hypercholesterolemia (defined by one of the following):
    i. Low density lipoprotein cholesterol (LDL-C) 130-189 mg/dl (at Visit #1)
    ii. Current treatment with a medication prescribed by a physician for hypercholesterolemia (defined by one of the following):
        1. Statins
        2. Proprotein convertase subtilisin/kexin type 9 (PCSK9) inhibitors
        3. Absorption inhibitors (i.e. ezetimibe)
        4. Fibrates
        5. High-dose niacin (≥1 g per day)
        6. Bile acid sequestrants (e.g. cholestyramine)

b. <u>Exclusion Criteria</u>

1) Unwillingness or inability to modify current diet
2) Actively engaged in another dietary intervention or taking a weight-loss supplement
3) SBP ≥190 mm Hg or DBP ≥110 mm Hg (at Visit #1)
4) Low-density lipoprotein cholesterol (LDL-C) ≥190 mg/dl, triglycerides (TG) ≥600 mg/dl, or glucose ≥400 mg/dl (at Visit #1)
5) Evidence of prior diagnosis of severe chronic kidney disease (CKD) defined by electronic health record codes for dialysis, CKD Stage 4 or 5 and/or last estimated glomerular filtration rate (eGFR) ≤30 ml/min/1.73^2 [based on Modification of Diet in Renal Disease (MDRD) formula]
6) Active cancer other than non-melanoma skin cancers (i.e. basal cell carcinoma or squamous cell carcinoma)
7) Diagnosis of celiac disease, ulcerative colitis, or Crohn's disease
8) High risk for alcohol use disorder:
    a. ≥21 drinks a week (men)
    b. ≥14 drinks a week (women)
9) Women who are pregnant or plan to become pregnant within the next 6 months from the start of the study (Day 0)
10) Food allergies requiring a specialized diet, including allergies to dairy products, eggs, peanuts, tree nuts, soy, gluten-containing wheat and grains, fish, and shellfish
11) Previous visit at TLC for diet counseling

12) Frequent (≥1x/within the last 12 months) shopper using Kroger's online shopping platform (Kroger.com or the Kroger app) or unwillingness to use it throughout the study.
13) Participant lives greater than 20 miles from any participating Kroger study store
14) Women, Infants, and Children (WIC) beneficiaries in their household

c. <u>Recruitment Process</u>

Step 1: The PIs, the SuperWIN project manager, and the SuperWIN research coordinator(s) will be responsible for recruiting participants. Participants will be recruited with assistance from UC Health PCN providers. Patient panels of these providers will be pulled from Epic (Clarity database) to generate a list of those who meet broad study eligibility criteria and MyChart to send a message to potential patients (see attached HIPAA Partial Waiver for Recruitment).

Recruitment will be conducted using up to seven methods: 1) letter from study team; 2) follow-up postcard from study team; 3) flyers in PCP offices; 4) follow-up phone calls; 5) email messages including through MyChart, 6) text messages and 7) Kroger marketing.

Method 1: Patients will be mailed a recruitment packet, containing a letter from the study team and his/her provider (see attached Letter from Study Team) as well as a written informed consent form (ICF; see attached Informed Consent Form). The letter will provide a study-specific phone number for interested patients to call.

Method 2: For patients not responding to the initial letter, the study team will send them a postcard (see attached Postcard). This postcard contains general inclusion criteria, study procedures/assessments that would be required of the participants, and contact information if they are interested in being screened.

Method 3: In parallel to the initial study letter and postcard mailings, recruitment flyers and tear pads (see attached) will be available for PCP's to display in their respective offices. Both flyers have information about the study, as well as information regarding who to contact to be screened.

Method 4: If these methods of recruitment are still producing a small number of participants, the study team will call patients from Step 1 that have yet to respond to any of the other recruitment methods (Methods 1-3). These phone calls will simply ask if the participant is interested in being considered for the study. If the participant is interested, the verbal consent screening process will ensue (see telephone screening script). If the participant is not interested, the study team

will thank them for their time.

Method 5: In parallel with Method 4, an email will be sent to patients meeting the preliminary inclusion criteria including but not limited to MyChart and email addresses on file in Epic (Clarity database).

Method 6: In parallel with Method 5, the research team will send a text message to patients who have not responded to the other avenues of outreach.

Method 7: In parallel with Method 4, the research team will work with Kroger marketing to help recruit for the study. This could include, but is not limited to, setting up flyers inside the store asking interested shoppers to inquire about the study if they have a primary care physician through UC Health.

The intended study population is not specific to gender, racial or ethnic distribution. The present study will not recruit from groups considered to be vulnerable. Vulnerable populations may include, but are not limited to: children, minors, pregnant women, and the terminally ill [9, 10].

d. Study Methods and Procedures

Call #1 will screen interested UC Health patients by phone for eligibility. The screening performed at this visit will be conducted at least 30 days prior to the intended randomization date. On Call #1, screening will be performed by the SuperWIN study team (i.e. research coordinator or project manager), who have been trained by the Co-PIs. A screening script developed for this study will be utilized (see attached Telephone Screening Script). Those participants who provide verbal consent and meet the general eligibility criteria (see *Consent Form* section) will be considered study participants. Once oral consent is obtained and eligibility is confirmed, Call #1 will be used to schedule each participant's Visit #1 at the appropriate Kroger supermarket location (see *Settings and Facilities, Inclusion Criteria, and Exclusion Criteria* sections for more details on location assignment). Throughout the study, all of the participant's in-person study visits will take place at TLC in this Kroger supermarket. Each visit will be conducted by a TLC RDN assigned to this location.

At the end of Call #1, an email will be sent through REDCap to each consented participant containing online surveys to collect demographic, medical, and other pre-intervention data (see attached Demographics Survey, Medical History Survey, and Pre-Intervention Survey). The Pre-Intervention Survey will assess each participant's perceptions of food literacy, self-confidence, and frequency of behaviors related to shopping, cooking, and eating, as well as characteristics of the participant's household. Each participant will be informed to complete all surveys within 7 days of Call #1. If the surveys are still not completed at 7 days, the study team will call the participant to determine whether he/she is still

interested in study participation and to identify the barriers to survey completion (e.g. inadequate internet access). If he/she is felt to still be an appropriate study participant, the research team will guide the participant through the surveys. If the participant is not responsive or does not want to complete the surveys, Visit #1 will be cancelled. This email will also contain a handout with instructions to assist them with estimating portion sizes of foods/beverages during subsequent dietary intake recall assessments (see attached Food Amounts Booklet).

Table 1. Chronological Order of Study Contacts [days preceding the study start date are labelled with (-)]

Contact	Contact components	When would participant be expected to complete this contact?	How much participant time will be required?
**Study Contacts for All Participants**			
Call #1	• Obtain verbal consent for study (via Telephone Screening Script)<br>• Obtain contact information/preferences<br>• Screen participant for eligibility (via Telephone Screening Script)<br>• Schedule Visit #1	On or before Day -30	40 minutes
Email #1	• Demographic Survey<br>• Medical History Survey<br>• Pre-Intervention Survey<br>• Food Amounts Booklet	Within 7 days of Call #1	35 minutes
Calls #2-4	• 3 separate 24-hour dietary recalls	Days -30 to -2	45, 30, and 30 minutes
Visit #1	• Obtain written consent for study<br>• Biometric assessment (height, weight, waist and hip circumferences, blood pressure, blood lipid panel and glucose)<br>• 30-minute MNT session by RDN<br>• Final assessment of eligibility<br>• Visit #1 Checklist completion (via REDCap)<br>• Assign new Kroger Loyalty ID number<br>• Randomization to 1 of 3 study arms<br>• Orientation to randomized arm*<br>• Complete W-9 form	Day 0	90 minutes

	• Schedule all subsequent visits		
**Study Contacts following Visit #1 for Participants Randomized to POP Education Arms**			
Email #2	• Link to view educational videos for online shopping and apps (<u>only</u> for combined online/in-store POP arm)	Day 0	25 minutes
Visits #2-6	• Review of Snapshot[#]<br>• In-store and/or online POP education<br>• POP Checklist completion<br>• Review/confirm next visit date/time	Days 14, 28, 42, 56, and 70	60 minutes (plus up to 30 minutes for shopping)
Visit #7	• Review of Snapshot<br>• In-store and/or online POP education<br>• POP Checklist completion<br>• Biometric assessment (weight, waist and hip circumferences, blood pressure, blood lipid panel and glucose)<br>• Review/confirm next visit date/time	Day 84	75 minutes (plus up to 30 minutes for shopping)
Email #3	• Process Evaluation Survey<br>• Post-Intervention Survey	Day 84 to 91	35 minutes
Calls #5-7	• 3 separate 24-hour dietary recalls	Days 85 to 99	30 minutes each
Calls #8-10	• 3 separate 24-hour dietary recalls	Days 166 to 180	30 minutes each
Email #4	• Medical History Survey	Days 173 to 180	15 minutes
Visit #8	• Biometric assessment (weight, waist and hip circumferences, blood pressure, blood lipid panel and glucose)<br>• Consent for Re-contact after Day 180	Day 180	30 minutes
**Study Contacts following Visit #1 for Participants Randomized to the Control Arm**			
Visit #2	• Biometric assessment (weight, waist and hip circumferences, blood pressure, blood lipid panel and glucose)<br>• Review/confirm next visit date/time	Day 84	35 minutes

Email #2	• Process Evaluation Survey<br>• Post-Intervention Survey	Day 84 to 91	35 minutes
Calls #5-7	• 3 separate 24-hour dietary recalls	Days 85 to 99	30 minutes each
Calls #8-10	• 3 separate 24-hour dietary recalls	Days 166 to 180	30 minutes each
Email #3	• Medical History Survey	Days 173 to 180	15 minutes
Visit #3	• Biometric assessment (weight, waist and hip circumferences, blood pressure, blood lipid panel and glucose)<br>• Consent for Re-contact after Day 180	Day 180	30 minutes

TLC; The Little Clinic: RDN; registered dietitian nutritionist: POP; point-of-purchase: MNT; medical nutrition therapy.
All visits will be conducted within the same store to which the participant was randomized. All visits will be scheduled within a ± 3-day window. Participants will receive a reminder phone call prior to each study visit.
24-hr dietary recalls will be scheduled to collect data from 1 weekend day and 2 weekdays.
*For example, if participant is randomized to the combined online/in-store POP arm, the orientation will include educating the participant on Kroger.com (e.g. setup account).
#Snapshot refers to a collection of figures and tables highlighting purchase data metrics.

Calls #2-4 will consist of a set of 3 dietary intake recalls from a trained interviewer at the Bionutrition Core of the Schubert Research Clinic at Cincinnati Children's Hospital Medical Center (CCHMC). During each call, the interviewer will conduct a 24-hour dietary recall, collecting detailed dietary intake information specific to the participant from the previous day. The mean from all three dietary recalls will be used to calculate a baseline DASH score for each participant. These random calls aim to collect intake data for 1 weekend day and 2 weekdays. Another set of 3 dietary recalls will be conducted between Days 85-99 as well as between Days 166-180, using the same protocol.

Visit #1: The participant will meet the TLC RDN who will conduct all the visit tasks (Table 2). The RDN will begin by reviewing the SuperWIN study with the participant and obtaining a written consent. The RDN will then proceed to collect biometric data that includes height, weight, waist and hip circumferences, as well as blood pressure measurement. A blood lipid panel [total cholesterol (TC), LDL-C, high-density lipoprotein cholesterol (HDL-C), and TG levels] and blood glucose measurement will be obtained using a finger stick method. If study eligibility criteria are not met, the participant will be considered a screen-failure with this captured in REDCap. If eligibility criteria are met, the participant will receive a 30-minute, individualized, medical nutrition therapy (MNT) session. Visit #1 will introduce all participants to the key concepts of the DASH dietary pattern as well as address basic steps to implementing this dietary pattern, including shopping and cooking strategies. The data captured in the Demographic, Medical History, and Pre-Intervention Surveys

and dietary intake recalls (from Calls #2-4) will be available to the TLC RDNs via REDCap during Visit #1 and throughout the remainder of the study to individualize nutrition education.

At the end of the MNT session, if the participant is still felt to be eligible by the RDN (e.g. participant demonstrated interest in the DASH diet and engagement during the MNT session), the participant will then be randomized to 1 of 3 study arms. Participants will be informed of their randomization group by the RDN. Those participants randomized to the control arm will receive no further nutrition education throughout the study. Those participants randomized to one of the POP education arms will receive an additional 6 nutrition education visits (see *POP Education Visits* section). A brief orientation of the assigned arm will be provided to each participant.

Before the end of Visit #1, all randomized participants will receive a new, individual Kroger Loyalty ID card and number for the participant's sole use during the study. They will be instructed to have all other household members continue to use the household's pre-existing Kroger Loyalty ID card and number. At the end of Visit #1, all participants will be required to complete a W-9 form. This form is needed for participants to receive their incentives later in the study.

All randomized participants will receive the DASH Goal Tear Sheet and Goal Tracking Form (see attached) at Visit #1, in addition to other informational handouts (see attached Links to Handouts and Videos). All participants randomized to the combined online/in-store POP arm will be sent an email with a link to view educational videos on how to best shop online and use other tools to support healthier eating.

Table 2. Description of Visit #1 [Study Start Date (Date of Randomization)]

Visit Component	Specific Objectives and Content
General Study Introduction<br>(10 minutes)	• Discuss SuperWIN research program:<br>o Review aims of the study<br>o Review SuperWIN program expectations<br>o Explain concept of random assignment<br>• Review the ICF<br>• Obtain the participant's signature on the ICF
Biometric Assessment<br>(15 minutes)	• Measure height, weight, waist and hip circumference, blood pressure, blood lipid panel and blood glucose
Medical Nutrition Therapy<br>(30 minutes)	• Discuss DASH dietary pattern for CVD risk reduction:<br>o Review DASH food groups<br>o Explain the connection between DASH food groups and CVD risk reduction<br>• Review personal DASH dietary targets for CVD risk reduction:<br>o Review dietary intake data for the following DASH-recommended targets:<br>▪ Fruits<br>▪ Vegetables<br>▪ Low fat dairy<br>▪ Grains<br>▪ Proteins<br>▪ Nuts/Seeds/Beans<br>▪ Fats & Oils<br>▪ Sweets<br>▪ Sodium<br>▪ Calories<br>o Negotiate personal targets for change<br>• Explain DASH goals and relate to helping make dietary changes:<br>o Use personal target worksheet to set a DASH goal<br>o Provide handouts of strategies for eating a DASH-type diet and discuss these and other strategies with participant.<br>o Provide and review the DASH Goal Tear Sheet to establish personal targets and an action plan

Randomization (15 minutes)	• Explain goal of self-monitoring:<br>o Discuss the valued of goal tracking for meeting dietary goals<br>o Provide instructions for completing the Goal Tracking Form<br>• Confirm study eligibility and randomize (if appropriate)<br>• Review randomization assignment<br>• Orientation to randomized arm
Wrap-up (20 minutes)	• Complete Visit #1 Checklist in REDCap<br>• Assign new Kroger Loyalty ID number<br>• Complete W-9 Form<br>• Schedule all future TLC visits

CVD; cardiovascular disease: ICF; informed consent form: TLC; The Little Clinic.

<u>POP Education Visits:</u> Major themes, learning outcomes, and skill-building exercises will be consistent across modules covered in both POP education arms. Each participant's 6 POP education visits will be delivered at TLC by a RDN. The in-store POP education arm participants will spend the majority of each visit being educated within the aisles of the store.

In contrast, the combined online/in-store POP education arm participants will spend time being educated both in the aisles of the store as well as on Kroger's web-based shopping platform (Kroger.com). Emphasis will be placed on shopping using Kroger.com with either grocery pickup or home delivery. This arm will also be enhanced with the introduction and use of other online/app-based tools, including OptUp, Yummly, and The Grocery Runners. At Day 0, this arm will be provided short educational videos on topics such as shopping online, budget/meal planning online, and finding DASH-friendly recipes online. These are to be viewed at home and again, if needed, through REDCap during POP visits (see attached Links to Handouts and Videos). Time spent by the RDNs on each technological tool will depend upon the needs of the participant.

POP education will be individualized to each participant using baseline dietary intake recall data, individual food purchasing data metrics (automatically collected via a newly-issued Kroger Loyalty ID number), as well as review of updated the DASH Goal Tear Sheet and Goal Tracking Form (provided at Visit #1 and each subsequent POP visit). Purchasing data metrics will be updated and provided at each POP visit. The data will be displayed via easy-to-interpret figures and tables, referred to as a "Snapshot". The dietitian and participant will review an updated Snapshot at each POP visit. These data will serve to provide a better understanding of the overall purchasing habits of the participant, as well as

to identify specific behaviors that should be promoted or modified.

Learning objectives within each arm will be framed by the POP education strategy. Content covered within each visit will be recorded by the RDN via the POP Checklist (see attached) in REDCap. In addition to the DASH Goal Tear Sheet and Goal Tracking Form, informational handouts will be provided to the dietitians to distribute to participants at their discretion. Each POP visit will cover 1 module. Figure 2 and Table 3 describes the topics and flow of the 6 modules covered in each POP education arm. The POP education sessions are grounded in the social cognitive theory. Theory elements that are covered in the different sessions are also highlighted in Table 3.

Figure 2. POP Education Module Topics

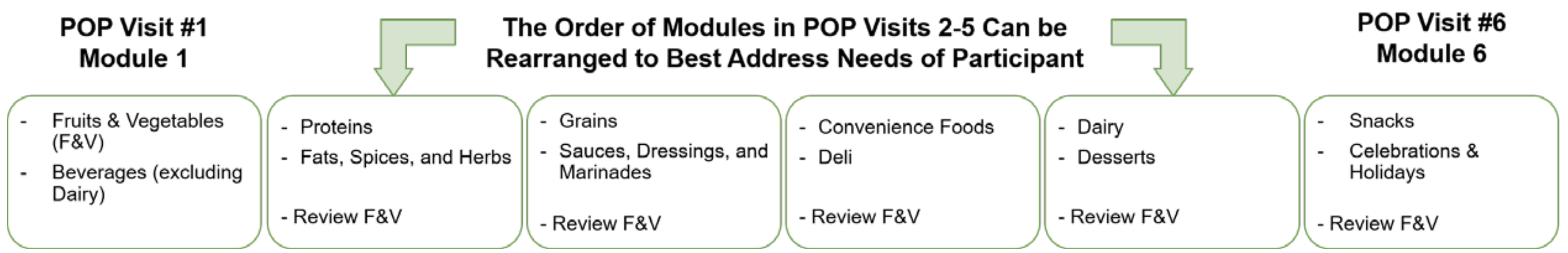

Table 3. POP Education Module Flow Summary

	Section	Time	Components	ADIME
The Little Clinic	Review	10 minutes	▪ Review the DASH Goal Tear Sheet and Goal Tracking Forms† ▪ Review Snapshot* ▪ Snapshot-guided discussion: - Feedback from previous goals and action plan - Strengths related to food topic of the week - Weaknesses related to food topic of the week - Barriers to healthy eating ▪ Set DASH goals	Assessmen t<br>Diagnosis

Appropriate POP Setting	Education	40 minutes	▪ Orientation to food topic of the week<br>▪ Address learning objectives using at least 2 suggested strategies in each of the following areas of food literacy: selecting DASH compliant foods (e.g. trying something new, reading labels, making substitutions), preparing DASH compliant foods (e.g. recipe identification and modification, meal planning), and discussing the health benefits of DASH.<br>▪ Orientation to POP education strategy-specific tools	Intervention
	Wrap-up	10 minutes	▪ Create action plan for DASH goals<br>▪ Distribute relevant handouts<br>▪ Planning for next visit:<br>- Review/confirm next visit date/time<br>- Reminder to complete/return the DASH Goal Tear Sheet and Goal Tracking Forms†	Monitoring Evaluation
	Shopping	30 minutes	▪ Participant shops independently	

ADIME; Assessment, Diagnosis, Intervention, Monitoring, and Evaluation: DASH; Dietary Approaches to Stop Hypertension: POP; point-of-purchase.
All visits will be conducted within the same store to which the participant was randomized. Participants will receive a reminder phone call prior to each study visit. All visits will be scheduled within a ± 3-day window.
24-hr dietary recalls will be scheduled to collect data from 1 weekend day and 2 weekdays.
*Snapshot refers to a collection of figures and tables highlighting purchase data metrics.
†Handouts to establish targets, goals and action plans, and self-monitoring.

All randomized participants will repeat weight, waist and hip circumference, blood pressure, blood lipid panel, and blood glucose measurements at Day 84. For those randomized to the POP education arms, this will occur at Visit #7.

On Day 84, all randomized participants will be sent an email with a Post-Intervention Survey and Process Evaluation Survey (attached) to be completed within 1 week. Participants will receive a reminder email if these have not completed within 1 week of receiving them. Up to 3 reminder phone calls will be made if these have not completed within 2 weeks of receiving them. After 2 weeks, if a participant still has not filled out the surveys, the study team will call the participant to complete the surveys as soon as possible.

On Day 173, all randomized participants will be sent an email with a subset of the Medical History Survey questions. Participants who were randomized into a POP education arm will also be sent an email 1-3 weeks prior to day 180 inviting them to participate in a focus group. Please see the Focus Group Protocol for more information on the focus group methodology. On Day 180 (the final TLC visit), weight, waist and hip circumference, blood pressure, blood lipid panel, and blood glucose measurements will be repeated in all randomized participants. If a participant has not completed the Medical History Survey questions by Day 180, the participant will be asked to complete them at the visit. Focus groups will take place following Day 180. All randomized participants will be asked for consent to re-contact after Day 180.

Table 4. Schedule of SuperWIN Procedures

	**Days**											
	**≥ -30**	**-30 to -2**	**0**	**14**	**28**	**42**	**56**	**70**	**84**	**85-99**	**166-180**	**180**
Contact Method												
Phone	X	X								X	X	
Email		X	(X)						X		X	
Visits (Control Arm)			X						X			X
Visits (POP Education Arms)			X	X	X	X	X	X	X			X
**Procedures to be Completed (All Participants)**												
Obtain Verbal Consent	X											
Screening for Eligibility	X											
Schedule Visit #1	X											
Review Food Amounts Booklet		X										
Complete Demographic Survey		X										
Complete Medical History Survey		X									X	
Complete Pre-Intervention Survey		X										
Complete 24-hour Dietary Intake Recalls (3 per time-point)		X								X	X	
Obtain Written Consent			X									
Measure Height			X									
Measure Weight			X						X			X
Measure Waist and Hip Circumferences			X						X			X

Measure Blood Pressure			X						X			X
Measure Blood Lipid Panel			X						X			X
Measure Blood Glucose			X						X			X
Complete 30-minute MNT (including dietary intake recall review)			X									
Complete Final Assessment of Eligibility			X									
Complete Visit #1 Checklist			X									
Assign new Kroger Loyalty ID number			X									
Randomize to 1 of 3 Arms			X									
Orient to Randomized Arm			X									
Complete Post-Intervention Survey										X		
Complete Process Evaluation Survey										X		
Consent for Re-contact												X
**Procedures to be Completed (POP Education Arms Only)**												
Review Videos			(X)									
Complete POP Education Module (including Snapshot review)				X	X	X	X	X	X			
Complete POP Checklist				X	X	X	X	X	X			

TLC; Kroger's The Little Clinic: RDN; registered dietitian nutritionist: POP; point-of-purchase: MNT; medical nutrition therapy.
All visits will be conducted within the same store to which the participant was randomized. Review and confirmation of the date/time of the following appointment is confirmed at each visit. Participants will receive a reminder phone call prior to each study visit.
All visits will be scheduled within a ± 3-day window.
24-hr dietary recalls will be scheduled to collect data from 1 weekend day and 2 weekdays.
(X) email with link to videos will only be sent to the combined online/in-store POP arm.

e. <u>Data Collection Tools</u>

After providing oral consent, each participant will be emailed electronic surveys through REDCap to collect: demographic, medical, and other pre-intervention information (see attached Demographic Survey, Medical History Survey, and Pre-Intervention Survey). Surveys are to be completed within 7 days. At Day 84, all participants will be emailed the Process Evaluation and Post-Intervention Surveys. These will assess participants' satisfaction and experience with the study interventions as well as changes in perceived food literacy concepts (see attached Process Evaluation Survey and Post-Intervention Survey). At Day 173, all participants will be emailed a subset of the Medical History Survey questions to

capture any changes in medical status that occurred during the time of the study.

For each participant, trained interviewers at CCHMC Bionutrition Core will use the Nutrition Data System for Research (NDSR) computer program to collect a total of 9 x 24-hour dietary intake recalls by phone. NDSR employs the USDA automated multiple-pass method in the collection of the dietary recall information. This method prompts participants to recall general information about their intake on the first pass, more specific details about these foods and amounts on the second pass, with a final review of descriptions and amounts on the third pass. This dietary information will be used to calculate a DASH score [11].

Kroger's existing practice is to link all household member purchases together under one Kroger Loyalty number. As the study inclusion criteria require each participant to be an existing Kroger shopper, each participant should be linked to this Kroger Loyalty number before the start of recruitment. During Visit #1, each participant will be given a *new,* individual Kroger Loyalty number. This new number is for the participant's sole usage for the duration of the study (Day 0 to Day 180); all other household members will continue to use the pre-existing number during this period. This study feature will allow separation of the participant's purchases from those of other household members throughout the study. In addition, household purchase data will be pulled into the study database by 84.51°, using patient identifiable information (e.g. name, address) submitted via password-protected communications, from 365 days prior to Day 0. Purchasing data captured from the new Kroger Loyalty number will be used to prepare metrics for the RDNs to review with each participant assigned to the POP education arms during Visits #2-7. These data will be displayed on study-issued tablets via a web-based, interactive, graphic "Snapshot." The intention of the Snapshot is to individualize POP education visits by the use of electronically, automatically collected data. Purchasing data will be analyzed as a secondary study outcome.

The RDN will measure each participant's height, weight, waist and hip circumferences, blood pressure, blood lipid panel, and blood glucose via a finger stick at Visit #1. A blood lipid panel consists of TC, HDL-C, LDL-C and TG. All measurements, except height, will be repeated at Day 84 and Day 180. These data will be entered into REDCap, an electronic case report form (eCRF) platform.

All visits will be guided and documented by the RDN using a Visit #1 Checklist (Table 2) and a POP Checklist (see attached). This information will be entered into REDCap.

f. <u>Statistical Analysis</u>

A minimum of 250 adults will be randomized into the study. Approximately 100, 100, and 50 participants will be randomized to the in-store POP education arm, the combination online/in-store POP education arm, and the control arm, respectively (see section *Data Analysis and Data Monitoring* for power calculations and testing plan).

NDSR food data will be used to calculate overall diet quality using the DASH score. The DASH score is a modification of the DASH index developed by Gunther et al. [11]. DASH score is a continuous 90-point measure with higher scores equating to higher DASH diet adherence.

Randomization will use stratification to balance important baseline covariates among study groups. These covariates will be age, sex, and household size.

Data will initially be assessed for their distribution (e.g. normality) and the level of missing values. Descriptive statistics will be calculated for each arm. The primary endpoint will be the change from baseline to Day 90 in the DASH score (as assessed by dietary intake recalls). Using hierarchical testing, up to 2 tests will be performed:

1) First test: Any POP education arm vs. control arm
2) Second test: In-store POP education arm vs. combined online/in-store POP education arm

An intention-to-treat (ITT) analysis will be used for both tests. The “last observation carried forward” approach will be used for missing data. Ascertainment of DASH score at Day 90 for each participant will require completion of at least 1 of 3 dietary intake recalls. Sensitivity analyses may be performed using a “complete case” approach or multiple imputation techniques.

The primary comparisons will use an analysis of covariance (ANCOVA) model. This multivariable model, controlling for the baseline covariates, will have DASH score as the dependent variable with treatment arm as the independent variable. As part of this process, the normality of distribution of DASH scores at both baseline and Day 90 will be assessed. If necessary, transformations (e.g. log) will be used to satisfy the assumptions of the model. The primary treatment group comparisons will be performed using appropriate contrasts of the randomization arm estimates. Statistical testing will only be performed for the second test if $p < 0.05$ is met for the first test (i.e. hierarchical testing). This will preserve an alpha-error of 0.05.

Using 100 participants for each intervention arm (total = 200) and 50 participants for the control arm, we have 93% power to detect a 5-point difference in change from baseline in the DASH score at Day 90 between any POP education arm and the control arm. This sample size will also provide 97% power to detect a 5-point difference between the in-store and combined online/in-store POP education arms. Power estimates were done at two-sided alpha = 0.05 and assuming equal standard deviations of 9 for the primary endpoint.

All analyses will be conducted according to the statistical analysis plan (SAP). Exploratory analyses will be done for important subgroups (e.g. sex, age, race). The following table provides examples of power to detect a 5-point difference in change from baseline to Day 90 in the DASH score between the in-store and combined online/in-store POP arms for subgroups. Numerous exploratory analyses will be performed for secondary outcomes, such as dietary quality measured by the Healthy Eating Index-2015 (HEI-2015).

Table 5. Statistical Power for Exploratory Subgroup Analyses

Subgroup sample size in each treatment group	Power to detect 5-point difference between in store and combination online/in-store POP arms*
52, 52	80%
60, 40	77%
40, 40	69%
50, 30	66%
30, 30	56%

*Note that secondary analyses should be considered exploratory as the alpha error will not be contained at 0.05.

g. <u>Data Storage and Confidentiality</u>

Upon oral consent, each participant will receive a unique, study ID number. The study ID number will be generated in the following format: three-letter identifier (e.g. LEB for Lebanon) followed by the participant number (0001, 0002, 0003, etc.). All subsequent participant research materials (including surveys and data collection tools) will only include participant ID numbers. Identifiable participant information will be stored in a screening log maintained by the study team. Participant identification will be limited to the SuperWIN research coordinators, SuperWIN project manager, CCHMC Bionutrition Core staff, Grocery Runners staff, study store TLC staff, and 84.51 (Kroger's analytics company, 84.51°).

Except for 84.51°, these individuals will have direct contact with participants. At study visits, TLC RDNs will be able to also review each participant's collected data, including the baseline Demographic, Pre-Intervention, and Medical History Survey findings, baseline diet intake data, baseline biometric data, and updated purchase data.

The CCHMC Data Management and Analysis Center (DMAC) staff will ensure the safe transfer and storage of participant data (including to and from 84.51°). Urgent (e.g. participant reports of syncope due to hypoglycemia) and nonurgent (e.g. baseline LDL-C 240 mg/dL) safety issues will result in notification of the participant's primary care provider (section 6b - *Safety Considerations*).

During the course of the study, information collected over the phone or electronically will be made available to key research personnel through a secure, file transfer protocol site. This includes scheduled visit dates and times, survey data, biometric data, dietary intake data, and purchase data. Diet intake and participant purchase history data will be stored on a secure server. It will then be merged together using SAS with data from REDCap for reporting and analyses.

Upon conclusion of the study, this information will be de-identified except for participant ID and Kroger Loyalty ID numbers. Any and all paper copies containing participant information (created in the event of computer or technical issues) will be promptly entered and stored in a locked file cabinet by the research coordinator or project manager. All electronic data will be stored on a secure server to be kept indefinitely by the investigators. Data and patient identifiers (e.g. name, home street address, birthdate, telephone numbers, email address, electronic health record number, dates of study procedures) will be deleted from staff computers and the recycle bin emptied. Hard-copies of completed surveys and other data collection tools will be kept in a locked file cabinet for 5 years after the conclusion of the study, after which they will be shredded.

h. <u>Setting and Facilities</u>

Each participant will be assigned to the Kroger study store at which the participant conducts his/her regular grocery shopping. Each study visit will be conducted at TLC in each store, across the Cincinnati and Northern Kentucky regions.

TLC is an independent medical clinic located within Kroger supermarkets, staffed by board-certified nurse practitioners, physician assistants, and registered dietitian nutritionists qualified to diagnose and treat minor medical issues. All medical assessments, including height, weight, waist and hip circumferences, blood pressure, blood lipid panel, and blood glucose

measurements will be made by a RDN or nurse in a private patient consultation room within TLC.

Dietary intake recall data will be collected via phone call from trained interviewers at the Bionutrition Core of the Schubert Research Clinic at CCHMC.

i. Estimated Period of Time

The entire research project will take an estimated 2.5 years to complete after UC IRB approval.

6.RISK/BENEFIT ASSESSMENT

a. Level of Risk

Table 6. Intervention Risks

Risk or Discomfort	Level
Physical – finger stick blood lipid panel and glucose measurement<br><br>Use of a finger stick is less invasive and takes less time than laboratory tests done at a physician's office. A finger stick requires significantly less blood and uses a smaller needle, than a typical blood draw done at a physician's office.	Minimal
Physical – blood pressure measurement<br><br>The blood pressure cuff may cause some discomfort while constricting to measure blood pressure. This discomfort is no greater than that experienced at a physician's office when measuring blood pressure.	Minimal
Emotional – nutrition counseling<br><br>Participant may feel some embarrassment or frustration when discussion their eating habits, food budget, or food purchasing habits with a registered dietitian.	Minimal

b. **Safety Considerations**

All participants will undergo biometric assessments (height, weight, waist and hip circumferences, blood pressure, blood lipid panel, and blood glucose) at Day 0. All assessments, except height, will be repeated at Day 84 and Day 180. If a study participant exhibits abnormal biometric values or complains about a new and/or an exacerbation of pre-existing symptoms, their PCN provider will be contacted by the study team. Any contacts/referrals made for safety considerations will be captured in a contact log. The following are examples that would warrant immediate PCN provider contact: SBP ≥220 mm Hg, SBP ≤80 mm Hg, DBP ≥130 mm Hg, blood glucose ≤60 mg/dl or ≤80 mg/dl with hypoglycemic symptoms, new-onset chest pain, severe fatigue or weakness, new-onset lightheadedness/dizziness or syncope, new-onset or persistent nausea, acute visual changes, worsening depression, or new-onset hypertension. In case of an emergency (as determined by the assessor), 911 would be called.

PCN providers will be given the names of their patients who have been randomized into the trial.

c. **Direct Benefit to Participants**

All participants will benefit from nutrition education, both from the individualized nutrition counseling as well as the provided education materials. Participants will also benefit from receiving 3 biometric assessments at TLC. Participants will be informed of their biometric assessment results (see Biometric Assessment Form) and may share this information with their providers, if they choose to do so. All of the above benefits will be provided at no cost to the participant.

The risk or discomfort in this study is no greater than that a participant would encounter at a medical office visit or nutrition counseling session.

7.PAYMENT

Participants will receive 2 incentives in separate installments. After the completion of the second set of 3 dietary recalls following Day 84, he/she will be mailed a $25 pre-paid debit Greenphire clincard. After the completion of the third set of 3 dietary recalls by Day 180, each participant will receive another $25 uploaded to his/her clincard. Participants who do not complete all 3 recalls at each time-point will not receive any monetary incentives. Clincards in the amounts of $25 and $25 are an appropriate, convenient, and non-coercive form of payment.

8.SUBJECT COSTS

As part of the nutrition education, participants will be encouraged to change food purchasing and eating habits to better align with a dietary pattern conducive to healthy living and decreased cardiovascular risk. Participants will be responsible for the cost of purchasing their own groceries. Participants will also be responsible for any costs associated with transportation to and from their selected Kroger study site.

Participants randomized to the combined online/in-store POP education arm will be encouraged to shop through Kroger's online shopping platform using their personal electronic devices and Internet connection. They will be provided with home grocery delivery services through The Grocery Runners, an independent home delivery company that provides services in Dayton, Cincinnati, and Northern Kentucky. The costs of shopping online will be minimized. The use of The Grocery Runners services will be provided at no cost to the participant.

9.CONSENT FORM

a. Consent Process

The consent process is broken down into four parts: 1) review of the recruitment packet; 2) verbal consent; 3) written consent; and 4) focus group consent.

A recruitment packet will be sent to potentially eligible UC Health PCN patients (see HIPAA Waiver for Recruitment). The packet will contain 1) the Letter from Study Team (see attached) and 2) the ICF (see attached). The ICF will be provided so participants can review it and discuss their questions at Visit #1 with the RDNs. Patients who receive the recruitment packet will be encouraged to call the study team if they are interested. The contact information for the study team is listed on the letter.

All interested participants who call the study team will first undergo a verbal consent process (see Telephone Screening Script). All individuals who are recruited through Methods 2-5 (postcard, PCP flyer, phone call, or through Kroger marketing) will also undergo a verbal consent process. During this process, the study manager or study coordinator will review the Telephone Screening Script with the participant. The study team member will prompt the patient to ask any

questions he/she may have in between each section of the script. Upon review of the verbal consent process, the research team member will ask for verbal consent to participate in the study. The research team member will document whether oral consent was obtained in REDCap. The script will obtain verbal consent for study procedures completed before Visit #1. If the participant provides verbal consent, the participant will be assigned a participant ID number. If the participant does not provide verbal consent, they will be considered a prescreen-failure and this data will be captured in the form of a screening log. Once the participant provides verbal consent, he/she will be screened for eligibility. The study team will continue to review general eligibility with the participant and, if he/she remains eligible, Visit #1 will be scheduled at the conclusion of the phone call.

At Visit #1, the participant will be given time to review the ICF with the RDN. The RDN will review the key points of the ICF, including the study purpose, participant involvement, risks, benefits, confidentiality, cost, payment, and rights. The RDN will prompt the patient to ask any questions he/she may have in between each section of the ICF. If the participant declines to participate at Visit #1, or if eligibility criteria are not met, he/she will be considered a screen-failure, with this captured in REDCap. If the participant gives written consent to participate in the rest of the study, the RDN will conduct the remainder of the Visit #1 procedures. The participant will be given a copy of the ICF. The original ICF will be stored at TLC in a secured location.

The participant will be informed that choosing to participate or not participate will not affect his/her relationship (including purchases) with Kroger or their affiliates, nor any medical care he/she may receive from TLC or UC Health. Each participant will be given the option for re-contact at Day 180.

For the focus groups, participants who complete SuperWIN as part of an intervention group will review the focus group informed consent with the dietitian at the Day 180 visit. If the participant agrees to attend the focus group, the dietitian will give them the purchasing snapshot questionnaire to be completed at home. All completed focus group informed consents will be collected by the study team and will be kept in a study binder. Further details surrounding the focus groups can be found in the focus group protocol.

b. <u>Consent Form</u>

See attached Telephone Screening Script, Informed Consent Form, Focus Group Informed Consent Form, and Day 180-Re-contact Sheet.

10.LITERATURE CITED[i] -

1. Moore, J.X., N. Chaudhary, and T. Akinyemiju, *Metabolic Syndrome Prevalence by Race/Ethnicity and Sex in the United States, National Health and Nutrition Examination Survey, 1988-2012.* Prev Chronic Dis, 2017. **14**: p. E24.
2. Committee, D.G.A., *Scientific report of the 2015 dietary guidelines advisory committee: Advisory report to the secretary of health and human services and the secretary of agriculture.* 2015, U.S. Department of Agriculture: U.S. Department of Agriculture, Agriculture Research Service. p. 436
3. Finkelstein, E.A., et al., *Annual medical spending attributable to obesity: payer-and service-specific estimates.* Health Aff (Millwood), 2009. **28**(5): p. w822-31.
4. Desroches, S., et al., *Interventions to enhance adherence to dietary advice for preventing and managing chronic diseases in adults.* Cochrane Database Syst Rev, 2013(2): p. CD008722.
5. Smith, L.P., S.W. Ng, and B.M. Popkin, *Trends in US home food preparation and consumption: analysis of national nutrition surveys and time use studies from 1965-1966 to 2007-2008.* Nutr J, 2013. **12**: p. 45.
6. Corporation, F.M.I.O.R., *Trends in the United States:consumer attitudes & the supermarket*. 1994, Food Marketing Institute: Research Dept., Food Marketing Institute.
7. Nielsen, S.J., A.M. Siega-Riz, and B.M. Popkin, *Trends in food locations and sources among adolescents and young adults.* Prev Med, 2002. **35**(2): p. 107-13.
8. Escaron, A.L., et al., *Supermarket and grocery store-based interventions to promote healthful food choices and eating practices: a systematic review.* Prev Chronic Dis, 2013. **10**: p. E50.
9. Assembly, W.M.A.G., *WMA DECLARATION OF HELSINKI – ETHICAL PRINCIPLES FOR MEDICAL RESEARCH INVOLVING HUMAN SUBJECTS*. 2018: World Medical Association. p. 4.
10. Shivayogi, P., *Vulnerable population and methods for their safeguard.* Perspect Clin Res, 2013. **4**(1): p. 53-7.
11. Gunther, A.L., et al., *Association between the dietary approaches to hypertension diet and hypertension in youth with diabetes mellitus.* Hypertension, 2009. **53**(1): p. 6-12.

11. ADDITIONAL DOCUMENTATION

1) NIH Biosketch Steen
2) NIH Biosketch Couch
3) HIPAA Partial Waiver for Recruitment
4) Letter from Study Team V1.0
5) Informed Consent V1.0
6) Postcard V1.0
7) Recruitment Ad Flyer V2.0
8) Recruitment Ad Tear Pad V2.0
9) Telephone Screening Script V1.0
10) Demographic Survey V1.0
11) Medical History Survey V1.0
12) Pre- and Post-Intervention Survey V1.0
13) Food Amounts Booklet V1.0
14) Links to Handouts and Videos
15) POP Checklist V1.0
16) DASH Goal Tear Sheet V1.0
17) Goal Tracking Form V1.0
18) Biometric Assessment Form V1.0
19) Process Evaluation Survey V1.0
20) Request for Waiver of Consent Documentation
21) Request for Waiver of Consent Process
22) Focus Group Protocol
23) Focus Group Informed Consent Form
24) Focus Group Information Sheet
25) Focus Group Questions
26) Purchasing Snapshot Questionnaire
27) Day 180 Re-contact Sheet

Supplementary Note 2: Statistical Analysis Plan

Statistical Analysis Plan

CCHMC Division of Biostatistics and Epidemiology (DBE)

Supermarket and Web-based Intervention targeting Nutrition (SuperWIN) for cardiovascular risk reduction

Principal Investigators: Dylan Steen, MD, MS, Sarah Couch, PhD, RDN
Statisticians: Eileen King, PhD, Matthew Fenchel, MS

Raw Data Check

It will involve all variables and all time-points. The raw data check will consist of the following:

Continuous Variables	N, N-Missing, Min, Median, Max
Categorical Variables	N, N-Missing, Count, Percent

The purpose of the raw data check is to examine data for outliers, missingness, and distributional properties. These will be described and (if necessary) adjusted for in the analyses. Histograms and bar charts may be produced.

Overall Analysis Approach

Descriptive Statistics. Descriptive statistics will consist of mean and standard deviation (SD) and median and interquartile range (IQR) for variables. Counts and percentages will be used for categorical variables. Unless otherwise determined, descriptive statistics will be produced for all variables and all time-points.

Distributions/Transformations. Residuals from models will be examined for primary and key secondary outcomes for distributional properties, to ensure model assumptions (normality and independence of residuals) are met. If they are not met, data transformation or other analysis methods (e.g. nonparametric) will be used.

Treatment Groups. Subjects were randomly assigned to one of three treatment groups: 1) Control; 2) In-store Point-of-Purchase Education (POP1); and 3) Combined Online and In-store Point-of-Purchase Education (POP2). The designation POP1/POP2 will be used to denote the combining of data from POP1 with POP 2.

DocuSign Envelope ID: 0D28DCB2-96A8-4360-9427-C8728A1D9207

Primary Outcome and Comparison: The primary outcome is the DASH score. The primary comparison of interest is the change in DASH score between baseline and the 3-month follow-up.

Key Secondary Outcomes: The main secondary outcomes are BMI, SBP, DBP, non-HDL cholesterol, total cholesterol and triglycerides.

Modeling. The primary comparisons will use a mixed model for repeated measures. This multivariable model, controlling for covariates, will have DASH score as the dependent variable with treatment as the independent variable. The primary treatment group comparisons will be performed using appropriate contrasts of the treatment arm estimates.

Significance Level (α) and Tests. For the primary and key secondary endpoints, hierarchical testing will be used, first comparing Control vs. POP1/POP2. If $p < 0.05$ for that test, then the two intervention groups will be compared (POP1 vs. POP2). This will preserve an alpha-error of 0.05. For all other endpoints, testing will be done at the $\alpha = 0.05$ significance level, with no multiple comparison adjustments. All tests, unless otherwise noted, will be two-sided.

COVID-19 Impact Analysis. Due to the COVID-19 pandemic occurring during the course of the SuperWIN, we have a COVID-19 impact analysis following regulatory guidance, defined below.

Software. Unless otherwise specified, SAS® 9.4 TS1M5 (SAS, Inc., Cary, NC) software will be used for all analyses.

Study Timepoints

Baseline: Data collected at, or before, the completion of Visit 1 (on Day 0).

Follow-up: Data collected after the completion of Visit 1 (on Day 0).

3 Month ("Day 90"): Dietary recalls are collected at this timepoint. These recalls are under "Day 85-99 Telephone Dietary Recall" in REDCAP and occur within the range of Days 1 to <166 from randomization.

6 Month ("Day 180"): Dietary recalls are collected at this timepoint. These recalls are under "Day 166-180 Telephone Dietary Recall" in REDCAP and occur within the range of Day 160 from randomization until the end of the study on 9/3/2021.

FIGURE: CONSORT DIAGRAM

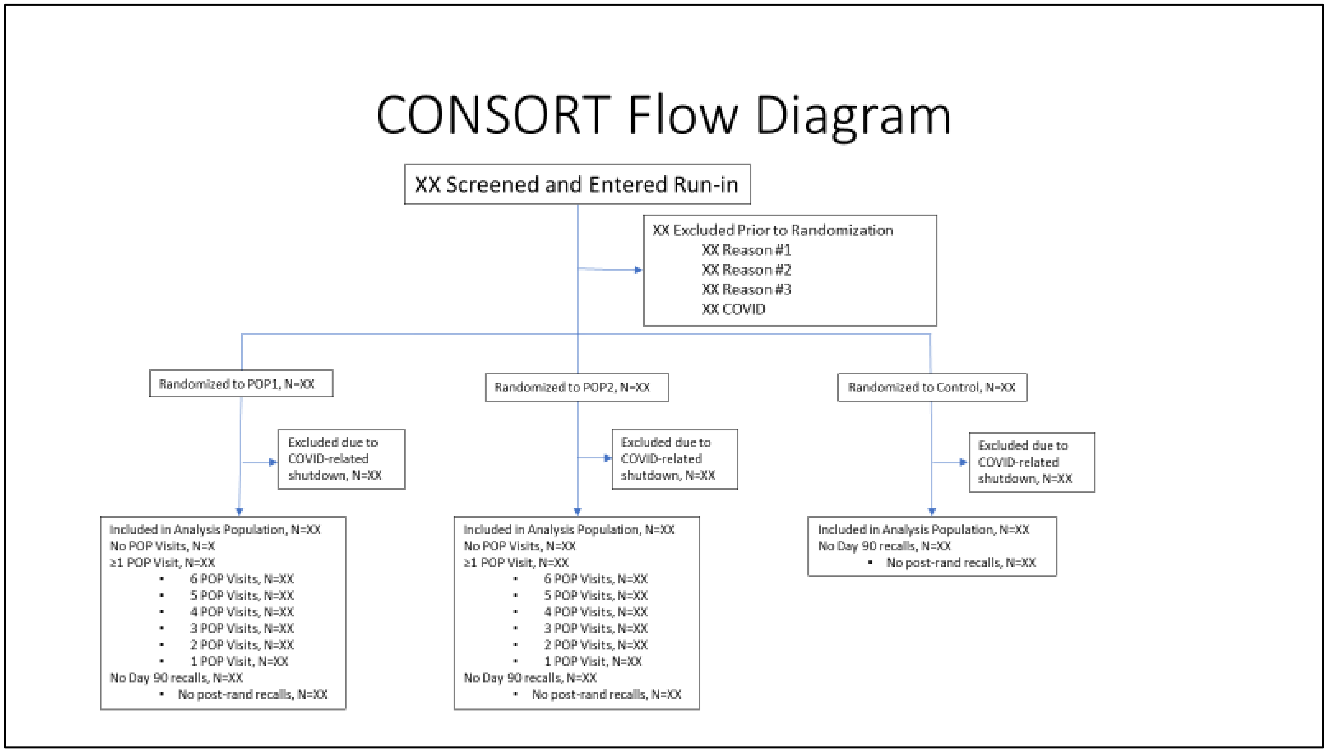

POP1: In-Store Only Arm. POP2: In-Store and Online Arm.

Programming Specifications for CONSORT Flow Diagram

1) Screened and Entered Run-In: Count all participants whose answer to the question "Is this participant eligible to have their visit #1 scheduled?" is one of the following:
   a. "Yes"
   b. "No" and the reason given for "Why" is "Other", with a free-text reason listed under "Explain why the participant is not eligible." To be included in this count, the reason needs to indicate that the patient got into the run-in but then was removed (e.g. "Did not complete baseline surveys").

2) Excluded at the Randomization Visit ("Visit 1"): For those participants than attended Visit #1, count all participants whose answer to the question "After careful assessment of all eligibility, are you sure the participant should be randomized?" = "No".
   a. For each categorized / drop-down reason under "Select the reason(s) the participant screen-failed", produce the total count (the dietitian could enter in multiple reasons). If the reason "Other" was checked, a free-text reason will have to be entered under "If the reason for participant screen-failure is "Other", please specify:"

Demographics and Baseline Characteristics

Statistics for continuous variables are shown as median (IQR). Values for categorical variables shown as N (%).

Variable	Sub-Category	Control (n=46)	POP1 (n=100)	POP2 (n=101)	P-Value
Age (y)					
Age Group (y)	21-50				
	51-75				
Sex	Female				
	Male				
Ethnicity	Hispanic or Latino				
	Not Hispanic or Latino				
Race	Asian				
	Black or African American				
	Native Hawaiian or Other Pacific Islander				
	White				
	Other				
	Multiracial				
Marital Status	Married/Living with Partner				
	Divorced/Separated				
	Never Married				
	Widowed				
Current Employment Status	Employed full-time (40 or more hours per week)				
	Employed part time (up to 39 hours per week)				
	Homemaker				
	Retired				
	Self-employed				

DocuSign Envelope ID: 0D28DCB2-96A8-4360-9427-C8728A1D9207

Variable	Sub-Category	Control (n=46)	POP1 (n=100)	POP2 (n=101)	P-Value
	Student				
	Unable to work				
	Unemployed				
Education	High School Graduate/GED				
	Some college				
	Certificate/Trade school				
	Associate's degree				
	Bachelor's degree				
	Graduate degree				
Annual Household Income	Less than $50,000 per year				
	$50,000 to less than $100,000				
	$100,000 and above				
The number of adults (18 years or older) living in the household, including the participant (excluding adults living away from home [e.g. college students]).	1				
	2				
	3				
	4				
	5				
The number of children (less than 18 years of age) living in the household.	0				
	1				
	2				
	3				
	5				
How many people does the participant shop and cook for at home (including the participant).	1 or 2				
	3 or more				

DocuSign Envelope ID: 0D28DCB2-96A8-4360-9427-C8728A1D9207

Variable	Sub-Category	Control (n=46)	POP1 (n=100)	POP2 (n=101)	P-Value
Use of the following programs:[a]	Supplemental Nutrition Assistance Program (SNAP)				
	Section 8 Housing				
	Medicaid				
	Supplemental Security Income (SSI)				
	Food Pantry				
	None				
	Other				
Participant health insurance	Private (commercial/employer based)				
	Public (Medicare, Medicaid, Caresource, etc.)				
	Other				
	Unknown				

[a] Participants could choose more than one answer. Separate comparisons are made for each individual program.

POP1: In-Store Only Arm. POP2: In-Store and Online Arm.

Tests for differences across groups for categorical variables were conducted with the likelihood-ratio chi-square test. Test for a difference in Age across group was conducted with the Kruskal-Wallis nonparametric test.

DocuSign Envelope ID: 0D28DCB2-96A8-4360-9427-C8728A1D9207

Baseline Characteristics: Biometrics

Statistics for continuous variables are shown as median (IQR). Values for categorical variables shown as N (%).

Variable	Sub-Category	Control (n=46)	POP1 (n=100)	POP2 (n=101)	P-Value
SBP (mm Hg)					
DBP (mm Hg)					
Hypertensive Medication	No				
	Yes				
Blood Pressure Group	Normal Blood Pressure[1]				
	Elevated Blood Pressure[2]				
	Hypertension[3]				
Height (cm)					
Weight (kg)					
Body mass index (kg/m^2)					
Weight group	Underweight (<18.5 kg/m^2)				
	Normal weight (18.5-24.9 kg/m^2)				
	Overweight (25.0-29.9 kg/m^2)				
	Obese (=>30 kg/m^2)				
Waist circumference (cm)					
Hip circumference (cm)					
Waist-to-hip ratio (WHR)					
Total cholesterol (mg/dl)					
HDL cholesterol (mg/dl)					
Non-HDL cholesterol (mg/dl)					

DocuSign Envelope ID: 0D28DCB2-96A8-4360-9427-C8728A1D9207

Variable	Sub-Category	Control (n=46)	POP1 (n=100)	POP2 (n=101)	P-Value
Hypercholesterolemic medication	No				
	Yes				
Hypercholesterolemic[4]	No				
	Yes				
Triglycerides (mg/dl)[5,6]					
Glucose (mg/dl)[5]					

POP1: In-Store Only Arm. POP2: In-Store and Online Arm.

SBP: systolic blood pressure; DBP: diastolic blood pressure

Non-HDL cholesterol: Total cholesterol minus HDL cholesterol.

For conversion, 1 inch equals 2.54 centimeters. For conversion, 1 pound equals 0.453592 kilograms.

[1]Defined as SBP<120 mm Hg and DBP<80mm Hg at Visit #1
[2]Defined as SBP 120-129 mm Hg and DBP<80mm Hg at Visit #1
[3]Defined as SBP≥130 mm Hg or DBP≥80mm Hg and/or on hypertensive medication at Visit #1. This category combines stage 1 and stage 2 hypertension, as defined by the American Heart Association.
[4]Defined as % of participants with non-HDL cholesterol measurements between 130-189 mg/dl and/or on hypercholesterolemic medication at Visit #1

[5]Participants were not subjected to fasting.

[6]The Kruskal-Wallis nonparametric test was used to compare groups.

Baseline Characteristics - Activity Level & Dietary Status

Statistics are shown as N (%).

Variable	Sub-Category	Control (n=46)	POP1 (n=100)	POP2 (n=101)	P-Value
Major challenge in sticking to a diet	Busy schedule/Not enough time				
	Too expensive				
	Lack of support from family, friends, or co-workers				
	Diet too repetitive or strict				
	Lack of cooking or meal planning skills				
	Didn't see results with previous diets				
	Other				
Which best describes the participants' diet goals[a]	To lose weight				
	To manage high blood pressure				
	To manage high cholesterol or triglycerides				
	To manage diabetes				
	To reduce risk of heart disease or stroke				
	To increase energy and endurance				
	To increase intake of fruits and vegetables				
	To improve physical appearance				
	Other				
Activity Level in Daily Life	Sedentary				
	Lightly Active				
	Moderately Active				
	Very Active				
Exercise beyond daily activities	Never				
	Sometimes (1-3x a week)				
	All the time (Daily)				
Daily tobacco use (cigarettes, chew, etc)	Never				

Variable	Sub-Category	Control (n=46)	POP1 (n=100)	POP2 (n=101)	P-Value
	Sometimes (1-3x a week)				
	All the time (Daily)				
Weekly alcohol use	0				
	1-7 drinks for men and women				
	8-20 drinks for men; 8-13 drinks for women				
Previous heart attack or stroke	Yes				
	No				
Participant feels he/she is in good health.	Strongly Disagree				
	Disagree				
	Neutral				
	Agree				
	Strongly Agree				
Frequency of cooking at home	Never				
	1-2 times per week				
	3-4 times per week				
	5-6 times per week				
	Everyday				
Frequency of dining out for breakfast or brunch	Never				
	Once a month				
	1-4 times per week				
	2-3 times a month				
	5 or more times a week				
Frequency of dining out for lunch	Never				
	Once a month				
	1-4 times per week				
	2-3 times a month				

Variable	Sub-Category	Control (n=46)	POP1 (n=100)	POP2 (n=101)	P-Value
	5 or more times a week				
Frequency of dining out for dinner	Never				
	Once a month				
	1-4 times per week				
	2-3 times a month				
	5 or more times a week				
Frequency of dining out for snack foods	Never				
	Once a month				
	1-4 times per week				
	2-3 times a month				
	5 or more times a week				
Frequency of dining out for hot drinks	Never				
	Once a month				
	1-4 times per week				
	2-3 times a month				
	5 or more times a week				
Frequency of dining out for cold drinks	Never				
	Once a month				
	1-4 times per week				
	2-3 times a month				
	5 or more times a week				
Frequency of dining out for alcoholic drinks	Never				
	Once a month				
	1-4 times per week				
	2-3 times a month				
	5 or more times a week				

Variable	Sub-Category	Control (n=46)	POP1 (n=100)	POP2 (n=101)	P-Value

POP1: In-Store Only Arm. POP2: In-Store and Online Arm.

[a] Participants could choose more than one answer. Separate comparisons are made for each individual goal.

Tests for differences across groups for categorical variables were conducted with the likelihood-ratio chi-square test.

Diet: Baseline, 3 month and 6 month

Statistics for continuous variables are shown as least squares means (CI). Values for categorical variables shown as N (%).

	Control (n=46)	POP1 (n=100)	POP2 (n=101)	Intervention Effect (CI) POP1/POP2 vs. Control	P-value	Intervention Effect (CI) POP1 vs. POP2	P-value
DASH Score							
At baseline							
At 3 mon							
At 6 mon							
Change (baseline to 3 mon)							
Change (baseline to 6 mon)							
Fruit servings/1000 kcal/day							
At baseline							
At 3 mon							
At 6 mon							
Change (baseline to 3 mon)							
Change (baseline to 6 mon)							
Vegetable servings/1000 kcal/day							
At baseline							
At 3 mon							
At 6 mon							
Change (baseline to 3 mon)							
Change (baseline to 6 mon)							
Total Dairy servings/1000 kcal/day							
At baseline							
At 3 mon							
At 6 mon							
Change (baseline to 3 mon)							
Change (baseline to 6 mon)							

DocuSign Envelope ID: 0D28DCB2-96A8-4360-9427-C8728A1D9207

Low Fat servings/1000 kcal/day<br>At baseline<br>At 3 mon<br>At 6 mon<br>Change (baseline to 3 mon)<br>Change (baseline to 6 mon)							
Total Grain servings/1000 kcal/day<br>At baseline<br>At 3 mon<br>At 6 mon<br>Change (baseline to 3 mon)<br>Change (baseline to 6 mon)							
Whole Grain servings/1000 kcal/day<br>At baseline<br>At 3 mon<br>At 6 mon<br>Change (baseline to 3 mon)<br>Change (baseline to 6 mon)							
Meat servings/1000 kcal/day<br>At baseline<br>At 3 mon<br>At 6 mon<br>Change (baseline to 3 mon)<br>Change (baseline to 6 mon)							
Nuts/seeds servings/1000 kcal/day<br>At baseline<br>At 3 mon<br>At 6 mon<br>Change (baseline to 3 mon)<br>Change (baseline to 6 mon)							

Sweets servings/1000 kcal/day<br>At baseline<br>At 3 mon<br>At 6 mon<br>Change (baseline to 3 mon)<br>Change (baseline to 6 mon)							
Fats, oils servings/1000 kcal/day<br>At baseline<br>At 3 mon<br>At 6 mon<br>Change (baseline to 3 mon)<br>Change (baseline to 6 mon)							
Energy Intake -kcal/day<br>At baseline<br>At 3 mon<br>At 6 mon<br>Change (baseline to 3 mon)<br>Change (baseline to 6 mon)							
Total fat. % of kcals/day<br>At baseline<br>At 3 mon<br>At 6 mon<br>Change (baseline to 3 mon)<br>Change (baseline to 6 mon)							
Saturated Fat. % kcal/day<br>At baseline<br>At 3 mon<br>At 6 mon<br>Change (baseline to 3 mon)<br>Change (baseline to 6 mon)							

Calcium mg/1000 kcals/day<br>At baseline<br>At 3 mon<br>At 6 mon<br>Change (baseline to 3 mon)<br>Change (baseline to 6 mon)							
Potassium mg/1000 kcals/day<br>At baseline<br>At 3 mon<br>At 6 mon<br>Change (baseline to 3 mon)<br>Change (baseline to 6 mon)							
Magnesium mg/1000 kcals/day<br>At baseline<br>At 3 mon<br>At 6 mon<br>Change (baseline to 3 mon)<br>Change (baseline to 6 mon)							
Sodium mg/1000 kcals/day<br>At baseline<br>At 3 mon<br>At 6 mon<br>Change (baseline to 3 mon)<br>Change (baseline to 6 mon)							

POP1: In-Store Only Arm. POP2: In-Store and Online Arm.

A mixed model for repeated measures was used to model each outcome, controlling for age-group, sex, household size, race, income and baseline BMI. Contrasts were used to estimate differences between treatment groups a) at baseline, b) for 3-month change from baseline, and c) for 6-month change from baseline.

Blood Pressure Control: Baseline, 3 month and 6 month

Statistics for continuous variables are shown as mean (SD). Values for categorical variables shown as N (%).

Outcome	Control (n=46)	POP1 (n=100)	POP2 (n=101)	Intervention Effect (CI) POP1/POP2 vs. Control	P-value	Intervention Effect (CI) POP1 vs. POP2	P-value
**Blood pressure**[a]							
Systolic blood pressure – mm Hg							
At baseline							
At 3 mon							
At 6 mon							
Change (baseline to 3 mon)							
Change (baseline to 6 mon)							
Diastolic blood pressure – mm Hg							
At baseline							
At 3 mon							
At 6 mon							
Change (baseline to 3 mon)							
Change (baseline to 6 mon)							
**Hypertension control at 3 mon – N (%)**[b]							
SBP <120 and DBP <80							
SBP <130 and DBP <80							
SBP <140 and DBP <90							
SBP <150 and DBP <90							
**Hypertension control at 6 mon – N (%)**[b]							
SBP <120 and DBP <80							
SBP <130 and DBP <80							
SBP <140 and DBP <90							
SBP <150 and DBP <90							

DocuSign Envelope ID: 0D28DCB2-96A8-4360-9427-C8728A1D9207

POP1: In-Store Only Arm. POP2: In-Store and Online Arm.

For systolic blood pressure and diastolic blood pressure: a mixed model for repeated measures was used to model each outcome, controlling for age-group, sex, race, baseline BMI, baseline BP meds (0, 1, 2+), baseline sedentary (yes/no), and baseline current smoking (yes/no). Contrasts were used to estimate differences between treatment groups a) at baseline, b) for 3-month change from baseline, and c) for 6-month change from baseline.

For hypertension control, relative risk was calculated using logistic regression models, controlling for the baseline value of the dependent variable (e.g. SBP, DBP), age-group, sex, race, baseline BMI, baseline BP meds (0, 1, 2+), baseline sedentary (yes/no), and baseline current smoking (yes/no).

[a] Intervention effect is difference in mean change in blood pressure and 95% confidence interval.

[b]Intervention effect is the relative risk and 95% confidence interval.

Weight, BMI, WHR: Baseline, 3 month and 6 month

Statistics for continuous variables are shown as mean (SD). Values for categorical variables shown as N (%).

	Control (n=46)	POP1 (n=100)	POP2 (n=101)	Intervention Effect (CI) POP1/POP2 vs. Control	P-value	Intervention Effect (CI) POP1 vs. POP2	P-value
**Outcomes[a]**							
BMI – kg/m^2							
At baseline							
At 3 mon							
At 6 mon							
Change (baseline to 3 mon)							
Change (baseline to 6 mon)							
WHR							
At baseline							
At 3 mon							
At 6 mon							
Change (baseline to 3 mon)							
Change (baseline to 6 mon)							
**BMI control at 3 mon – N (%)[b]**							
BMI <30							
BMI <35							
BMI <40							
BMI <45							
**BMI control at 6 mon – N (%)[b]**							
BMI <30							
BMI <35							
BMI <40							
BMI <45							

DocuSign Envelope ID: 0D28DCB2-96A8-4360-9427-C8728A1D9207

POP1: In-Store Only Arm. POP2: In-Store and Online Arm.

For BMI, WHR: a mixed model for repeated measures was used to model each outcome, controlling for age-group, sex, household size, race, income, sedentary (yes/no), current smoking (yes/no), and baseline energy intake. Contrasts were used to estimate differences between treatment groups a) at baseline, b) for 3-month change from baseline, and c) for 6-month change from baseline.

For BMI control: relative risk was calculated using logistic regression models, controlling for the baseline value of BMI, age-group, sex, household size, race, income, baseline sedentary (yes/no), baseline current smoking (yes/no), and baseline energy intake.

[a] Intervention effect is difference in mean change in outcome and 95% confidence interval.

[b] Intervention effect is the relative risk and 95% confidence interval.

Labs: Baseline, 3 month and 6 month

Statistics for continuous variables are shown as mean (SD). Values for categorical variables shown as N (%).

	Control (n=46)	POP1 (n=100)	POP2 (n=101)	Intervention Effect (CI) POP1/POP2 vs. Control	P-value	Intervention Effect (CI) POP1 vs. POP2	P-value
**Outcomes**[a]							
Total Cholesterol							
At baseline							
At 3 mon							
At 6 mon							
Change (baseline to 3 mon)							
Change (baseline to 6 mon)							
NonHDL-C							
At baseline							
At 3 mon							
At 6 mon							
Change (baseline to 3 mon)							
Change (baseline to 6 mon)							
HDL-C							
At baseline							
At 3 mon							
At 6 mon							
Change (baseline to 3 mon)							
Change (baseline to 6 mon)							
Triglycerides							
At baseline							
At 3 mon							
At 6 mon							
Change (baseline to 3 mon)							
Change (baseline to 6 mon)							
**NonHDL-C control at 3 mon – N (%)**[b]							

DocuSign Envelope ID: 0D28DCB2-96A8-4360-9427-C8728A1D9207

NonHDL-C <100<br>NonHDL-C <130<br>NonHDL-C <160<br>NonHDL-C <190							
**NonHDL-C control at 6 mon – N (%)[b]**							
NonHDL-C <100<br>NonHDL-C <130<br>NonHDL-C <160<br>NonHDL-C <190							
**TG control at 3 mon – N (%)[b]**							
TG <100<br>TG <150<br>TG <200<br>TG <250							
**TG control at 6 mon – N (%)[b]**							
TG <100<br>TG <150<br>TG <200<br>TG <250							

POP1: In-Store Only Arm. POP2: In-Store and Online Arm.

For continuous measurements: a mixed model for repeated measures was used to model each outcome, controlling for age-group, sex, race, household size, income, baseline BMI, baseline cholesterol meds (no/yes), and baseline sedentary (yes/no). Contrasts were used to estimate differences between treatment groups a) at baseline, b) for 3-month change from baseline, and c) for 6-month change from baseline.

For categorical control variables: relative risk was calculated using logistic regression models, controlling for the baseline value of the dependent variable (e.g. NonHDL-C, TG), age-group, sex, household size, race, income, baseline sedentary (yes/no), baseline cholesterol meds (no/yes), and baseline BMI.

[a] Intervention effect is difference in mean change in outcome and 95% confidence interval.

[b]Intervention effect is the relative risk and 95% confidence interval.

DocuSign Envelope ID: 0D28DCB2-96A8-4360-9427-C8728A1D9207

Medication Use: Baseline and 6 month

Statistics for continuous variables are shown as mean (SD).

Variable	Control (n=46)	POP1 (n=99)	POP2 (n=102)	Intervention Effect (CI) POP1/POP2 vs. Control	P-value	Intervention Effect (CI) POP1 vs. POP2	P-value
Number of Total Medications							
Baseline							
6 mon							
Change (Baseline to 6 mon)							
Number of BP Meds							
Baseline							
6 mon							
Change (Baseline to 6 mon)							
Number of Cholesterol Meds							
Baseline							
6 mon							
Change (Baseline to 6 mon)							

POP1: In-Store Only Arm. POP2: In-Store and Online Arm.

[a] Intervention effect is difference in mean change in outcome and 95% confidence interval.

DocuSign Envelope ID: 0D28DCB2-96A8-4360-9427-C8728A1D9207

COVID Impact Analyses

The effect of the COVID-19 pandemic- represented by the US national emergency declaration date of March 13, 2020- on the primary endpoint will be evaluated through these prespecified COVID-19 impact analyses with a 15% interaction significance level. The two groups are: 1) Pre-COVID (anyone with both the baseline and 3-month dietary recall timepoints occurring before 3/13/2020) and 2) During-COVID (anyone with both the baseline and 3-month dietary recall timepoints occurring after the June 2020 recruitment re-initiation).

	Pre-COVID Intervention Effect (CI) POP1/POP2 vs. Control (N=109)	P-value	Pre-COVID Intervention Effect (CI) POP1 vs. POP2 (N=109)	P-value	During-COVID Intervention Effect (CI) POP1/POP2 vs. Control (N=138)	P-value	During-COVID Intervention Effect (CI) POP1 vs. POP2 (N=138)	P-value
DASH Score<br>-Change (baseline to 3 mon)<br>-Change (baseline to 6 mon)								
Systolic Blood Pressure<br>-Change (baseline to 3 mon)<br>-Change (baseline to 6 mon)								
Diastolic Blood Pressure<br>-Change (baseline to 3 mon)<br>-Change (baseline to 6 mon)								
BMI<br>-Change (baseline to 3 mon)<br>-Change (baseline to 6 mon)								
Non-HDL-C<br>-Change (baseline to 3 mon)<br>-Change (baseline to 6 mon)								
Triglycerides<br>-Change (baseline to 3 mon)<br>-Change (baseline to 6 mon)								

Subgroups of Interest: Primary Endpoint at 3 Mon

Subgroup	POP1/POP2	Control	Absolute Mean DASH Difference (compared to Control)
Age<br>21-50<br>51-75			
Gender<br>Male<br>Female			
Race<br>White<br>Not White			
Married<br>Yes<br>No			
Education<br>Bachelor/Graduate<br>Not			
Employment<br>Fulltime<br>Not Fulltime			
Annual Household Income<br>≥$100,000<br>Less			
Household Size<br>Single Person<br>More			
Frequency of Cooking at Home<br>≥5-6x/week<br>Less			
Activity level<br>Moderate/very<br>Less			
Hypercholesterolemia<br>Yes<br>No			
Hypertension<br>Yes<br>No			
Obese			

Yes<br>No			
Baseline DASH Score<br>Above ≥40<br>Below 40			

DocuSign Envelope ID: 0D28DCB2-96A8-4360-9427-C8728A1D9207

Food Literacy: Baseline and Follow-up

Questions (all questions using a 5-point Likert scale)	Control (n=46)	POP1 (n=100)	POP2 (n=101)	Intervention Effect (CI), POP1/POP2 vs. Control	P-value	Intervention Effect (CI), POP1 vs. POP2	P-value
Cook dinner at home?<br>Baseline<br>Followup<br>Change from baseline<br>(5 choices, Highest "everyday")							
In the past month, how often did you dine out for breakfast or brunch?<br>Baseline<br>Followup<br>Change from baseline<br>(5 choices, ≥5x/week)							
In the past month, how often did you dine out for lunch?<br>Baseline<br>Followup<br>Change from baseline<br>(5 choices, ≥5x/week)							
In the past month, how often did you dine out for dinner?<br>Baseline<br>Followup<br>Change from baseline<br>(5 choices, ≥5x/week)							

Questions (all questions using a 5-point Likert scale)	Control (n=46)	POP1 (n=100)	POP2 (n=101)	Intervention Effect (CI), POP1/POP2 vs. Control	P-value	Intervention Effect (CI), POP1 vs. POP2	P-value
In the past month, how often did you dine out for snack foods?<br>Baseline<br>Followup<br>Change from baseline<br>(5 choices, ≥5x/week)							
In the past month, how often did you dine out for hot drinks?<br>Baseline<br>Followup<br>Change from baseline<br>(5 choices, ≥5x/week)							
In the past month, how often did you dine out for cold drinks?<br>Baseline<br>Followup<br>Change from baseline<br>(5 choices, ≥5x/week)							
In the past month, how often did you dine out for alcoholic drinks?<br>Baseline<br>Followup<br>Change from baseline<br>(5 choices, ≥5x/week)							
Eat dinner at home with other members of your household?<br>Baseline<br>Followup<br>Change from baseline<br>(5 choices, Highest “everyday”)							

DocuSign Envelope ID: 0D28DCB2-96A8-4360-9427-C8728A1D9207

Participant Feedback (Part 1)

Statistics are median score with IQR.

Questions (all are questions using a 5-point Likert scale)	Control (n=46)	POP1 (n=99)	POP2 (n=102)
I had adequate time with the dietitian during the counseling visit.			
The DASH goals that I set with the dietitian were reasonable.			
The information provided on food portions made me aware of how much I should eat from each food group.			
The dietitian counseling session helped me understand the link between what I eat and my health.			
The action plan that I set with the dietitian helped me to meet my DASH goals.			
The dietitian provided useful ways to help me meet my DASH goals.			
The dietitian provided information in a way I could easily understand.			
The dietitian suggested useful ways I could increase fruits, vegetables and low-fat dairy foods in my life.			
The dietitian provided me with tools that I could use to make my eating healthier.			
The amount of information provided to me during the dietitian counseling session was just right.			
The handouts provided to me during the study helped me eat healthier.			
My Food Purchasing Snapshot was easy to understand.	NA		
My Food Purchasing Snapshot helped me to shop more healthfully.	NA		
I feel I had adequate time with the dietitian during the in-store shopping experience with online enhancement.	NA		
The dietitian-led store tours with online enhancement helped me identify what information on a food label is most important to understand for my health.	NA		
The information I received on how to make healthy food substitutions was clear.	NA		
The dietitian-led store tours with online enhancement provided me with strategies to better select DASH-friendly foods.	NA		
The dietitian-led store tours with online enhancement provided me with adequate knowledge on how to modify recipes to reduce sodium content.	NA		

DocuSign Envelope ID: 0D28DCB2-96A8-4360-9427-C8728A1D9207

Questions (all are questions using a 5-point Likert scale)	Control (n=46)	POP1 (n=99)	POP2 (n=102)
Report mean (SD) for each cell.			
The dietitian-led store tours with online enhancement helped me identify easy ways to plan for lower sodium eating.	NA		
The dietitian-led store tours with online enhancement informed me how to save money when buying nutritious fruits and vegetables.	NA		
The dietitian-led store tours with online enhancement taught me ways to increase fruit and vegetable servings at meals and snacks.	NA		
The dietitian-led store tours with online enhancement helped me identify unique ways to prepare fruits and vegetables in the home.	NA		
The dietitian-led store tours with online enhancement guided me in finding new ways to incorporate fruits and vegetables into favorite family dishes.	NA		
The dietitian showed me ways to fit increased low-fat dairy consumption into my life.	NA		
The dietitian-led store tours with online enhancement helped me understand what a dairy serving looks like.	NA		
The dietitian-led store tours with online enhancement helped clear up my confusion surrounding dietary fats.	NA		
Report mean (SD) for each cell.			

DocuSign Envelope ID: 0D28DCB2-96A8-4360-9427-C8728A1D9207

Participant Feedback (Part 2)

Results shown as N (%)

Questions	POP1 (n=99)	POP2 (n=102)
How many more point-of-purchase sessions would you have wanted to have (4 choices)?<br>None<br>1-2<br>3-4<br>5-6<br>(only one can be selected)		
Would it be helpful to continue to receive updated Food Purchasing Snapshots?<br>No<br>Yes, through a website<br>Yes, through an app<br>Yes, by paper copies in the mail<br>(select all that apply)		
Would it be helpful to continue to get support from the dietitian in the future?<br>No<br>Yes, through a website<br>Yes, through an app<br>Yes, through additional visits<br>(select all that apply)		

APPROVAL – SIGNATURES

Name: Dylan Steen

Signature: DocuSigned by: Dylan Steen
FE93FC89BCFD4A6...

Date: 12/2/2021

Name: Sarah Couch

Signature: DocuSigned by: Sarah Couch
B604E6D9699843C...

Date: 12/1/2021

Name: Eileen King

Signature: DocuSigned by: Eileen G King
430FAABAA3FE482...

Date: 12/1/2021

Name: Matthew C. Fenchel

Signature: DocuSigned by: Matthew C. Fenchel
13AC15995FA1487...

Date: 12/1/2021
