# Supplementary material for: Efficacy of supermarket and web-based interventions for improving dietary quality: a randomized, controlled trial
Source: Nat Med. 2022 Dec 1;28(12):2530–6. doi: 10.1038/s41591-022-02077-7 (PMC9800276; doi:10.1038/s41591-022-02077-7)
Supplement: Supplementary file 2 — Reporting Summary [file 41591_2022_2077_MOESM2_ESM.pdf]

nature portfolio

Corresponding author(s): Dylan L. Steen MD MS

Last updated by author(s): Sep 17, 2022

Reporting Summary

Nature Portfolio wishes to improve the reproducibility of the work that we publish. This form provides structure for consistency and transparency in reporting. For further information on Nature Portfolio policies, see our Editorial Policies and the Editorial Policy Checklist.

Statistics

For all statistical analyses, confirm that the following items are present in the figure legend, table legend, main text, or Methods section.

n/a	Confirmed	
☐	☒	The exact sample size (*n*) for each experimental group/condition, given as a discrete number and unit of measurement
☐	☒	A statement on whether measurements were taken from distinct samples or whether the same sample was measured repeatedly
☐	☒	The statistical test(s) used AND whether they are one- or two-sided<br>*Only common tests should be described solely by name; describe more complex techniques in the Methods section.*
☐	☒	A description of all covariates tested
☐	☒	A description of any assumptions or corrections, such as tests of normality and adjustment for multiple comparisons
☐	☒	A full description of the statistical parameters including central tendency (e.g. means) or other basic estimates (e.g. regression coefficient) AND variation (e.g. standard deviation) or associated estimates of uncertainty (e.g. confidence intervals)
☐	☒	For null hypothesis testing, the test statistic (e.g. *F*, *t*, *r*) with confidence intervals, effect sizes, degrees of freedom and *P* value noted<br>*Give P values as exact values whenever suitable.*
☒	☐	For Bayesian analysis, information on the choice of priors and Markov chain Monte Carlo settings
☐	☒	For hierarchical and complex designs, identification of the appropriate level for tests and full reporting of outcomes
☒	☐	Estimates of effect sizes (e.g. Cohen's *d*, Pearson's *r*), indicating how they were calculated

Our web collection on statistics for biologists contains articles on many of the points above.

Software and code

Policy information about availability of computer code

Data collection	Completed dietary recalls were analyzed for average calories, nutrients, and food group servings using the Nutrition Data System for Research (Nutrition Coordinating Center, University of Minnesota, Minneapolis, MN), versions 2018 and 2019. Other data were collected by Research Electronic Data Capture (REDCap).
Data analysis	SAS 9.4 TS1M5 (SAS, Inc., Cary, NC) software was used for all analyses.

For manuscripts utilizing custom algorithms or software that are central to the research but not yet described in published literature, software must be made available to editors and reviewers. We strongly encourage code deposition in a community repository (e.g. GitHub). See the Nature Portfolio guidelines for submitting code & software for further information.

Data

Policy information about availability of data

All manuscripts must include a data availability statement. This statement should provide the following information, where applicable:

- Accession codes, unique identifiers, or web links for publicly available datasets
- A description of any restrictions on data availability
- For clinical datasets or third party data, please ensure that the statement adheres to our policy

The datasets generated during and/or analyzed during the current study are not publicly available, but may be made available upon reasonable request to the corresponding author.

Human research participants

Policy information about studies involving human research participants and Sex and Gender in Research.

Reporting on sex and gender	The gender of participants was determined during an in-person study visit conducted by the study dietitian. Gender was self-reported. We have reported our findings by gender.
Population characteristics	Men and women between ages 21 to 75 years were eligible for inclusion if they were the primary food planner for their household, were an existing shopper at one of the study Kroger supermarkets, were able to shop and prepare food independently, and had a home computer. Participants were enrolled if they had at least one cardiovascular (CV) risk factor: 1) systolic blood pressure (SBP) >130 mm Hg, diastolic blood pressure (DBP) >80 mm Hg, and/or treatment with an anti-hypertensive medication; 2) obesity defined as a body-mass index (BMI) ≥30 kg/m2; and/or 3) non-high-density lipoprotein cholesterol (non-HDL-C) ≥130 mg/dl and/or treatment with a lipid-lowering medication. Key exclusion criteria included current treatment with another dietary or weight loss intervention, use of Kroger's online shopping platform within 12 months, prior use of Kroger's dietary counselling services, baseline SBP ≥190 mm Hg, DBP ≥110 mm Hg, or non-HDL-C ≥190 mg/dl.
Recruitment	All participants had to have a primary care clinician at UC Health. Lists of UC Health patients likely to meet the eligibility criteria (e.g., diagnosis code of hypertension) were generated from UC Health's Clarity Database (Epic Systems Corporation). The UC study coordinator then mailed study materials to those patients living near the study stores. Phone calls, texting, emails, and flyers were also used. Interested patients were phone screened by the coordinator, and if eligible, were entered into the run-in period. Following a run-in period consisting of collecting baseline dietary intake via phone and survey information via email, participants attended a study visit at their assigned store location. All study visits were conducted in the store by a supermarket registered dietitian ("study dietitian"). At the end of visit, once eligibility and interest were confirmed, participants were randomized.<br><br>There is self-selection bias in dietary clinical trials which aim to modify dietary behavior. Participants must be interested in participating in a study as well as modifying their diets. Our findings likely do not apply to those who would not want to participate in a study, meet with a dietitian, attend educational visits, or try improve their current eating habits and health.
Ethics oversight	The study was coordinated by the University of Cincinnati (UC) and the UC IRB approved the protocol.

Note that full information on the approval of the study protocol must also be provided in the manuscript.

Field-specific reporting

Please select the one below that is the best fit for your research. If you are not sure, read the appropriate sections before making your selection.

☒ Life sciences ☐ Behavioural & social sciences ☐ Ecological, evolutionary & environmental sciences

For a reference copy of the document with all sections, see nature.com/documents/nr-reporting-summary-flat.pdf

Life sciences study design

All studies must disclose on these points even when the disclosure is negative.

Sample size	267 participants were randomized. Analysis cohort included 247 randomized participants. We estimated that 100 participants in each intervention group (total = 200) and 50 participants for the control group would be required to provide 93% and 97% power to detect a 5-point difference in change from baseline to 3 months in the DASH score for the first and second hypothesis tests, respectively. Power estimates were performed at a two-sided alpha=0.05 and assumed equal standard deviations (SD)=9 for the primary endpoint.
Data exclusions	20 participants were withdrawn from study participation (due to inability to deliver study visits) by study leadership when the U.S. declared a national emergency on March 13, 2020.
Replication	Replication is possible, but has not been performed by any other study team due to feasibility challenges.
Randomization	Participants were randomized 2:2:1 to Strategy 1 (N=107), Strategy 2 (N=109), and Control (N=51). In the analysis cohort, Strategy 1, Strategy 2, and Control had 100, 101, and 46 participants, respectively. Randomization was accomplished by the study dietitian using the pre-loaded, stratified randomization list in the Research Electronic Data Capture (REDCap) randomization module, which programmatically displayed the assignment. Randomization was stratified by characteristics reported to influence food choice: age (2 levels), gender (2 levels), and household size (3 levels). Block size was 5.
Blinding	The study coordinators and investigators had access to the REDCap database which contained each participants treatment assignment. The investigators were unblinded to group assignment. However, all calculations of DASH score (the primary endpoint) during follow-up and changes in DASH score were only performed after completion of the study. Thus, the study investigators were blinded to all changes in the primary endpoint.

Reporting for specific materials, systems and methods

We require information from authors about some types of materials, experimental systems and methods used in many studies. Here, indicate whether each material, system or method listed is relevant to your study. If you are not sure if a list item applies to your research, read the appropriate section before selecting a response.

Materials & experimental systems

n/a	Involved in the study
☒	☐ Antibodies
☒	☐ Eukaryotic cell lines
☒	☐ Palaeontology and archaeology
☒	☐ Animals and other organisms
☐	☒ Clinical data
☒	☐ Dual use research of concern

Methods

n/a	Involved in the study
☒	☐ ChIP-seq
☒	☐ Flow cytometry
☒	☐ MRI-based neuroimaging

Clinical data

Policy information about clinical studies

All manuscripts should comply with the ICMJE guidelines for publication of clinical research and a completed CONSORT checklist must be included with all submissions.

Clinical trial registration

Registration number and registry name: NCT03895580 (Clinicaltrials.gov)

Study protocol

The study protocol has been included in the submission.

Data collection

All participant visits were conducted across 13 Kroger supermarket locations in Ohio and Kentucky. Each of these locations had a Kroger clinic which allowed for study visits and assessments. From March 2019 through the end of February 2021, 267 participants in total were randomized.

Outcomes

The primary endpoint was change in DASH score. DASH score was calculated on a 0 to 90-point scale, with a higher score indicating greater adherence to a DASH diet. In this trial, DASH score was calculated from 11 component scores based on the alignment between actual intake and the DASH serving recommendations (e.g., whole grains, vegetables, fruits). At baseline, 3 months, and 6 months, three 24-dietary recalls (two weekday and one weekend) were collected at each timepoint by phone. DASH scores were calculated from these raw dietary intake data. DASH scores were first calculated for individual recalls followed by calculation of a participant's mean DASH score at each timepoint. DASH scores were not calculated until all the study dietary recalls were completed (i.e., blinding not an issue). Use of dietary intake recalls, collection on multiple days, and a comprehensive scoring system to reflect the multiple components of DASH were used to optimize measurement of DASH dietary pattern adherence. The prespecified secondary endpoints of systolic blood pressure, diastolic blood pressure, body-mass index, total cholesterol, non-high-density cholesterol, and triglycerides were measured during study visits by the study dietitians and analyzed for changes at 3 months and 6 months. All laboratory studies were performed using a Cardiocheck Plus analyzer (PTS Diagnostics, Whitestown, IN) on a fingerstick blood sample. Blood pressure measurements were performed using an automated device (Omron Blood Pressure Monitor, BP760N; Omron Healthcare, Kyuto, Japan). COVID-19 impact analyses were prespecified prior to database lock. The pre-pandemic subgroup was defined as those participants who were randomized and had completed 3 months of follow-up prior to study cessation when the U.S. declared a national emergency on March 13, 2020. The statistical analysis plan (SAP) has been included in the submission.
